# Body‐worn cameras’ effects on police officers and citizen behavior: A systematic review

**DOI:** 10.1002/cl2.1112

**Published:** 2020-09-09

**Authors:** Cynthia Lum, Christopher S. Koper, David B. Wilson, Megan Stoltz, Michael Goodier, Elizabeth Eggins, Angela Higginson, Lorraine Mazerolle

**Affiliations:** ^1^ George Mason University Fairfax Virginia USA; ^2^ University of Queensland Brisbane Australia; ^3^ Queensland University of Technology Brisbane Australia

## 1 PLAIN LANGUAGE SUMMARY

### 1.1 Body‐worn cameras (BWCs) do not have clear or consistent effects on most officer or citizen behaviors, but different practices need further evaluation

Law enforcement agencies have rapidly adopted BWCs in the last decade with the hope that they might improve police conduct, accountability, and transparency, especially regarding use of force.

Overall, there remains substantial uncertainty about whether BWCs can reduce officer use of force, but the variation in effects suggests there may be conditions in which BWC could be effective. BWCs also do not seem to affect other police and citizen behaviors in a consistent manner, including officers’ self‐initiated activities or arrest behaviors, dispatched calls for service, or assaults and resistance against police officers. BWCs can reduce the number of citizen complaints against police officers, but it is unclear whether this finding signals an improvement in the quality of police–citizen interactions or a change in reporting.

Research has not directly addressed whether BWCs can strengthen police accountability systems or police–citizen relationships.

***What is the aim of this review?***

*This Campbell systematic summarizes the evidence from 30 studies of the effects of BWCs on several officer and citizen behaviors. The majority of studies are from the United States.*

### 1.2 What is this review about?

The last decade has been marked by the rapid adoption of BWCs by the police and a growing body of evaluation research on the technology's effects. Spurred on by high‐profile officer‐involved shooting incidents and protests, many citizens and community groups have supported the adoption of BWCs, hoping that this technology will deter police misconduct, better capture use‐of‐force events, and increase police accountability and transparency.

At the same time, some police officers and community members have expressed concerns that BWCs might discourage citizens from reporting crimes or cause officers to pull back on preventative or proactive activities that may help prevent offending. This Campbell systematic review synthesizes research on the impacts of BWCs on officer and citizen behaviors.

### 1.3 What studies are included?

Studies eligible for this review included those that examined the use of BWCs by law enforcement officers using either randomized controlled trials (RCTs) or quasi‐experimental research designs, and that measured police or citizen behaviors, rather than their perceptions. All studies compared officers wearing BWCs with officers not wearing BWCs.

Thirty eligible studies were found, which reported on 12 different types of outcome measures of officer or citizen behavior. A total of 116 effects on these outcomes are examined. Almost all studies were carried out in a single municipal jurisdiction (e.g., a city or county). The majority of studies take place in the United States.

### 1.4 What are the findings of this review?

Overall, the way BWCs are currently being used may not substantially affect most officer or citizen behaviors. The use of BWCs does not have consistent or significant effects on officers’ use of force, arrest activities, proactive or self‐initiated activities, or other measured behaviors. Nor do BWCs have clear effects on citizens’ calls to the police or assaults or resistance against officers.

Analysis suggests that restricting officer discretion in turning on and off BWCs may reduce police use of force, but more assessment is needed.

BWCs may reduce the number of citizen complaints against police officers, although it is unclear why complaints decline.

### 1.5 What do the findings of this review mean?

BWCs are one of the most rapidly diffusing and costly technologies used by police agencies today. This review questions whether BWCs bring the expected benefits to the police and their communities.

Existing research does not evaluate whether police accountability or police–citizen relationships are strengthened by BWCs. Much more knowledge is needed about when BWCs do create desired effects, and whether they are cost‐effective.

For the many police agencies that have already purchased BWCs, researchers should continue testing for ways in which both police and citizens might gain benefits from the cameras’ continued use. These could include limiting the discretion that officers have with BWC use, using BWCs for coaching, training or evidentiary purposes, and finding ways that BWCs can be used to strengthen police–citizen relationships, internal investigations, or accountability systems.

### 1.6 How up‐to‐date is this review?

This review includes studies completed and available in written form as of September 2019.

## 2 EXECUTIVE SUMMARY/ABSTRACT

### 2.1 Background

In the past decade, many communities have experienced high‐profile police‐involved shootings and deaths in custody, as well as citizen protests and demands for greater police accountability and transparency. These events have helped spur the rapid adoption of BWCs by law enforcement agencies, with the expectation that cameras might improve police conduct, accountability, and transparency, especially regarding use of force. At the same time, both police and community leaders have expressed privacy concerns about cameras and fears that BWCs might discourage citizens from reporting crimes or cause officers to pull back on their duties. Such expectations and concerns, in the face of the rapid adoption of this technology, have been met with significant levels of research and evaluation of BWCs’ effects to better inform decisions about BWC purchases and use.

### 2.2 Objectives

The objective of this Campbell systematic review is to synthesize the evaluation research on the impacts of BWCs on several officer and citizen behaviors, including officer use of force, citizen complaints against officers, arrest, assaults/resistance against officers, dispatched calls for service, officer self‐initiated calls, pedestrian and traffic stops, and other behaviors.

### 2.3 Search methods

This review applied a systematic search strategy to the Global Policing Database (GPD) from 2004 to December 2018, which contains all published and unpublished experimental and quasi‐experimental evaluations of policing interventions conducted since 1950. The GPD search was supplemented by an additional search to obtain studies of BWCs from January 2019 to September 2019.

### 2.4 Selection criteria

Experimental and quasi‐experimental designs were eligible for this review. Additionally, studies must have examined the use of BWCs by law enforcement officers and measured police or citizen behaviors (rather than their perceptions).

### 2.5 Data collection and analysis

In total, 30 independent studies were found across 35 eligible documents coded for this review. From these 30 studies, 116 effect sizes were coded across 12 outcome measures of officer or citizen behaviors. Inverse‐variance weighted random‐effects meta‐analysis was used to synthesize the effect sizes. The effect size used was the relative incident rate ratio (RIRR). Results on this effect size were transformed into a mean percent increase or decrease (change) in treatment condition relative to the control condition for the counts associated with each outcome. Risks of bias, adopted from the Cochrane risk‐of‐bias tool (Sterne et al., 2019), were recorded at both the study and outcome levels. There were no widespread violations of the randomization process or missing data in the studies examined. Depending on the particular outcome measured, however, the measurement or ascertainment of the outcome could have differed between intervention groups, and some outcomes likely suffered from bias risk. The risk of contamination bias was also likely in many studies.

### 2.6 Results

Findings from this Campbell systematic review indicate that BWCs can reduce the number of citizen complaints against police officers (% change = −16.6, 95% confidence interval [CI] [−30.0 to −0.7]), although it remains unclear whether this finding signals an improvement in the quality of police–citizen interactions or a change in reporting. The current evidence is insufficient for concluding that BWCs reduce officer use of force (% change = −6.8, 95% CI [−19.5 to 7.9]), but there remains substantial uncertainty in this effect (moderator analyses suggest that BWCs may be more likely to reduce police use of force if agencies highly restrict officers’ discretion in how they use the cameras). BWCs do not seem to affect other police and citizen behaviors (or to do so in a consistent manner), including officers’ arrest behaviors (% change = −3.9, 95% CI [−12.7 to 5.8] and self‐initiated activities (% change = 3.8, 95% CI [−5.2 to 13.5]), dispatched calls for service (% change = 2.6, 95% CI [−3.0 to 8.6]), and assaults or resistance against police officers (% change = 15.9, 95% CI [−4.9 to 41.3]). There is high variability in findings across studies, which suggests that BWCs can have positive, negative, or null impacts on police or citizen behaviors under different circumstances that are not well understood.

It seems that overall, however, the expectations that BWCs might change officer or citizen behaviors (for better or worse) have not yet been consistently realized. Research has not addressed whether BWCs can increase police accountability or police–citizen relationships more generally.

### 2.7 Author's conclusions

BWCs are one of the most rapidly diffusing and costly technologies recently adopted by police agencies. However, citizens’ and police leaders’ expectations about the impacts of this technology have not always been realized, thus raising questions as to whether the current use of BWCs brings expected benefits to agencies and their communities. It is unclear how or why BWCs reduce complaints against the police, and the existing research does not speak to whether police accountability or police–citizen relationships are strengthened by BWCs. For the many police agencies that have already purchased BWCs, researchers should continue testing for ways in which both police and citizens might gain benefits from the cameras’ continued use. These methods might include limiting the discretion that officers have with BWC use, using BWCs in training or for evidentiary purposes, or finding ways that BWCs can be used to strengthen police–citizen relationships.

## 3 BACKGROUND

### 3.1 The rapid diffusion of BWCs into policing and society

BWCs—also called body‐worn videos—are small video and audio recording devices that law enforcement officers wear on their clothing or glasses. These cameras can be turned on manually or automatically based on a variety of procedures, policies, rules, or prompts that are determined by an agency, government, or another municipal oversight group. When operating correctly and barring mishaps, BWCs can visually and audibly record interactions, activities, and events from an officer's vantage point (cameras worn by officers point outward, not inward on officers). Many cameras can also record a small time period before and after the cameras are activated, to capture a wider time frame around events that officers choose to record. Given these capabilities, BWCs are believed to provide an additional and more objective record of events involving officers and members of the community than written reports or accounts by officers or citizens alone.

BWCs have been in use since the 2000s, beginning with early trials by police agencies in the United Kingdom and also Australia (Taylor, 2016; although prior to BWCs, police used vehicle dashboard cameras, which recorded officer and citizen behavior on traffic stops). Today, BWCs are likely the most rapidly diffusing technologies in modern police history. Although it is difficult to determine how many BWCs are in circulation today, there have been some estimates. In the United Kingdom, one assessment by a privacy watchdog group found that over 70% of police forces had acquired cameras by 2019 and were rapidly moving toward full adoption.^1^ In the United States, the most recent adoption estimates provided by the Bureau of Justice Statistics indicate that as of 2016, 60% of local police departments and 49% of sheriff's offices had fully deployed their BWCs (Hyland, 2018). This reflects a near doubling of BWC use since 2013 (Bureau of Justice Statistics, 2013). Hyland also notes that by 2016, 86% of general‐purpose law enforcement agencies that had acquired BWCs had a formal policy in place or under development, signifying that agencies are also institutionalizing this technology into their general operations. At the time of this publication, the level of adoption of BWCs in the United States is likely even higher, with more officers wearing BWCs on a regular basis. It would not be an exaggeration to say that when encountering a uniformed police officer, persons in the United States and the United Kingdom would likely encounter one who would be recording their interaction with a body‐worn video device.

In the United States, the recent and continually unfolding history of the rapid adoption of BWCs in the past decade (the 2010s) provides clues as to what both police and citizens expect cameras to accomplish. The push for BWC adoption has been propelled by highly publicized and filmed events involving (often) White police officers killing (often) unarmed Black individuals (see general discussions by Braga, Sousa, Coldren, & Rodriguez, 2018; Lum, Stoltz, Koper, & Scherer, 2019; Maskaly, Donner, Jennings, Ariel, & Sutherland, 2017; Nowacki & Willits, 2018; White, 2014). The first significant event of this era did not actually involve a police officer or a BWC, but an armed individual who, posing as a neighborhood watchman, killed an unarmed Black youth—Travon Martin—in 2012. Following the Martin killing was the shooting of Michael Brown in 2014 by a Ferguson, Missouri police officer and then the death of Freddie Gray while in the custody of police in Baltimore City, Maryland, in 2015. These and many other sentinel events made national headlines as they were captured on citizens’ cell phone cameras.

These events sparked significant protest and reform movements, most notably Black Lives Matter,^2^ that called for substantial reforms and greater accountability and transparency of the police, especially to their uses of force, misconduct, and in some cases, crimes. During this time, other policing tactics also were heavily scrutinized and challenged in court, especially the widespread use of stop‐question‐and‐frisk (see, e.g., *Floyd et al. vs. New York City et al.,* 08 Civ. 1034 [SAS]). These and other long‐brewing concerns about police tactics, accountability, and use of force led to a significant review of policing undertaken by President Obama's Task Force on 21st Century Policing (2015), which considered BWCs as one possible option to reduce police use of force and improve police accountability and transparency with the public. In culmination, these contexts fostered enough public protest and political will to generate an urgent call for the adoption of BWCs. This demand was matched with a prepared supplier; technology companies had already developed both BWCs and other similar surveillance devices (e.g., in‐car cameras, license plate readers, and closed‐circuit televisions). The U.S. Department of Justice in 2015 also provided $20 million in funds to support BWC adoption (U.S. Department of Justice, 2015), which fueled their rapid uptake.^3^ Further, national civil rights groups such as the Leadership Conference on Civil and Human Rights^4^ also expressed support for cameras, while at the same time emphasizing that regulations for camera use should be put in place to both protect citizens and increase police accountability (Leadership Conference on Civil and Human Rights & Upturn, 2017[updated]).

Thus, BWCs—in a period of less than a decade—became one of the most rapidly adopted law enforcement technologies in the history of modern policing. Given the rapid and widespread implementation of BWCs, their costs, and the high expectations that both citizens and police leaders had for them, an essential question for practitioners, government officials, researchers, and citizens is whether the cameras effectively achieve these expectations. In their narrative review of empirical BWC research, Lum et al. (2019) suggested that BWCs have not had consistent effects on the behaviors of officers or citizens, for better or worse, and that both citizens and the police seem to believe that BWCs might be able to protect each from the other. Others, however, have been more optimistic in their assessments (see, e.g., Gaub & White, 2020; Malm, 2019; Maskaly et al., 2017). Unlike all of these previous reviews and commentaries, this systematic review of BWC evaluation research seeks to examine and synthesize BWC research outcomes more specifically using meta‐analysis techniques.

### 3.2 The intervention and how it might work

While there is little debate about how BWCs technically operate, there is more debate about how BWCs affect (or are expected to affect) officer and citizen behaviors. The diffusion story of BWCs, at least in the United States, seems clear: BWCs were intended to document interactions between police and citizens to increase the transparency and accountability of these interactions, especially during investigations of police misconduct. These expectations were laid out by both President Obama's *Task Force on 21st Century Policing Report* (2015, pp. 31–32) as well as the *Civil Rights Principles on Body Worn Cameras* developed by the Leadership Conference on Civil and Human Rights (2015, see principle 4). Researchers have also found that the public supported the adoption of BWCs because cameras might more generally improve police performance and behavior and reduce excessive uses of force (see Crow, Snyder, Crichlow, & Smykla, 2017; Culhane, Bowman, & Schweitzer, 2016; Ellis, Jenkins, & Smith, 2015; Sousa, Miethe, & Sakiyama, 2018), although citizen support may be contingent on an individual's race or background (see Crow et al., 2017; Kerrison, Cobbina, & Bender, 2018; Sousa et al., 2018), or personal beliefs and involvement in social institutions (Miethe, Liberman, Heen, & Sousa, 2019). It is important to note the nuanced differences in some of these expectations by citizens and communities. Generally, people believed that cameras could reduce police use of force (and also disparate use of force), and improve officer behavior toward citizens. But there was also the expectation that camera footage could be used to increase the accountability of the police in specific incidents as well.

On the other hand, police feelings and beliefs about BWCs may differ from those of citizens. Survey research on officers’ attitudes toward BWCs indicate that officers either have—or grow to have—positive attitudes toward cameras once they start using them (Ellis et al., 2015; Fouche, 2014; Gaub, Todak, & White, 2018; Grossmith et al., 2015; Jennings, Fridell, & Lynch, 2014; Jennings, Lynch, & Fridell, 2015; Koen, 2016; McLean, Wolfe, Chrusciel, & Kaminski, 2015; Smykla, Crow, Crichlow, & Snyder, 2015; Toronto Police Service, 2016; White, Todak, & Gaub, 2018). This research is reviewed in Lum et al. (2019), but in summary, it seems that the most likely reason that officers have positive feelings for BWCs is that officers see cameras as a means for protecting themselves from frivolous complaints or one‐sided stories about their conduct (Fouche, 2014; Goetschel & Peha, 2017; Koen, 2016; McLean et al., 2015; Owens & Finn, 2018; Pelfrey & Kenner, 2016). As Braga, Barao, Zimmerman, Douglas, and Sheppard (2019) note, “BWC videos reflect the officers’ gaze, and can serve to counter narratives recorded on smartphones by members of the public, and potentially reduce organizational liability” (p. 22). In the eyes of officers, BWCs “work” because they deter citizen misbehavior and keep citizens accountable. Survey findings also indicate that some officers are skeptical about whether BWCs will actually change their own behavior (Headley, Guerette, & Shariati, 2017; Pelfrey & Kenner, 2016). BWCs are also viewed by officers as a valuable evidentiary gathering tool that can aid in the investigations of crimes. These incongruences between the expectations of officers and citizens about how BWCs might work complicates our interpretation of the effects of BWCs.

Whether one believes BWCs keep citizens or the police accountable, the hypothesized mechanism of BWCs’ effects is the self‐awareness generated when an individual is being recorded and watched, which may deter wrongdoing or socially undesirable behavior because cameras may increase a person's perceived risk of detection (Ariel, Farrar, & Sutherland, 2015). For example, BWCs are theorized to have a deterrent effect on excessive use of force or unlawful actions by officers because officers will be aware that they are being recorded, which leads them to exercise restraint. This assumes that officers actively remember that they are wearing cameras or are being recorded by another officer's camera (both assumptions may not always be the case). Similarly, BWCs may also deter the citizens that officers encounter. For example, civilians may see the cameras (or be alerted to them verbally by officers) and moderate their behavior accordingly because they become aware that they are being recorded. Again, this hypothesis assumes that citizens even notice or are aware that officers are recording them. McClure et al., (2017), Goodison and Wilson (2017), and White, Todak, and Gaub (2017) all found that citizens more often than not did not remember if officers were wearing cameras.

In likely the first RCT of the effects of BWCs, Ariel et al. (2015; see also Farrar, 2012; Farrar & Ariel, 2013) use theoretical foundations of self‐awareness (Duval & Wicklund, 1972; Wicklund, 1975), socially desirable responding (see Paulhus, 1984), and deterrence (Nagin, 2013) to argue that BWCs can deter what is perceived as socially unacceptable behavior by increasing an individual's “knowing with sufficient certainty that our behavior is being observed” (p. 516). Ariel et al. (2017) hypothesized “that the self‐awareness that arises when we are aware of being watched/filmed drives us to comply with rules/norms, primarily because of the perceived certainty of punishment” (p. 297). Ariel et al. (2018) also apply these concepts to explain why officers may be *more likely* to be assaulted when wearing BWCs. They argue that officers may become overly‐deterred and excessively self‐conscious, which could hamper their ability to take control of a situation, thereby increasing the chances that they will be assaulted.

Many measures have been used to examine these theorized impacts of BWCs for both officers and citizens, although it may be challenging to disentangle upon whom the self‐awareness and subsequent deterrence effect is operating. For example, the reduction in use of force is one common measure researchers have used to examine the deterrent impacts of BWCs on officers. However, a reduction in the use of force by an officer wearing a BWC could also reflect the restraint of a citizen (which in turn tempers the officer's potential use of force), if he or she is aware of being recorded. Another common measure used to evaluate the deterrent impact of BWCs on officer behavior is the reduction in complaints against police officers. However, a reduction in complaints might also reflect a deterrent effect on citizens (or even a reporting effect). If, for example, citizens know they are being recorded or are shown a video of their encounter after they threaten to file a complaint, they may feel corrected, embarrassed, or deterred from continuing with their complaint (regardless of whether their complaint was objectively justified).

The concept of self‐awareness and subsequent deterrence need not only apply to wrongdoing. Officers have a great deal of discretion in terms of whether they arrest or cite individuals or write certain reports. For example, there may be legitimate reasons why an officer might not arrest an individual who has broken the law. Since BWCs are recording officer actions, officers might not want to risk being scrutinized for using their discretion in ways that might not be socially desirable or fairly applied, which may lead them to become more legalistic. In turn, this may lead officers to increase their use of formal responses, including arrests, citations, and written reports. One might argue that similar forces on discretion could inhibit citizens from calling the police, reporting crimes, or acting as witnesses for others, as it may increase their risk of retaliation, involvement, or victimization.

A related but different conceptualization of how BWCs may modify behavior has been examined by Wallace, White, Gaub, and Todak (2018), and White, Gaub, and Todak (2018). Particularly after the Ferguson, Missouri police officer shooting of Michael Brown, the idea of “de‐policing” or the “Ferguson Effect” was raised by police leaders^5^ and studied by scholars (see, e.g., Maguire, Nix, & Campbell, 2017; Marier & Fridell, 2020; Nix & Wolfe, 2016; Pyrooz, Decker, Wolfe, & Shjarback, 2016; Rosenfeld, 2015; Shjarback, Pyrooz, Wolfe, & Decker, 2017). De‐policing is hypothesized to occur when officers reduce their proactive activities because it could increase their risk of being recorded and scrutinized for their actions. This reaction might seem most likely for heavy‐handed and controversial proactive activities such as excessive or unconstitutional stop‐question‐and‐frisks. The notion of de‐policing, however, could extend to any “extra” policing beyond responding to 911 calls (e.g., community engagement, proactive/directed patrols at crime hot spots, traffic stops, and problem‐oriented policing activities). In turn, because proactive policing is believed to help prevent or deter crime (see National Academies of Sciences, 2018), researchers have also examined whether de‐policing would result in increases in crime (Rosenfeld, 2015, examined this phenomenon in St. Louis). Wallace et al. (2018) combined ideas of self‐awareness and deterrence with organizational theory related to discretion, motivation, and environment to hypothesize about whether BWCs might cause officers to reduce their self‐initiated activity (they did not find such an effect).

### 3.3 Prior reviews

The first review of BWC research was conducted by White (2014), who discovered that only five studies had been undertaken as of September 2013 (Farrar, 2012; Goodall, 2007; Katz, Choate, Ready, & Nuňo, 2015 6; Mesa Police Department, 2013; ODS Consulting, 2011). This meant that almost a third of U.S. agencies had already adopted BWCs, and widespread adoption was already occurring in the U.K., despite the lacuna of knowledge about their effectiveness. Fortunately, researchers have become very interested in studying BWCs in the latter half of the 2010s. For example, by November 2015, Lum et al.'s (2015) review of both completed and in‐progress studies for the Laura and John Arnold Foundation found that completed studies about BWCs had grown to more than a dozen, with 30+ additional studies underway. Later, Cubitt, Lesic, Myers, and Corry (2017) reviewed 11 articles on the impacts of BWCs. Although they concluded the overall methodological state of research was weak, they were optimistic about BWCs providing “an effective law enforcement option” (p. 392), in that BWCs could reduce crime rates, reduce complaints against officers, and more effectively document evidence. Similarly, Maskaly et al. (2017), in a review of police and citizen outcomes, found 21 empirical studies as of January 2017, which led them to conclude that police were receptive to BWCs, and that the cameras can exert positive effects on police behavior.

In their comprehensive narrative review of BWCs, Lum et al. (2019) discovered approximately 70 published or publicly available studies of BWCs that contained over 110 sub‐studies examining various outcomes and aspects of BWCs as of June 2018. Lum et al.'s review was not a meta‐analysis and did not synthesize effects across studies. They also looked at a wider range of studies, subjects, methodologies, and outcomes to examine the state of research on BWCs. In particular, they grouped studies into six topical categories: (a) the impact of BWCs on officer behavior; (b) officer attitudes about BWCs; (c) the impact of BWCs on citizen behavior; (d) citizen and community attitudes about BWCs; (e) the impact of BWCs on criminal investigations; and (f) the impact of BWCs on law enforcement organizations.

Lum et al. (2019) concluded that although it seemed that many agencies, officers, and citizens support BWCs, cameras had not consistently had the effects anticipated (or feared) by either police officers or citizens. They argued that anticipated effects may have been “overestimated” (p. 110) and that behavioral changes in the field may be “modest and mixed” (p. 111). Lum et al. (2019) also observed that while several studies suggested that BWCs could reduce citizen complaints against police, it remained unclear *why* the decline occurs. Their findings on police use of force, another prominent outcome in BWC research, were equivocal given that studies did not seem to show that BWCs had consistent effects on officer behaviors. Further, they pointed out some outcomes that needed more research—in particular, the impact of BWCs on police–citizen relationships, accountability systems, and racial and ethnic disparities in policing outcomes. At the same time, Lum et al. stated that BWCs would continue to be adopted by police agencies, which makes the production and synthesis of rigorous research even more essential to this policy area.

In their “review of reviews” commentary, Gaub and White (2020) characterize Lum et al.'s assessment as “gloomy” (p. 13). They suggest that other reviews, including their own assessment from their collection of outcomes for the U.S. Department of Justice BWC Policy and Implementation Program (see White, Gaub, & Padilla, 2019a, 2019b), are more optimistic about the future of BWCs (see also Malm, 2019). These disagreements about the state of knowledge on BWCs, and the fact that a great deal of investment has already been made in them, require more clarity in this research area so that police agencies can make the most informed decisions given the research available. As Lum et al. extensively describe (see also discussions by Braga et al., 2019; White, 2019), BWC research seems to be marked by heterogeneous findings, which suggests that outcomes may be influenced by various contextual and methodological factors. Findings might be moderated by the quality of research studies or the manner in which cameras are implemented and used across sites. As Braga et al. (2019) aptly state, “a comprehensive and systematic review of these kinds of moderators across studies that might explain the observed heterogeneity in study findings seems warranted” (p. 20). This meta‐analysis addresses these issues.

## 4 OBJECTIVES FOR THIS REVIEW

Given the widespread diffusion of BWCs in policing, the enormous costs related to this adoption, and the expectations about BWCs’ potential effects—both positive and negative—on police and citizen behaviors, the first objective of this review is to synthesize high‐quality research evidence on the impacts of BWCs on several outcomes of interest to police, policymakers, and the wider community. This review will focus on examining two categories of effects of BWCs:

- The impacts of BWCs on officer behaviors, as measured by complaints against officers; officer use of force; arrest and citation behavior; officer‐initiated activities (e.g., general self‐reported activity, traffic stops, and pedestrian stops/field interviews/stop‐question‐and‐frisks); incident report writing; and other measures of officer behaviors. We note that many of these measures might also reflect the impact of BWCs on citizen behaviors, as discussed above.
- The impacts of BWCs on civilian behaviors, as measured by community members’ compliance with police commands (as measured by resisting arrest or assaults against officers) and their calls for police service.

Additionally, we reiterate a point from the initial protocol for this meta‐analysis (see Lum, Koper, Wilson, et al., 2019): this review does not examine the impact of BWCs on case investigations, court processes, or court dispositions from investigations of crime.^7^ It is important to note that police and prosecutors have placed a growing emphasis on the use of BWCs to collect evidence and secure the prosecution and conviction of criminal offenders (Merola, Lum, Koper, & Scherer, 2016). These uses focus on a different set of outcomes and objectives (i.e., the prosecution of people, not the police) than those initially envisioned by citizens and municipalities who pushed for police use of BWCs. Thus, specific findings on the impacts of BWCs on criminal investigations, detections, guilty pleas, and convictions (see, e.g., Ellis et al., 2015; Goodall, 2007; Morrow, Katz, & Choate, 2016; ODS Consulting, 2011; Owens, Mann, & Mckenna, 2014; Yokum, Ravishankar, & Coppock, 2019) are not included in either the initial protocol or this review (although other outcomes from these studies may be included). We believe those findings deserve separate discussion from the impact of BWCs on officer and citizen behaviors and encourage others to take up this analysis.

The second objective of this review is to explore possible explanations for the heterogeneous effects of BWCs on officer and citizen behaviors found across studies. As White (2019), Malm (2019), and Gaub and White (2020) argue, evaluations of BWCs tend to be carried out in single agencies which differ in terms of their organizational characteristics, and environmental, community, and political contexts. Additionally, agencies differ in the way they implement BWCs, which may also influence the impact of BWCs on officer behaviors. For example, Ariel et al. (2016a) found that differences in the levels of officer discretion in turning on and off cameras may lead to different outcomes in use of force outcomes across agencies. Outcome differences may also result from whether studied officers were mandated or volunteered to wear cameras (see discussions by Katz, Huff, Webb, & Johnson, 2019). Methodological or research‐related differences in studies may also contribute to heterogeneous findings, which are commonly analyzed in systematic reviews. These include measures of internal validity, notably randomization, contamination, and fidelity differences across studies. Given these concerns, we proposed in the protocol to carry out a number of post‐hoc moderator analyses based on the availability of information found in eligible studies. We detail both the moderator and sensitivity analyses in the methodology section below.

Overall, the goal of this review is to provide practical information about the impacts of BWCs on a range of important outcomes to citizens, police agencies, municipalities, governments, oversight groups, and nongovernmental organizations. Knowledge from this review is intended to provide the police with more information as they consider whether to adopt BWCs or to more carefully consider their uses and expectations if agencies have already adopted them. Because technologies often lead to unintended consequences for both agencies and the communities they serve (Koper, Lum, & Willis, 2014), research syntheses can also help to highlight these consequences and help agencies and communities plan for (or temper their expectations of) future impacts of BWCs. This review will hopefully continue to facilitate the debate and conversation about incongruent expectations of BWCs between officers and civilians and provide a more holistic view of cameras for municipalities and governments, who are ultimately funding them.

## 5 METHODOLOGY

### 5.1 Criteria for including and excluding studies

#### 5.1.1 Types of study designs

Both experimental and quasi‐experimental designs were included in this review. Experimental designs were eligible if the treatment was randomly assigned to the units of analysis. Quasi‐experimental studies with nonrandom assignment were eligible for inclusion if a similar comparison group was evident in the study. Study authors could develop a comparable comparison group using propensity scores or other matching techniques achieved through the use of statistical controls. Matching may be at the individual or group level, and statistical control methods could include regression, analysis‐of‐covariance, and propensity score matching, among others. The use of a statistical control method is sufficient for inclusion; we do not exclude studies based on a subjective assessment of the quality of the statistical controls. Instead, any quasi‐experimental design that controls for possible explanations for BWC outcomes, such as officer, civilian, or event characteristics, was eligible. Quasi‐experimental designs that do not have a comparison group or do not use the above methods to achieve comparability were not eligible for inclusion in this review.

One exception to this rule that was not mentioned in the initial protocol is that we treat noncomparison group interrupted time series studies as quasi‐experiments if they had adequate data for modeling time trends, seasonal patterns, and autocorrelation as means of creating a control condition counterfactual (see Box & Tiao, 1975; McCleary & Hay, 1980). Such studies had to have at least two years and 24 data points for both the preintervention and post‐intervention periods.

#### 5.1.2 Types of participants

The populations of interest for this review are law enforcement officers and civilians. We note that because BWC studies employ various units of analysis, we include officers, groups of officers, shifts, non‐law enforcement personnel (e.g., community members and citizens), or geographic areas, as study units. We excluded studies of BWC use by those who work in court settings, corrections, or private security. We made this decision given that BWCs are primarily used by uniformed police officers, and almost all of the BWC research focuses on police officer use of BWCs. One study that we initially included but subsequently excluded after peer review was Ariel et al.'s (2019) United Kingdom rail stations study. This study examined the impact of BWCs on rail station gate agents who are not law enforcement officers. For this reason, two external reviewers believed this study was not eligible, to which we agree.

#### 5.1.3 Types of interventions

The intervention examined in this review is the wearing of the BWC by a law enforcement officer.

#### 5.1.4 Types of outcome measures

Only outcomes and effects from studies that attempt to measure officer or citizen behavior, not their attitudes or perceptions, were examined for this review:

Measures of officer behavior

- Complaints against officers
- Use of force
- Arrests
- General levels of self‐initiated activities of officers as measured by officer‐initiated calls for service
- Stop and frisk or field interrogation stops
- Traffic stops or tickets
- Incident reports written
- Response time
- Time on scene
- Ordinance citations (not traffic‐related)

Measures of civilian behavior

- Dispatched calls for service
- Assaults on officers/officer injuries
- Resistance against officers

#### 5.1.5 Specific deviations from the protocol

The above list differs from that initially presented in the protocol (see Lum, Koper, Wilson, et al., 2019, section 3.1.4) in two ways. First, the list is more specific and extended. For example, we expanded the general “proactive activities” category from the protocol to include general levels of self‐initiated activities of officers as measured by officer‐initiated calls for service; stop and frisk or field interrogation stops; and traffic stops or tickets, as we found that these types of proactive activities have been commonly analyzed (specifically and separately) in BWC studies. We also added reported incidents, response time, time on scene, and ordinance citations given that our literature review uncovered research examining these outcomes. We also replaced “criminal or disorderly conduct” with “dispatched calls for service,” given that this is how this construct (which reflects citizen behaviors) is commonly measured in the literature.

Second, in the initial protocol for this review (see Lum, Koper, Wilson, et al., 2019), we included citizens’ “willingness to call the police or cooperate in criminal investigations.” This measure was removed from this review for two reasons. First, we captured willingness to call the police with actual dispatched calls for service, which is a more commonly studied measure. Second, we removed this construct because it focused more on perceptions and feelings of willingness, not behavior.

#### 5.1.6 Duration of follow‐up

The expected effects of BWCs are immediate, and they are presumed to have an effect while they are being used. As such, the outcomes in BWC research are usually measured concurrently with the intervention. We did not find any studies that measured effects at a follow‐up period when BWCs were no longer in use. For example, two studies (Koslicki, Makin, & Willits, 2019; Sutherland, Ariel, Farrar, & De Anda, 2017) both measured the long‐term effects of BWCs three years after implementation, but in both studies, BWCs were still being used by those agencies.

### 5.2 Search strategy and screening process

The initial search was contracted out to the GPD^8^ team at the University of Queensland (Elizabeth Eggins and Lorraine Mazerolle) and Queensland University of Technology (Angela Higginson). The results of their search, which included studies through December 31, 2018, were provided to the GMU team in June 2019. Due to the fast‐moving nature of this research area, however, the George Mason University (GMU) team conducted a supplemental search to identify additional studies completed from January 1 to September 30, 2019. The full search process is now detailed.

According to Higginson, Eggins, Mazerolle, & Stanko, (2015, p. 1), the GPD “is a web‐based and searchable database designed to capture all published and unpublished experimental and quasi‐experimental evaluations of policing interventions conducted since 1950. There are no restrictions on the type of policing technique, type of outcome measure or language of the research.” The GPD is compiled using systematic search and screening techniques, which are reported in Higginson et al. (2015) and detailed in the Supporting Information Appendices A and B. Broadly, the GPD search protocol includes an extensive range of search locations to ensure that both published and unpublished experimental and quasi‐experimental studies in policing are captured across criminology and allied disciplines and that are aligned with Campbell search strategies and processes. Only a portion of the GPD is publicly available; full searches can only be conducted internally by the GPD team.

To capture studies for this review, the GPD research team used BWC‐specific terms to search the GPD corpus of full‐text documents that have been screened as reporting a quantitative impact evaluation of a policing intervention. Specifically, the team used the search parameter “camera* video* OR BWC* OR BWV*” to search the title and abstract fields of the corpus of documents published between January 2004^9^ and December 31, 2018. December 2018 was used as the cutoff because the GPD team began the search at the beginning of 2019.

The results were compiled and provided to the GMU team in June 2019. The GPD search team also had updated and processed GPD records for a range of additional gray literature sources received from their library in later 2019. They then conducted a supplemental search of the GPD database (again, only through December 31, 2018, to match their initial search), and provided the results of that additional search of the GPD to the GMU team in December 2019.

Because research on BWCs is a fast‐moving and continually growing area, the GMU research team carried out additional searches, after receiving the GPD's results, while they were completing their review (between July 2019 and January 2020) to ensure that this review included the latest BWC research through September 2019. This search included the following steps: Upon receiving the GPD's search, the team cross‐referenced every study found in Lum et al. (2019) to the findings of the GPD to capture any eligible studies which may have been missing from the GPD search. Next, they examined the Body‐Worn Camera Toolkit,^10^ which contains outcome directories developed by White et al. (2019a, 2019b) on BWC research. The research team also carried out an additional search—using the exact same parameters as the GPD team—for documents published between January 1 and June 30, 2019, using Google Scholar and the EBSCO Criminal Justice Abstracts database. Between July 2019 and January 2020, additional studies were also presented to the GMU team through a variety of alerts, resources, and correspondences from researchers who had written new reports that had yet to be published. Finally, to ensure that no new, unpublished studies were overlooked, in January 2020, the GMU team contacted 82 individuals (listed in the Supporting Information Appendix C) who had published evaluation or nonevaluation research on BWCs and provided them with a list of all eligible studies under consideration as well as the search criteria that were used to identify studies. These researchers were asked to identify any additional eligible studies that had been completed as of September 2019.

To ensure a systematically recorded database system was used across multiple coders, a data extraction and collection database was created using LibreOffice,^11^ a freely available office suite, combined with Amazon's cloud services. All titles and abstracts of research articles or reports discovered from these various search efforts described were entered into this system, which was then used to select and code eligible studies. The GMU team used a two‐coder system for every coding process for this systematic review, from the examination of abstracts and full text for eligible studies to coding characteristics of studies, outcomes, and effect sizes for each eligible study. Each two‐coder abstract‐review team consisted of one principal investigator (Lum or Koper) and one doctoral student (Goodier or Stoltz). Pairs were assigned to each abstract, and principal investigator‐student dyads were equally mixed across the abstracts. Each pair coded each abstract provided from the GPD and supplemental searches as “potentially eligible,” “not eligible,” “relevant review” (to flag that documents that could be useful but that are not eligible as a study), and “unclear” using our initial criteria. If there were disagreements in coding, the other principal investigator would act as a third‐party judge. Studies with differences that persisted or could not be mitigated (e.g., if one coder continued to believe a study was “potentially eligible”) were retained, and the full text of the study was examined in the next screening process.

Once studies were determined by at least one coder to be “potentially eligible,” the full‐text document of each study was obtained, labeled, and assigned to a principal investigator‐student dyad as described above. After reviewing the full text of an article or report, each coder then coded “yes,” “no,” or “uncertain” for each of the above criteria described in 5.1 above. If a coder answered “yes” to all of the criteria, the study was coded as “eligible.” If not, the study was coded as “not eligible” or “unclear.” If there were disagreements in coding, the other principal investigator would act as a third‐party judge. The GMU team would also meet on a regular basis to discuss this process and the coding of specific studies; if a study continued to draw debate, an additional expert and study author (Wilson) was consulted to determine the eligibility of a document for inclusion.

### 5.3 Criteria for determination of independent findings

The unit‐of‐analysis for this review is the research study, which is defined as a distinct sample of study participants involved in a common research project. Multiple reports (e.g., publications, technical reports) from a common research study are coded together as a single study. A research study was treated as unique only if the study sample did not include study participants included in any other coded study. Multiple effect sizes were coded from studies with multiple outcomes. Statistical independence was maintained in all statistical analyses. In these studies, each outcome construct was typically measured by only one dependent variable. For two studies, a single outcome construct was measured by two dependent variables. The average of the two effect sizes within each study and construct was used in the meta‐analyses, thus ensuring that each study (or sub‐study) contributed no more than one effect size to each meta‐analysis.

### 5.4 Details of study coding categories

Per Campbell's conventions, all studies were double‐coded. Details of all data collected at the study‐level, outcome‐level, and effect‐size level are provided in the Supporting Information Appendix D. Coding included identification information for each study; descriptive features of studies (including information on treatment and control conditions, locations and organizations involved, dates of study, and BWC implementation); information on the nature of the BWC policies and use; method and design features of studies; risk‐of‐bias indicators as modified from Cochrane tools; outcomes selected and units measured; and effect size coding. The full coding of each study and each effect is available publicly at the Open Science Framework (OSF) depository for this study.^12^

We prioritized more general measures of a construct over less general measures since these appeared most regularly across the studies. For example, there are many different types and categories of use of force (e.g., hands only, nonlethal instruments, and firearm use) and complaints (i.e., complaints of rudeness and service delivery). For this review, we selected the most general measure of use of force or complaints provided (i.e., counts of reports of use of force or complaints generated). Additionally, there are many different types of crimes and infractions that may receive arrest and citations, but only the most general measure of arrest and citation was measured (i.e., “all arrests” rather than “arrest for violence” and “arrest for property crimes”). Similarly, for non‐police civilian behaviors, the more general behavioral categories were measured (i.e., “resisting arrest” or “assault on officers”) rather than specific types of assaults or resistance. We also made the decision to collect three separate measures of officer proactivity. These include all self‐initiated calls for service, as well as field interviews/stop‐and‐frisks and traffic stops and tickets more specifically. In some studies, self‐initiated calls include stop and frisks, field interrogations, and traffic citations, but this was not always clear in each study, nor always the case. Thus, for this particular outcome category, three separate constructs were retained.

### 5.5 Statistical procedures and conventions

Based on prior work by Lum et al. (2019), we expected to find a sufficient number of studies to conduct a meta‐analysis for the outcomes described above. The initial protocol specified that various effect sizes were to be converted to Cohen's *d* except for outcomes that are more naturally measured dichotomously, in which case the odds ratio would be used. Upon coding and analyzing the studies, however, it became apparent that neither the odds ratio nor Cohen's *d* was appropriate for this review. In almost all cases, the underlying data from eligible studies were based on counts. In a few cases, the counts were dichotomized, in that the study authors converted the count of incidents per shift or officer to a dichotomous choice of whether an incident did or did not occur for that shift or officer.

The problem with using Cohen's *d* for this meta‐analysis is that it is scaled differently across different studies making the effect sizes noncomparable and unusable for our purpose (i.e., they should not be combined via meta‐analysis). The logic of Cohen's *d* is to standardize a mean difference between two groups relative to the standard deviation on that outcome. Ideally, this would be the population standard deviation for these data, although we almost universally use the sample standard deviation as an estimate of this quantity. In the prototypical case, we standardize on the variability across individuals. If the unit‐of‐analysis changes to shifts instead of individual officers, then the standardization also changes (this would be like changing the scaling of temperature from Fahrenheit to Celsius). That is, a Cohen's *d* based on the variability in the use of force across shifts and a Cohen's *d* based on variability in the use of force across the individual officers from those shifts will differ, with the former being larger, even though the underlying treatment effect remains the same. For Cohen's *d*s to be comparable, they must be based on a common unit‐of‐analysis. As discussed below, having a stable unit‐of‐analysis is problematic for count data.

Count data are dispersed over time and space and can be divided by time and space arbitrarily (or by some other unit). This division converts the count into a rate (i.e., the rate per year, per month, per officer, per jurisdiction). We can divide a count by increasingly smaller units, such as months, weeks, days, hours, or by 100,000 in the population, or 1,000,000 in the population. As we divide the count by smaller units of time/space/population, our sample size increases, and the rate decreases (rate per month is less than the rate per year), as does the standard deviation of the rates. These changes affect the value of Cohen's *d*. It is easy to simulate this, and the change can be by orders of magnitude (e.g., on some simulated data, Cohen's *d* changed from −0.23, to −0.54, to −1.45, when we changed the rate from days to weeks to months, respectively). The incidence rate ratio, the effect size used in this review, remains unchanged across different divisions of space, time, or population. For these reasons, Cohen's *d* is not suitable for these data.

We drew from Poisson‐based regression models (including quasi‐Poisson and negative binomial) to develop appropriate effect sizes based on incident rates. For post‐test only data, the effect size was the logged incident rate ratio (the log of the ratio of the incident rate for the BWC condition to the non‐BWC condition). For pre–post by BWC/non‐BWC data, the effect size was the logged RIRR. These are analogous to a simple mean difference and difference‐in‐differences effect estimates, just on the log scale. The formulas and detailed methods are reported in the Supporting Information Appendix E and Supporting Information Appendix F includes the script file (R Code) for all analyses. All results were converted from the logged incident rate ratios into a percent change for ease of interpretation.

Although the specific formula used varied depending on the nature of the data provided, the formula that best defines the logged RIRR is as follows:

$$\;\log\text{ RIRR}=\log\left(\frac{{\bar{x}}_{T_{2}}{\bar{x}}_{C_{1}}}{{\bar{x}}_{T_{1}}{\bar{x}}_{C_{2}}}\right),$$

where each mean is the sum of the counts divided by a standardizing unit such as the length of time, number or size of a geographic area (e.g., number of jurisdictions), or the number of persons (e.g., officers). Essentially, each mean is a rate or the number of counts per some unit. The subscripts indicate the treatment (*T*) or control (*C*) and baseline (1) or intervention (2) periods. Conceptually, this is comparing the proportion change in the rate for the treatment condition relative to the proportion change in the rate for the control condition. When the number of units are equal, the total counts rather than means (rates) can be used.

Meta‐analysis was conducted using random‐effects models estimated via restricted maximum likelihood, using the **metafor** package (Viechtbauer, 2010) in the **R** statistical application (R Core Team, 2013). In the protocol, we did not specify *a priori* the moderator or sensitivity analyses that would be conducted, although we provided some examples of ad hoc analyses that would be run given the data that could be collected. However, we anticipated, as discussed above, that moderator analysis could be conducted on types of research designs, locations of studies, differences in BWC policies, and differences in BWC implementation within studies. Moderator analyses of a single categorical variable were fit using the analog‐to‐the‐ANOVA method, also under a random‐effects model. In **metafor**, this is done by first estimating a meta‐regression model and then using the predict function to generate the mean effect size and related statistics for each category of the moderator variable.

Publication selection bias was assessed in four ways. First, we compared the results from published and unpublished reports. Published documents include peer‐reviewed journal articles, books, and book chapters. All other report forms, such as theses, technical reports, government, and agency reports, were considered unpublished. Second, we performed a trim‐and‐fill analysis on the major outcome categories. Third, we visually inspect a funnel plot on the major outcome categories. Fourth, we performed Egger's test of publication selection bias.

We did not, as per the protocol, include qualitative research in this systematic review, except as to provide context for interpreting results. We point to the Lum et al. (2019) review, which examined a large amount of qualitative and survey research, which provides additional context to this meta‐analysis.

## 6 RESULTS

### 6.1 Results of the search

Table 1 provides the results of the search process described above. In total, the search yielded 558 possible abstracts. Of these, nine were duplicate records and were removed, yielding a final total of 549 abstracts discovered.

**Table 1:** Documents and abstracts discovered during search processes

Source	Abstracts
GPD initial searches (provided June 2019 and December 2019)	516
Supplemental cross‐reference from Lum et al. (2019)	10
Supplemental cross‐reference from White et al. (2019a, 2019b) BWC toolkit	3
Supplemental January–June 2019 search	21
Other additions discovered or sent to us during the review	8
Minus true duplicates	−9
Total abstracts/titles discovered	549

The abstract screening process resulted in 51 potentially eligible abstracts from these 549 records to be further examined using full‐text review. The full‐text review then yielded 35 of the 51 documents as eligible for analysis (some reporting on different parts of the same study). The reasons for ineligibility aligned with our selection criteria. For instance, eight studies did not meet the methodological requirements as defined above for the specific outcomes of interest. For difficult decisions, we conferred with study first authors directly (e.g., Goodison & Wilson, 2017; White et al., 2018) to ensure we were interpreting their research correctly. Five studies did not focus on outcomes of interest (these studies often highlighted perceptions rather than behaviors). An example of this type of study is Demir, Apel, Braga, Brunson, and Ariel (2020).^13^ Further, we initially included one study that was removed after peer review because it was not focused on law enforcement. As noted, this was the U.K. Rail Station study conducted by Ariel et al. (2019), which examined BWC use by rail station staff. Finally, we excluded two additional studies because the outcome of interest was not reported with sufficient information to calculate an effect size.

After careful inspection, 30 independent studies were identified from the 35 documents. The labels used for these 30 studies are provided in Table 2, along with their associated documents.

**Table 2:** Eligible studies (*N *= 30) and associated documents for each study

Label used for each study (alphabetized by location of the study)	Associated documents
Braga et al. (2019) BOSTON, MA	Braga, Barao, McDevitt, and Zimmerman (2018); Braga, Barao, Zimmerman, Douglas, and Sheppard (2019)
Ariel (2016, 2017) DENVER, CO	Ariel (2016a); Ariel (2016b)
Bennett et al. (2019) FAIRFAX COUNTY, VA	Bennett, Bartholomew, and Champagne (2019)
Headley et al. (2017) HALLANDALE BEACH, FL	Headley, Guerette, and Shariati (2017)
Sousa, Braga et al. (2015, 2018) LAS VEGAS, NV	Sousa, Coldren, Rodriguez, and Braga (2016); Braga, Sousa, Coldren, and Rodriguez (2018)
Grossmith, Owens, Finn, et al. (2015, 2018) LONDON, UK	Grossmith, Owens, Finn, Mann, Davies, and Baika (2015); Owens and Finn (2018)
Mesa PD, Ready and Young (2013, 2015) MESA, AZ	Mesa Police Department (2013); Ready and Young (2015)
Stolzenberg et al. (2019) MIAMI‐DADE, FL	Stolzenberg, D'Alessio, and Flexon (2019)
Peterson, Lawrence, et al. (2018, 2019) MILWAUKEE, WI	Peterson, Yu, La Vigne, and Lawrence (2018); Lawrence and Peterson (2019)
Koslicki et al. (2019) NORTHWEST CITY	Koslicki, Makin, and Willits (2019)
Jennings et al. (2015) ORLANDO, FL	Jennings, Lynch, and Fridell (2015)
Katz et al. (2015, 2016) PHOENIX, AZ (Maryvale)	Katz, Choate, Ready, and Nuňo (2015); Morrow, Katz, and Choate (2016); Hedberg, Katz, and Choate (2016)
Katz et al. (2019) PHOENIX, AZ (not Maryvale/Mandated)	Katz, Huff, Webb, and Johnson (2019)
Katz et al. (2019) PHOENIX, AZ (not Maryvale/Volunteer)	Katz, Huff, Webb, and Johnson (2019)
Ariel, Farrar, et al. (2012, 2013, 2015, 2017) RIALTO, CA	Ariel, Farrar, and Sutherland (2015); Farrar (2012); Farrar and Ariel (2013); Sutherland, Ariel, Farrar, and De Anda (2017)
White et al. (2018) SPOKANE, WA	White, Gaub, and Todak (2018); Wallace, White, Gaub, and Todak (2018)
Wallace et al. (2018) SPOKANE, WA	
Jennings et al. (2017) TAMPA, FL	Jennings, Fridell, Lynch, Jetelina, and Reingle Gonzalez (2017)
Mitchell et al. (2018) URUGUAY	Mitchell, Ariel, Emilia Firpo, Fraiman, Del Castillo, Hyatt, Weinborn, and Brants Sabo (2018)
Yokum et al. (2019) WASHINGTON, DC	Yokum, Ravishankar, and Coppock (2019)
Henstock and Ariel (2017) WEST MIDLANDS	Henstock and Ariel (2017)
Ariel et al. (2016, 2017, 2018) SITES A, B, C, D, E, F, H, I, J, K (ten separate studies)	Ariel, Sutherland, Henstock, Young, Drover, Sykes, Megicks, and Henderseon (2016a); Ariel, Sutherland, Henstock, et al. (2016b); Ariel, Sutherland, Henstock, et al. (2017); Ariel, Sutherland, Henstock, et al. (2018) (same authors)

Table 2 requires three points of elaboration. The first involves the three Phoenix, Arizona, studies conducted by Katz and colleagues. One study, labeled “Katz et al. (2015, 2016) PHOENIX, AZ (Maryvale),” was the authors’ early pilot study in Maryvale, a small sub‐section of Phoenix. The second and third studies, labeled “Katz et al. (2019) PHOENIX, AZ (not Maryvale/Mandated)” and “Katz et al. (2019) PHOENIX, AZ (not Maryvale/Volunteer)” was a later study conducted on the rest of Phoenix, not including the Maryvale area. We treated this later non‐Maryvale study as two separate studies. In the “volunteer” study, a group of officers selected randomly from a larger pool of eligible officers was given the option of wearing BWCs. Those who volunteered to wear the BWCs were compared to a randomly determined group of control officers from the same eligible pool.^14^ We treated this comparison as a quasi‐experiment. In the “mandated” study, this same control group was compared to another randomly selected group of officers from the eligible pool who were required to wear BWCs. We treated this second set of comparisons as a separate RCT. Because different outcome constructs were reported for these two treatment groups, no issue of statistical dependency arose at the analysis stage, as only one of these was included in any given analysis.

The second issue regarding Table 2 refers to the Spokane, Washington study, labeled “White et al. (2018) SPOKANE, WA” and also “Wallace et al. (2018) SPOKANE, WA.” These two articles present unique findings but are both based on the same experiment conducted in Spokane, Washington, on the same group of officers. However, White et al. (2018) examined selected outcomes measured at the officer level, whereas Wallace et al. (2018) examined a different set of outcomes measured at the incident level. Thus, while we count the Spokane study as a single study, effects from the two articles will be labeled separately to signal that the associated outcomes and effect sizes arise from two different measures, methods, and documents that are not easily combined.

The third issue focuses on four related documents (Ariel et al., 2016a, 2016b, 2017, 2018) and is labeled “Ariel et al. (2016, 2017, 2018) SITES A, B, C, D, E, F, H, I, J, or K.” Across these four documents, Ariel and colleagues present the results of 10 studies of jurisdictions that are kept anonymous.^15^ Ariel and colleagues combined these studies in these documents for their analyses, but we treat them as separate studies after conversations with the study authors. We note that several essential study and implementation elements are not reported for these sites. Given the missing information from these studies, we remove them in the sensitivity analyses to determine if their inclusion affects our findings.

### 6.2 Description and characteristics of the studies

Evaluations of the impacts of technology in policing are unusual, even for technologies that rapidly diffuse into the profession. A good example is license plate‐reader technology, which also experienced a rapid diffusion in the early 2000s but has only been evaluated for its crime prevention potential in a handful of studies (see discussion in Lum, Koper, Willis, et al., 2019). This has not been the case with BWCs. Although few studies existed at the time BWCs began their rapid adoption and diffusion (e.g., Goodall, 2007; ODS Consulting, 2011), BWCs have since been studied extensively. The earliest documented randomized controlled experimental trial of the impact of BWCs on officer or citizen behavior was likely Farrar's master's thesis for the University of Cambridge, which reported on the Rialto experiment, completed in 2012 (reported as a peer‐reviewed publication by Ariel et al., 2015). Around the same time, the Mesa, Arizona Police Department (2013) reported its quasi‐experiment. Over a period of only 6–7 years, there have been at least 30 outcome evaluations examining the impact of BWCs on behaviors. The magnitude of this corpus of research is notable, and this group of studies only represents a subset (albeit a large portion) of all BWC empirical research.

Due to space limitations, Table 3 displays only some characteristics of each of the 30 eligible BWC studies included in this review. However, we provide the full data for all data elements collected for each study (as described in the Supporting Information Appendix D) at the Open Science Framework (OSF) depository for this study.^16^ For each study, Table 3 displays the shortened label used from Table 2; selected information about the jurisdictions; the types of research design and unit of analysis used; the number of officers involved; the intervention start and end dates; and the funding source. All of the interventions of BWC use within the parameters of this systematic review occurred within a short time frame (2011–2018) and results were reported quickly (2012–2019).

**Table 3:** Select study‐level descriptive information

Study name	Population	Year BWC implemented	Research design	Unit‐of‐assignment	No. officers in study	Intervention start date	Intervention end date	Funding source
Ariel (2016, 2017) DENVER, CO	633,777	2014	QE: statistical adj. for baseline	Police‐defined geographic areas	632	Jul 2013	Dec 2014	No funding received
Ariel et al. (2016, 2017, 2018) SITE A	161,400		RCT: simple	Shift	546			No funding received
Ariel et al. (2016, 2017, 2018) SITE B	285,700		RCT: simple	Shift	23			No funding received
Ariel et al. (2016, 2017, 2018) SITE C	203,800		RCT: simple	Shift	111			No funding received
Ariel et al. (2016, 2017, 2018) SITE D	285,700		RCT: simple	Shift	22			No funding received
Ariel et al. (2016, 2017, 2018) SITE E	751,500		RCT: simple	Shift	870			No funding received
Ariel et al. (2016, 2017, 2018) SITE F	188,400		RCT: simple	Shift	120			No funding received
Ariel et al. (2016, 2017, 2018) SITE H	108,817		RCT: simple	Shift	115			No funding received
Ariel et al. (2016, 2017, 2018) SITE I	26,757		RCT: simple	Shift	60			No funding received
Ariel et al. (2016, 2017, 2018) SITE J	151,533		RCT: simple	Shift	150			No funding received
Ariel et al. (2016, 2017, 2018) SITE K	249,470		RCT: simple	Shift	105			No funding received
Ariel, Farrar, et al. (2012, 2013, 2015, 2017) RIALTO, CA	100,009	2012	RCT: simple	Shift	54	Feb 2012	Feb 2013	Rialto Police Department and Jerry Lee Centre of Experimental Criminology
Bennett et al. (2019) FAIRFAX COUNTY, VA	1,142,004	2018	QE: time series	Other	259	Mar 2017	Dec 2018	American University and the Charles Koch Foundation
Braga et al. (2019) BOSTON, MA	658,279	2016	RCT: block randomized	Officer	281	Sep 2015	Aug 2017	City of Boston and Rappaport Institute for Greater Boston
Grossmith, Owens, Finn, et al. (2015, 2018) LONDON, UK	8,173,941	2008	RCT: cluster randomized	Enforcement group	2,060	May 2014	Apr 2015	Metropolitan Police Service, College of Policing, and Mayor's Office for Policing and Crime
Headley et al. (2017) HALLANDALE BEACH, FL	38,725	2015	QE: other	Officer	51	Jan 2015	Dec 2016	No funding received
Henstock and Ariel (2017) WEST MIDLANDS, UK	1,141,400	2014	RCT: block randomized	Shift	46	Jun 2014	Dec 2014	No funding received
Jennings et al. (2015) ORLANDO, FL	250,224	2014	RCT: block randomized	Officer	89	Mar 2013	Feb 2015	No funding received
Jennings et al. (2017) TAMPA, FL	355,603	2015	QE: propensity score	Officer	761	Mar 2014	Feb 2016	No funding received
Katz et al. (2015, 2016) PHOENIX, AZ (Maryvale)	237,352	2013	QE: other	Police‐defined geographic areas	110	Jan 2012	Jul 2014	Bureau of Justice Assistance, U.S. Department of Justice
Katz et al. (2019) PHOENIX, AZ (not Maryvale/Mandated)	1,393,820	2013	RCT: simple	Officer	297	Nov 2015	Nov 2018	Bureau of Justice Assistance, U.S. Department of Justice
Katz et al. (2019) PHOENIX, AZ (not Maryvale/Volunteer)	1,393,820	2013	QE: propensity score	Officer	310	Nov 2015	Nov 2018	Bureau of Justice Assistance, U.S. Department of Justice
Koslicki et al. (2019) NORTHWEST CITY	99,000	2013	QE: time series	Other	100	Jan 2009	May 2016	No funding received
Mesa PD, Ready and Young (2013, 2015) MESA, AZ	443,875	2012	QE: simple matching	Officer	100	Nov 2012	Oct 2013	Mesa Police Department, and Arizona State University
Mitchell et al. (2018) URUGUAY	3,369,299	2016	QE: other	Police‐defined geographic areas	208	Jan 2015	Mar 2017	No funding received
Peterson, Lawrence, et al. (2018, 2019) MILWAUKEE, WI	598,672	2015	RCT: block randomized	Officer	504	Jun 2015	Dec 2016	Bureau of Justice Assistance, U.S. Department of Justice
Sousa, Braga, et al. (2016, 2018) LAS VEGAS, NV	597,353	2011	RCT: simple	Officer	416	Mar 2011	Sep 2015	National Institute of Justice, U.S. Department of Justice
Stolzenberg et al. (2019) MIAMI‐DADE, FL	2,664,418	2016	QE: time series	Other	991	Jan 2005	Jun 2018	Bureau of Justice Assistance, U.S. Department of Justice
Wallace et al. (2018) SPOKANE, WA	210,695	2014	RCT: simple	Other	149	Jan 2013	Apr 2016	Laura and John Arnold Foundation
White et al. (2018) SPOKANE, WA	210,695	2014	RCT: simple	Officer	149	Nov 2014	Apr 2016	Laura and John Arnold Foundation
Yokum et al. (2019) WASHINGTON, DC	647,484	2015	RCT: block randomized	Officer	2,224	Nov 2014	Apr 2017	Laura and John Arnold Foundation

Abbreviations: QE, quasi‐experimental study; RCT, randomized controlled trial.

Table 4 provides summary statistics for each study. Whereas a majority of the studies examine BWC use in U.S. jurisdictions (57%), at least three have been conducted outside of the United States. There are likely more non‐U.S. studies since the 10 anonymous “global” studies (labeled “Ariel et al. (2015, 2017, 2018) SITE A, B, C, …”) also include studies from “around the world” (Ariel et al., 2016a, p. 752). Interestingly, many studies were not conducted on police agencies from highly populated cities; 47% of these studies examine jurisdictions of between 100,000 and 500,000 people, and three of the studies were conducted in locales with fewer than 100,000 people. Researchers from two universities dominate BWC studies: 47% of studies come from University of Cambridge‐affiliated individuals (always involving Ariel) and 17% coming from Arizona State University (mostly involving White, Katz, Wallace, and others). Twenty‐six of these studies have been published in peer‐reviewed journals (87%). Of these 26 studies, six also included unpublished materials (i.e., technical reports, thesis). Four studies were not published in a peer‐review journal (three technical reports, and one book).

**Table 4:** Key summary statistics of eligible body‐worn camera (BWC) studies (*N *= 30)

Characteristics	*N*	Percent
Country		
USA	17	56.7
Unknown	10	33.3
UK	2	6.7
Republic of Uruguay	1	3.3
Population size		
<100,000	3	10.0
100,000–500,000	14	46.7
500,001–1 million	6	20.0
>1 million	7	23.3
Research design		
Randomized controlled trial	20	66.7
Quasi‐experiment	10	33.3
Unit of analysis		
Shift	12	40.0
Officer	8	26.7
Geographic area	3	10.0
Time period	4	13.3
Incident and officer	2	6.7
Incident	1	3.3
Evaluation team		
University of Cambridge	14	46.7
Arizona State University	5	16.7
Florida International University	2	6.7
University of South Florida	2	6.7
Other teams (only 1 per team)	7	23.3
Publication type		
Journal	20	66.7
Journal/Tech report	5	16.7
Tech report	3	10.0
Book	1	3.3
Journal/Thesis/Other	1	3.3
BWCs use by the agency prior to the study		
Use of BWCs began very close to the time of the study or for the purposes of the study	14	46.7
BWCs were already in use by agency before the study began (selectively)	6	20.0
Unknown	10	33.3
Year BWCs were first implemented in the agency		
2012	2	6.7
2013	2	6.7
2014	5	16.7
2015	4	13.3
2016	4	13.3
2017	2	6.7
2018	1	3.3
Not reported	10	33.3
Nature of BWC use during the intervention		
Uniformed patrol only	25	83.3
Uniformed patrol and specialized units	4	13.3
Specialized units	1	3.3
BWC turned on by default		
Yes	25	83.3
No	1	3.3
Cannot tell	4	13.3
Discretion regarding on‐off		
Higher	9	30.0
Moderate	3	10.0
No or low	14	46.7
Cannot tell	4	13.3
Must inform citizens that BWC is on		
Yes	16	53.3
No	3	10.0
Not specified	11	36.7
In the 2 years prior to camera adoption, had the agency experienced a collaborative reform or sentinel event?		
No or not mentioned	25	83.3
Yes	5	16.7
Contamination of control condition		
Less likely	6	20.0
More likely	24	80.0
Fidelity of BWC implementation		
Higher	6	20.0
Lower	11	36.7
Unsure	13	43.3

BWC outcome evaluation research eligible for this review has been dominated by experimental studies. Two‐thirds of the studies used RCT designs, whereas a third employed a quasi‐experimental design of some type. Although only a guess, the large number of experiments reflected in this review may be the result of many studies being implemented at the same time BWCs were adopted by agencies. As Table 4 shows, 47% of the studies involved agencies that had implemented their BWCs around the same time (or for the purposes of) the study. An additional 20% of the studies were in agencies that were already using BWCs prior to the study (for the remaining third of the studies, this finding was unknown). For those that did report the year of the initial implementation of BWCs (regardless of when studies began), the implementation year was 2012 or later, and most often in 2014, 2015, or 2016. The average number of officers involved in these experiments and quasi‐experiments was 410 (minimum = 22; maximum = 2,224; median = 149).

All of the studies—with one exception—compared the same treatment condition (officers wearing BWCs) with the same control condition (officers not wearing BWCs). The exception was Mitchell et al.'s (2018) study in which the treatment condition involved officers wearing BWCs and saying a script versus a control condition in which officers did not wear BWCs and did not say a script.

BWC studies included in this review also varied in their units of analysis used for each study. Forty percent of the studies employed shifts as their unit of analysis, all of which were conducted by research teams that included Ariel and colleagues under the auspices of the University of Cambridge. These study authors compared officers wearing BWCs during randomly selected shifts (e.g., Monday day shift, Tuesday evening shift) to officers not wearing cameras during other shifts (e.g., Monday evening shift, Wednesday morning shift). In these designs, the same officers often carry out the treatment and control conditions depending on their shift assignment. Twenty‐seven percent of the studies examined treatment effects at the officer level, where cameras were randomly allocated to individual officers, and those wearing cameras were compared to those not wearing cameras. In three studies, all officers in a geographic area(s) were wearing BWCs and were compared with all officers in another area(s) who were not wearing BWCs. Four other studies used time periods as the unit of analysis. In the Spokane, Washington study, White et al. (2018) and Wallace et al. (2018) used two different units of analysis for the same study (officers and calls for service, respectively).

As White (2019) has asserted, understanding the context of BWC adoption may be necessary for explaining differences in the effects of BWCs. This information, however, was sometimes difficult to discern from studies.^17^ From what authors reported, at least 17% of the studies were carried out in locales that had, in the two years prior to their camera adoption, undergone some significant scrutiny in the form of collaborative reforms or consent decrees (two approaches to examining agency activities and accountability used by the U.S. Department of Justice) or some other form of an official review due to a significant sentinel event (such as an officer‐involved shooting). It is difficult to determine, however, the accuracy of this estimate, given that many studies did not discuss these issues. While we conduct moderator analysis on this study characteristic, conclusions should be interpreted cautiously.

Agencies examined in these studies also varied in terms of their official policies for BWC use. We collected information reported on agencies’ official policies regarding BWC use as well as their adherence to the implementation of BWCs during the evaluation study. With regard to official policies, a large portion of the agencies (83%) had official policies that required officers to either turn on their cameras at the start of their shift or at least turn them on when carrying out most official duties. Fifty‐three percent of the studies were carried out with agencies that also required officers to inform citizens that BWCs were turned on, although, for 37% of the studies, this was unknown.

However, policy directives to turn on cameras or to tell citizens about cameras do not fully capture the level of discretion that officers have with their cameras. While officers may be required to have their BWCs turned on, some studied agencies also allowed for officer discretion within this policy. We attempted to capture the level of discretion allowed by an agency's policy as reported by study authors using an ordinal measure: “no or low,” “moderate,” or “higher” discretion allowed. From what we could discern, 14 of the 30 eligible studies (47%) were conducted in agencies in which officers had no or low discretion as to when they could turn on or off their cameras. Policies in these agencies often specified only a handful of circumstances in which officers could turn off their cameras or included stringent rules and reporting requirements when officers turned off their cameras. Three studies involved agencies that seemed to have more “moderate” levels of discretion, in which guidance was provided in policy about some circumstances in which officers would be allowed to decide for themselves whether cameras could remain on or turned off. Finally, 30% of studies involved agencies that gave officers much higher levels of discretion as to their BWC usage. In those studies, agencies left it up to officers to decide whether to turn on their cameras and provided wide latitude in this decision. In 4 studies, the agency's discretion policy could not be discerned.

### 6.3 Study implementation

Two concerns dominate discussions of the implementation of BWC experiments and quasi‐experiments: contamination of treatment and control groups, and adherence to treatment conditions.

Many of the BWC studies have some form of contamination between treatment and control groups, which is particularly difficult to avoid and manage in BWC studies. Contamination often occurs in two ways in BWC evaluations. The first is when officers with BWCs in the treatment condition come into contact with officers not wearing BWCs in a control or comparison condition. This can occur when BWCs are randomly or otherwise allocated to some officers in an agency and not to others, and is particularly acute if BWC and non‐BWC officers are working in the same geographic areas. This type of contamination is difficult to control, as officers regularly provide other officers with assistance and backup. In these cases, non‐BWC wearing control officers may be affected by the presence of a BWC‐wearing officer and become more self‐aware or deterred, potentially making their behavior similar to treatment officers. Some study authors have tried to measure when this type of contamination occurs by examining calls for service or other events in which officers with and without BWCs are both present (see analytic examples given by Braga, Barao, et al., 2018; Braga, Sousa, et al., 2018; Lawrence & Peterson, 2019; Wallace et al., 2018; Yokum et al., 2019). Selecting comparison officers from a different location to avoid this type of contamination potentially makes the treatment and control groups noncomparable because the groups would then not be encountering the same contexts, environments, and types of events, as the treatment condition (and matching techniques may only alleviate some parts of the comparability problem).

Another form of contamination can occur when the same officers implement both treatment and control conditions. This happens, for example, when BWCs are randomly or otherwise allocated by shift. For example, on Tuesday, an officer may be assigned to wear a BWC because she is on a shift selected as the treatment condition for that day. However, on Wednesday, that same officer may be part of a control shift and thus not assigned to wear a camera. As Ariel and colleagues have discussed at length in multiple studies (see the Rialto experiment, Ariel et al., 2015, where this was first noted), using shifts reduces between‐group contamination described above and also allows for treatment and control environments and contexts to be similar. This approach, however, can suffer from within‐subject contamination; an officer on a non‐wearing camera day may act as if he or she is wearing a camera.

We examined each of the 30 studies for the possibility of contamination, both using the Cochrane Risk of Bias approach (see the Supporting Information Appendix G), as well as a more qualitative coding of contamination being more or less likely. We coded studies as more likely to have either form of contamination if the study had the same officers assigned to both treatment and control conditions, if the authors described the amount of contamination between treatment and control officers as being substantial (which we define here as treatment and control officers responding together to more than 25% of incidents), or the authors otherwise indicated that contamination might have influenced the results. As we indicate in Table 4, we believe that at least 80% of the BWC studies are more likely than not to have contamination bias. The Supporting Information Appendix G shows that that the likely impact of this type of bias are findings that move toward the null (BWCs may impact both those wearing and not wearing cameras in similar ways, thus muting the resulting impacts of treatment). However, this impact could also be unpredictable depending on the level, extent, type, and timing of this contamination.

Ariel et al. (2017) and Braga et al. (2019) have discussed contamination from a different perspective. Ariel et al. (2017) explore the idea of “contagious accountability,” which suggests that officers across an agency could be affected by their agency's use of BWCs even if they personally have not been assigned a camera, which could lead to broader positive gains. Braga et al. (2019) use the term “spillover effects,” and frame it as a possible diffusion of benefits (deterrence, self‐awareness, reduction in inappropriate or illegal behaviors) from officers wearing cameras to officers not wearing cameras. For our purposes, while we recognize that contamination might lead to broader benefits for agencies or their communities, such contamination may attenuate treatment effects in experimental research.

A second implementation concern is whether officers adhered to treatment conditions during the study (fidelity to treatment). This is a complicated concern with BWC research, given that fidelity to treatment presents a wide range of scenarios that were not always made clear by study authors. For example, at the most basic level, fidelity to treatment—“officer wearing BWCs”—could simply involve whether or not the officer was physically wearing a camera, regardless of whether the camera was turned on or whether an officer alerts a citizen of the presence of a camera. This issue is made more complicated because it is unclear whether the physical wearing of cameras alone impacts officers and citizens, or whether it is the activation of cameras that creates the effect (and, subsequently, the awareness by officers or citizens that they are being recorded). Even if we assume that wearing the camera required the camera to be activated, agencies had different policies regarding activation, which did not always align with researchers’ interests. Ariel's 10 anonymous studies are a case in point. In Ariel et al. (2016b), they describe that their study protocols “stripped officers of their discretion to decide when, where, and under which conditions BWCs would be applied” (p. 457), and yet, commanders in some of the 10 agencies studied gave their officers discretion—sometimes high levels of discretion—to wear and activate their cameras during the experiment. In these cases, official policies allowed for high levels of discretion, *and* fidelity to treatment was also low. Differently, agencies might allow for high levels of discretion as a matter of official policy, and if officers follow that policy in an experiment as planned, then fidelity would also be considered high.

We did attempt to capture in our coding whether officers in the treatment condition followed the instructions asked of them during the study. We coded studies according to whether treatment officers exhibited “lower” or “higher” levels of compliance with the treatment condition based on what was reported by authors. In some cases, study authors conducted empirical tests of compliance with BWC activation or compliance (see, e.g., Grossmith et al., 2015; Headley et al., 2017; Henstock and Ariel, 2017; Yokum et al., 2019) and reported on the level of compliance, whereas in other cases, study authors qualitatively reported on compliance levels (see Ariel et al., 2016b, and Ariel et al., 2018, for sites that had “no compliance” without empirical checks). For those in which noncompliance was measured, we coded anything over a 25% threshold as lower compliance or if authors themselves indicated lower compliance (37% of the studies). However, in many cases (43%), compliance could not be discerned at all, and we coded these as “unsure.” In 20% of the studies, it appeared that officers in the treatment group had higher compliance with treatment implementation.

### 6.4 Risk of bias

Risks of bias indicators were recorded at both the study and outcome levels. We adopted these assessments from the Cochrane risk‐of‐bias tool. The risk of bias assessment at the study‐level is shown as the Supporting Information Appendix G. We used three dimensions of study‐level bias from the Cochrane tool: Domain 1 (risk of bias arising from the randomization or other selection process); Domain 2 (risk of bias due to deviations from the intended interventions); and Domain 5 (risk of bias in the selection of the reported result). For each potential source of bias, we coded studies on the following scale: yes, probably yes, no, probably no, and no information. In the BWC arena, many of these risk‐of‐bias sources were unclear, and selected items from the Cochrane tool were dropped, given that the items were not relevant to the nature of the research designs in this area.^18^

As mentioned, 20 studies were RCTs, and 10 studies used a quasi‐experimental design (including time‐series studies that satisfied our criteria). Baseline equivalence was judged as nonproblematic for all of the RCTs and many of the quasi‐experimental designs, with the exception of four studies in which the effect of this nonequivalence was either unpredictable (3 studies) or likely to bias the results toward the null (1 study). There were no widespread violations of the randomization process that we could ascertain for most of the experiments. Most RCTs involved all units, but in some cases, individuals volunteered for the study, and then all volunteers were randomly assigned to treatment and control groups (see, e.g., Braga, Barao, et al., 2018; Braga, Sousa, et al., 2018; Jennings et al., 2015; Sousa et al., 2016). While studying only volunteers raises challenges to external validity, risk of bias from the randomization was not likely.

Several forms of unit assignment to treatment were used across quasi‐experiments. For example, the Tampa study (Jennings et al., 2017) compared officers who had volunteered to be in the treatment group and wear cameras to other officers who had not volunteered and were not wearing cameras, using propensity score matching to find a comparable sample. In the Mesa study (Ready & Young, 2015), the treatment group consisted of a mix of volunteers and officers mandated to wear BWCs, and the control group was found by matching officers on race, gender, and age. In the Hallandale study (Headley et al., 2017), the treatment group consisted of volunteers, sergeants required to wear cameras, and a group randomly selected across two stages of selection. The comparison group included officers not selected during any of these processes (although no matching techniques or statistical controls were reported). In the case of the non‐Maryvale Phoenix study (Katz et al., 2019), researchers examined randomly selected treatment and control officers but also included an additional group of voluntary treatment officers (see the previous description in Section 6.1). In other cases, officers were assigned to treatment groups because they worked in a geographic area selected for intervention (see Ariel, 2016, 2017; Katz et al., 2015; Mitchell et al., 2018), and were then compared to officers in other areas in which cameras were not assigned, which could lead to baseline differences between groups. In two cases, agencies were compared during time periods in which they did not use BWCs and later times in which they did (Koslicki et al., 2019; Stolzenberg et al., 2019). These complexities can make it difficult to adequately match officers to treatment officers on important characteristics, or to predict the direction of bias that may result.

Internal validity is also likely muted in several studies by the high likelihood of contamination and challenges with fidelity in BWC studies as aforementioned, which seems most likely—at least a priori—to bias estimated effects toward the null. However, we did not find a great deal of evidence as to attrition or loss of study participants in the vast majority of these studies.

At the outcome level, we used two dimensions of risk of bias, again adopted from the Cochrane tool. These were Domain 3, which assesses the risk of bias from missing outcome data, and Domain 4, which assesses the risk of bias in the measurement of the outcome (Supporting Information Appendix H). In terms of risk of bias from missing data, we did not believe there were widespread missing data problems in studies, and there was little reason to believe that outcomes might be missing for some participants and not others. The nature of these outcomes would make both of these risks unlikely.

Regarding risk‐of‐bias in outcome measurement (Domain 4), however, our assessment was more equivocal. Depending on the particular outcome being measured, the measurement or ascertainment of the outcome could have differed between intervention groups. We note that our determinations are based on a logical assessment on our part because study authors do not assess this directly, and therefore our assessments are debatable. For example, for studies examining the use of force as an outcome, we assessed the risk of bias as “probably yes” in all cases. For this outcome, the wearing of the BWC could prompt officers to report their uses of force more so than not relative to the control officers, and potentially more so for minor (and even authorized) uses of force. Similarly, officers may be more likely to report assaults and resistance against them relative to control officers, given that those actions are now caught on camera. With regard to citizen complaints, this was more ambiguous, but we also assessed these outcomes as “probably yes,” given what we know from officer and citizen survey research (see Lum et al., 2019). For example, citizens might be more likely to file reports against officers when they know that officer misconduct was captured by BWCs, but they might be less likely to file complaints if they feel that the behaviors will not appear sufficiently serious on a BWC recording or if the recording might reflect badly on their own behavior.

We also assessed outcomes of self‐initiated activities (officer proactivity), stop‐question‐and‐frisks, pedestrian stops, and traffic stops as also “probably yes” with regard to the risk of bias in measurement. Again, these are based on our logical assessment and not information in the studies. Officers in control conditions do not have to turn on BWCs when engaging in these activities, and empirical research by Lum, Koper, Wu, Johnson, and Stoltz (2020) indicates that up to 50% of proactivity may not be even reported to dispatchers. However, those wearing BWCs may be more inclined to report when they carry out these activities (or may be required to report them when wearing a BWC).

For other outcomes, this particular risk of bias in measurement seems less probable. For example, with arrests, citations, incident reports, and dispatched calls for service, having a BWC may influence the *occurrence* of these outcomes but not their *measurement*. Once an arrest is made, a citation is given, an incident report is written, or a call is dispatched, there is no reason to suspect those with BWCs may then somehow be able to undo or not report those activities. Nor would an RMS system or a supervisor reviewing a report from an officer with a BWC be able to do the same, even if knowing that officer had a BWC. For incident reports, officers may be asked to revise their reports based on reviewing their BWC footage, but the existence of the report is unaffected. The same is true with the measurement of dispatched calls for service.

### 6.5 Synthesis of results

Across the 30 studies, 12 outcome constructs were identified with a total of 112 outcomes across the 30 studies (shown in the Supporting Information Appendix I) and 116 effect sizes calculated. Again, the Supporting Information Appendix E details how effect sizes were calculated, and the Supporting Information Appendix F includes the script (R Code) file for all analyses. All individual calculated effects are provided on the OSF site for this review. Table 5 shows the overall mean percent change by construct, derived from the RIRR. A RIRR reflects the difference‐in‐difference change in counts. Values of RIRR greater than 1 indicate that the BWC count increased relative to the control count. Values of RIRR <1 indicate that the BWC count decreased relative to the control count. The RIRR values were converted to percentages with the following formulas, depending on whether the value was above or below 1. Negative percent change indicates that the BWC count decreased relative to the control count and visa‐versus for positive percent change.

ForRIRR>1;Percentchange=(RIRR−1)×100.

ForRIRR<1;Percentchange=(1−RIRR)×100.

**Table 5:** Overall mean percent change by construct and associated statistics

		95% CI							
Construct	Mean % change	Lower	Upper	*k*	*z*	*p*(*z*)	*Q*	*p*(*Q*)	*τ*
Use of force	−6.8	−19.5	7.9	26	−0.941	.347	82.822	.000	0.303
Complaints against officer	−16.6	−30.0	−0.7	22	−2.033	.042	26.109	.202	0.200
Assault on officer/officer injuries/resistance	15.9	−4.9	41.3	15	1.464	.143	15.071	.373	0.133
Arrests	−3.9	−12.7	5.8	13	−0.813	.416	159.762	.000	0.146
Officer‐initiated CFS	3.8	−5.2	13.5	8	0.803	.422	17.320	.015	0.097
Dispatched calls for service	2.6	−3.0	8.6	6	0.907	.365	322.632	.000	0.054
Traffic stops or traffic tickets	−5.0	−33.1	34.8	5	−0.289	.772	362.696	.000	0.372
Field interviews or stop and frisk	−12.0	−37.5	24.0	4	−0.729	.466	20.943	.000	0.330
Incident reports	−8.0	−21.7	8.0	3	−1.018	.309	46.710	.000	0.127
Response time	−0.2	−1.7	1.3	3	−0.238	.812	0.697	.706	0.000
Non‐traffic citations	6.4	5.8	7.1	2	19.031	.000	0.382	.536	0.000
Time on scene	−4.6	−12.0	3.4	1	−1.149	.251			

*Note:* CI = confidence interval; *k* = number of effect sizes; *z* = *z*‐test; *p*(*z*) = *p*‐value for *z*‐test; *Q* = homogeneity statistic; *p*(*Q*) = *p*‐value for homogeneity statistic; *τ* = square‐root of the random effects variance component.

In summary, only two constructs appear to be statistically significant—complaints and non‐traffic citations. The research indicates that the use of BWCs by police officers reduces the number of complaints against officers and increases officers’ use of non‐traffic citations. However, the non‐traffic citations finding is based on only two studies and is thus very preliminary. The findings for the other constructs, such as use of force, assaults on officers, arrests, officer proactivity, and citizen calls for service, are not statistically significant and much more ambiguous or heterogeneous. Overall, there is not a clear impact of BWCs on any of these behaviors. We discuss the findings for each construct below, along with detailed explanations for each construct that are important for interpreting each effect.

#### 6.5.1 Use of force

Use of force was the most frequently examined outcome in eligible studies and reflected the main impetus behind the adoption of BWCs. As discussed above, BWCs were considered as a possible tool to increase the accountability of police officers regarding their uses of force, especially against racial and ethnic minorities. Previous narrative reviews (see Lum et al., 2019) found heterogeneous effects of BWCs on the use of force (showing BWCs can lead to increases, decreases, or no effects on the use of force), leading some to believe that other factors (such as officer discretion or fidelity of treatment) may contribute to these findings (Gaub & White, 2020; White, 2019).

**Figure 1:**
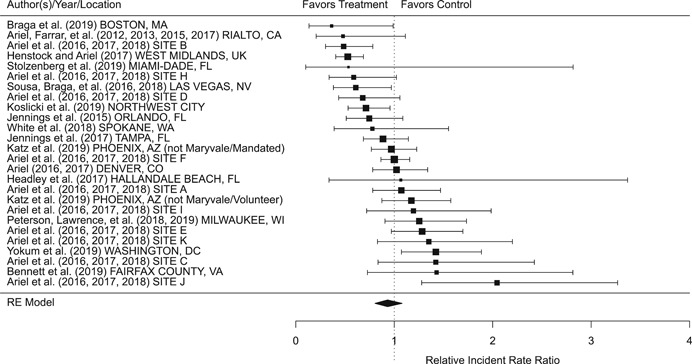
Meta‐analysis results of Body‐worn cameras (BWCs) and use of force

Twenty‐six out of the 30 studies in our review reported on this outcome, and the forest plot for this construct is shown as Figure 1. To those unfamiliar with forest plots, for our analyses, points plotted to the left of 1 indicate a “treatment effect,” which always indicates a relative reduction in the construct for officers wearing BWCs for all of the forest plots shown in this review. We note that we do not assign any normative value to the term “favors” treatment or “reduction” for each construct. These terms only refer to the direction of the effect. For each study effect, the squares reflect the study‐level effect size, and the horizontal line reflects the 95% confidence interval. The size of the effect size square reflects the weight given to each study (inversely, the wider the confidence interval, the smaller the weight and conversely, the larger the standard error). The diamond at the bottom of the table shows the random‐effects mean effect size and confidence interval (this is the row labeled “RE Model”). For our plots, the upper bound of the 95% confidence intervals has been censored at +10 to improve the readability of the plots and ensure consistent scaling across plots.

For use of force, the RIRR = 0.932, which translates to a 6.8% relative reduction in use of force incidents in the treatment group compared to the control group on average (as shown in Table 5). This finding is not statistically significant, with a 95% confidence interval that ranges from roughly a 20% reduction to roughly an 8% increase in use of force. The distribution is highly heterogeneous, indicating variability in the underlying effects across studies. This suggests substantial uncertainty regarding the effectiveness of BWCs in reducing use of force. We will explore possible explanations for this heterogeneity in our moderator analyses below.

#### 6.5.2 Complaints

**Figure 2:**
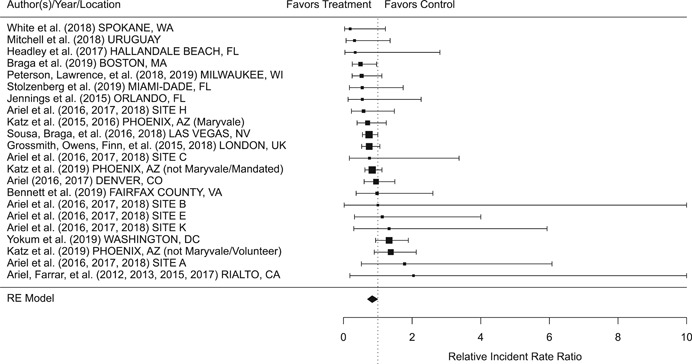
Meta‐analysis results of Body‐worn cameras (BWCs) and complaints

The second most frequently studied outcome in eligible BWC studies is complaints. We might expect complaints to decline when officers wear BWCs because it improves officer or citizen behavior, or because it affects citizen reporting behavior (see Lum et al., 2019). Twenty‐two out of the 30 studies in our review reported on the impact of BWCs on complaints, and the forest plot of this outcome is shown as Figure 2. The RIRR = 0.834, which translates to a 16.6% relative reduction in the number of complaints in the treatment condition compared to the control condition. This finding is the only main finding aside from citations^19^ that is statistically significant (*p* = .042). This finding is consistent with multiple prior reviews—it appears that for reasons unclear from this research, officers with BWCs have significantly fewer complaints lodged against them than officers who do not wear BWCs. In contrast to the highly heterogeneous findings for use of force, the distribution of effects for complaints is not statistically significantly heterogeneous, with a fairly small random‐effects dispersion parameter, *τ*, suggesting little to only modest variability in effects across studies.

#### 6.5.3 Assaults on officers/officer injuries and resistance to officers

Next, we turn to the impact of BWCs on assaults against officers (some studies also include officer injuries from these assaults) and citizens physically resisting officers. Unlike use of force, these constructs are more suggestive of citizen behaviors rather than officer behaviors (although Ariel et al., 2018, suggest these outcomes can result from the interaction between officer and citizen behaviors). The idea is that citizens are less likely to assault or resist officers when they know they are being recorded or see a BWC pointed at them. We initially identified this construct as two separate constructs: assaults on officers (to include measures of officer injuries from those assaults) and resistance (during arrest). We decided to combine these two constructs because they often overlap (i.e., an assault is deemed to take place on an officer while a person is resisting arrest). Additionally, only two studies (Headley et al., 2017; Katz et al., 2015) measured resistance.

**Figure 3:**
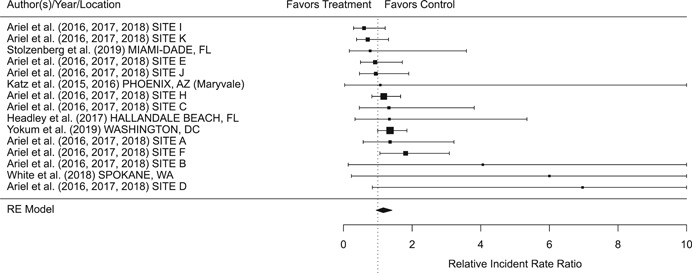
Meta‐analysis results of body‐worn cameras (BWCs) and assaults against officers/officer injuries and resistance against officers

Fifteen out of the 30 studies examined this outcome, and the results are presented in the forest plot of Figure 3. Note that almost all of these findings come from Ariel et al.'s 10 anonymous studies. The overall RIRR = 1.148, which translates to a 15.9% relative increase in assaults or resistance against officers wearing BWCs. However, the finding is not statistically significant, indicating a lack of evidence supporting the beneficial (or harmful) effects of BWCs on this outcome. The confidence interval is wide, and the distribution is heterogeneous, indicating uncertainty in the effect of BWCs on this outcome.

#### 6.5.4 Arrests

**Figure 4:**
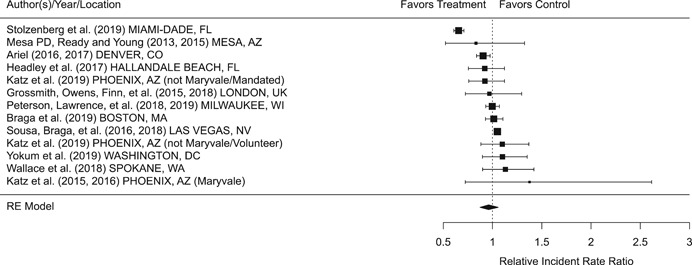
Meta‐analysis results of body‐worn cameras (BWCs) on arrests

Researchers have also examined whether BWCs affect the arrest decisions of police officers. For example, officers wearing BWCs might act more legalistically in their decision to arrest individuals, possibly reducing their discretion and increasing their use of arrest, given their awareness of being recorded (see discussions in Ariel et al., 2017; Rowe, Pearson, & Turner, 2018). Thirteen out of the 30 studies examined this outcome, and the forest plot for arrest outcomes is shown in Figure 4. The RIRR = 0.961, which translates to a 3.9% relative reduction in arrest incidents in the treatment condition compared to the control condition. This finding is not statistically significant and it is also highly heterogeneous, suggesting that BWCs may increase arrests in some contexts and decrease them in others with uncertainty about any typical effect across implementations.

#### 6.5.5 Officer‐initiated calls for service (general proactivity)

Officer (or “self”)‐initiated calls for service often reflect officer proactivity, which includes a wide range of actions. As already discussed, some have hypothesized that BWCs might cause officers to reduce their proactivity to avoid additional scrutiny (whether the proactivity is controversial or not). The challenge of examining this measure as an outcome is that proactivity is not regularly reported by officers (Lum et al., 2020 found that up to 50% of proactivity by officers may go unreported).

**Figure 5:**
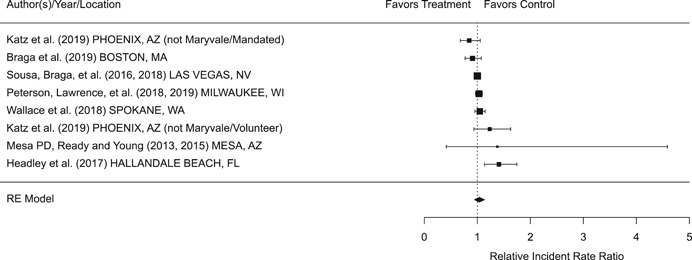
Meta‐analysis results of body‐worn cameras (BWCs) and officer‐initiated calls for service

For this review, we examined three types of self‐initiated activity reported in eligible studies: a general measure of self‐initiated calls for service, traffic stops or traffic tickets, and field interviews or stop and frisks.^20^ Eight out of the 30 eligible studies reported a general measure of officer‐initiated calls for service, and the forest plot for these outcomes is shown as Figure 5. The RIRR = 1.038, which translates to a 3.8% relative increase in officer self‐initiated calls for service in the BWC treatment group compared to the control condition. This finding is not statistically significant, but it is heterogeneous, suggesting uncertainty in the typical effect across BWC implementations.

#### 6.5.6 Traffic stops or tickets

**Figure 6:**
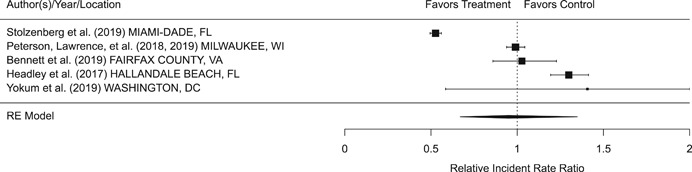
Meta‐analysis results of body‐worn cameras (BWCs) and traffic stops or tickets

Traffic stops and tickets can result from both self‐initiated and citizen‐initiated calls for service, but they often represent a significant proportion of proactivity as practiced by the police (Lum et al., 2020). As with the more general measure of proactivity above, we did not find an impact of BWCs on traffic stops or tickets. However, only five studies reported this outcome, and the mean RIRR = 0.950 suggests a 5% relative reduction in traffic stops or traffic tickets in the treatment group compared to the control group (Figure 6). This finding is not statistically significant, and it is highly heterogeneous. Caution should be taken in inferring anything from this finding, given that it is based on only five studies.

#### 6.5.7 Field interviews or stop and frisk

**Figure 7:**
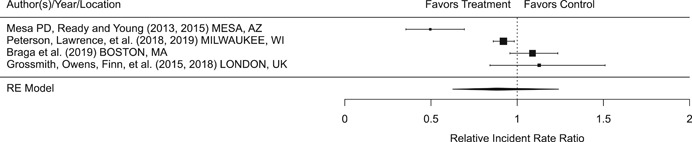
Meta‐analysis results of body‐worn cameras (BWCs) and field interviews or stop and frisks

Only 4 out of 30 studies examined the impact of BWCs on another form of proactivity—field interviews and stop‐and‐frisks (also called “pedestrian stops”). Figure 7 shows the forest plot for this analysis. The RIRR = 0.880 translates to an average 12% relative reduction in field interviews or stop and frisk reports in the treatment group compared to the control group. As with general measures of proactivity, this finding is not statistically significant and is highly heterogeneous. Caution should be taken in inferring anything from this finding, given that it is based on only four studies.

#### 6.5.8 Dispatched calls for service

One possible measure of citizen behavior is the number of dispatched calls for service (which indirectly estimates the frequency with which citizens call the police). Some citizens might not want to be videotaped, given their own self‐awareness or fears about the suspect or the police, and may choose not to call the police if they know that responding police are wearing BWCs. However, this effect relies on a tenuous assumption that citizens know their officers in their jurisdictions were wearing BWCs. McClure et al. (2017) and White et al. (2017) found that citizens may not even remember whether officers they interacted with were wearing BWCs (also hinted at by Goodison and Wilson, 2017). Additionally, any impacts that BWCs might have on citizen reporting would likely affect calls to both treatment and control officers if they were all working in the same geographic area. Some studies that examine dispatched calls for service do not hypothesize about the effects of BWCs on citizen calls. For example, Ariel (2016a, 2016b) used calls for service as a stabilizing variable for their analysis, to create balanced treatment and control groups at baseline so to examine other effects of BWCs.

**Figure 8:**
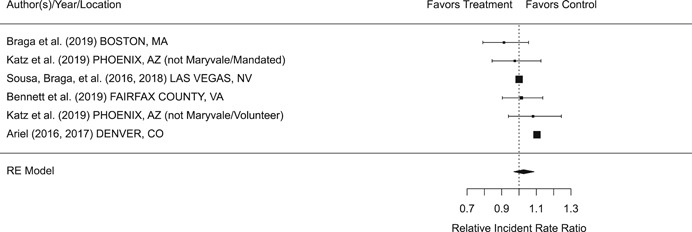
Meta‐analysis results of body‐worn cameras (BWCs) on dispatched calls for service

Despite these uncertainties, we examine this construct but caution readers in interpreting the findings. Six of the 30 eligible studies examined the impact of BWC use on dispatched calls for service, and we present the forest plot of these estimated effects in Figure 8. The RIRR = 1.026, which translates to a 2.6% relative increase in dispatched calls for service for the treatment condition overall. This finding is not statistically significant and is highly heterogeneous.

#### 6.5.9 Other outcomes

Incident reports, response time, non‐traffic citations, and time on scene were also collected as part of this systematic review. Each of these appeared in three or fewer studies, as shown in the last four rows of Table 5. Among these outcomes, there was a statistically significant shift only for non‐traffic citations, with those officers using BWCs writing more non‐traffic citations than officers not wearing BWCs. Nonetheless, given the low number of tests for these outcome categories, we believe it is premature to draw any claims about the effects of BWCs on these outcomes.

#### 6.5.10 Summary of results

**Figure 9:**
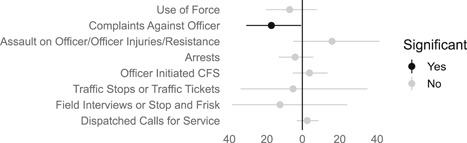
Summary of results

Figure 9 presents mean percent changes and confidence intervals of all findings, excluding the “other outcomes” in a single graphic. Overall, only one construct shows a statistically significant mean % reduction across studies—complaints. The findings for the other constructs, such as use of force, assaults on officers, arrests, officer proactivity, and citizen calls for service, are heterogeneous and not statistically significant.

### 6.6 Sensitivity analysis

Two sensitivity analyses were conducted for this review. First, we re‐ran the overall analyses without Ariel et al.'s 10 anonymous studies (see Ariel et al., 2016a, 2016b, 2017, 2018). Because these studies were reported collectively as a “global multisite randomized controlled trial,” many aspects of the individual sites and analyses were difficult to discern or not reported, such as where and when the intervention occurred, the context or background of the BWC implementation, or specific aspects of BWC implementation. We report the overall mean percent change and associated statistics for those constructs measured in Ariel et al.'s studies (complaints, use of force, and assaults on officers/resistance) in Table 6 with those sites removed (the other construct findings from Table 5 remain the same). Findings did not change for the most part; the findings for complaints continued to remain significant and in the same direction. The relative reduction in use of force almost doubles to 12.8%, although this finding continues to be nonsignificant and heterogeneous. The removal of Ariel's 10 global studies also leads to an effect of borderline statistical significance (*p* level just above .05) on assaults and resistance against officers, with the mean % change increasing dramatically to a 34.2% relative increase in assaults and resistance.

**Table 6:** Overall mean percent change for constructs and associated statistics without Ariel et al.'s 10 global studies

		95% CI							
Construct	Mean % change	Lower	Upper	*k*	*z*	*p*(*z*)	*Q*	*p*(*Q*)	*τ*
Complaints against officer	−18.4	−32.7	−1.0	16	−2.062	.039	23.535	.073	0.222
Use of force	−12.8	−27.1	4.4	16	−1.490	.136	48.093	.000	0.278
Assault on officer/officer injuries/resistance	34.2	−0.1	80.1	5	1.956	.050	1.322	.858	0.000

*Note:* Percent change is based on the relative incident rate ratio (RIRR). For values of RIRR >1, the percent change is RIRR − 1. For values of RIRR <1 it is 1 − RIRR. A RIRR reflects the difference‐in‐difference percent change in counts. Negative numbers reflect lower counts for the BWC condition. *k* = number of effect sizes; *z* = *z*‐test; *p*(*z*) = *p*‐value for *z*‐test; *Q* = homogeneity statistic; *p*(*Q*) = *p*‐value for homogeneity statistic; *τ* = square‐root of the random effects variance component.

Second, we re‐ran the main effects analysis after removing the Mesa, Arizona Study (Mesa Police Department, 2013; Ready & Young, 2015). The Mesa study uses field contact reports that were “completed by the officers on selected days every time the officers had contact with a citizen. Five days per month were randomly selected for officers to fill out the field contact reports, one day for each police precinct” (Ready & Young, 2015, p. 449). Authors, therefore, measured officer behavior in this study by asking officers to report whether or not they issued non‐traffic citations, gave warnings, conducted stop‐and‐frisks, made arrests, or self‐initiated any other activities. This type of data collection was markedly different from other studies that used police administrative data for the same constructs.

Table 7 presents the new analysis with the Mesa study removed. (again, the other findings from Table 5 remain the same). Substantive inferences do not change from the main effects, and all mean percent changes are nonsignificant except for citations, which now only reflects a single effect size. Removing the Mesa study, however, does produce a notable mean percent change for field interviews and stop and frisk changes from a 12% reduction to a 1% increase.

**Table 7:** Overall mean percent change by construct and associated statistics excluding the Mesa, Arizona, Study

		95% CI							
Construct	Mean % change	Lower	Upper	*k*	*z*	*p*(*z*)	*Q*	*p*(*Q*)	*τ*
Arrests	−3.4	−12.5	6.6	12	−0.691	.490	158.832	.000	0.148
Officer‐initiated calls for service	3.6	−5.4	13.5	7	0.765	.444	17.047	.009	0.098
Field interviews or stop and frisk	1.0	−12.0	15.9	3	0.144	.885	6.456	.040	0.096
Citations	6.4	5.8	7.1	1	19.028	.000			

*Note:* Percent change is based on the relative incident rate ratio (RIRR). For values of RIRR >1, the percent change is RIRR − 1. For values of RIRR <1, it is 1 − RIRR. A RIRR reflects the difference‐in‐difference percent change in counts. Negative numbers reflect lower counts for the BWC condition. *k* = number of effect sizes; *z* = *z*‐test; *p*(*z*) = *p*‐value for *z*‐test; *Q* = homogeneity statistic; *p*(*Q*) = *p*‐value for homogeneity statistic; *τ* = square‐root of the random effects variance component.

### 6.7 Moderator analyses

BWC researchers have argued that findings from BWC studies may be influenced by a number of moderating factors including aspects of the research designs, official BWC policies, and the way BWCs are implemented (see, e.g., Ariel et al., 2016b; Braga et al., 2019; Gaub & White, 2020; Katz et al., 2019). Given these concerns, we proposed in the protocol to carry out a number of post‐hoc moderator analyses based on the availability of information found in eligible studies. We ran several moderator analyses on the four outcomes that appear most frequently across these studies: use of force, complaints, arrests, and assaults on officers. For these outcomes, we examine whether outcomes vary based on research design, the unit of randomization, agency context and background, year of adoption, discretion in BWC use, contamination between treatment and control officers, intervention fidelity, and the research team involved in these studies. These findings should be viewed cautiously, given that the sample size for each analysis is small.

#### 6.7.1 Randomized experiments versus quasi‐experiments

Table 8 shows the mean percent change between BWC and non‐BWC use, comparing randomized experiments and quasi‐experimental studies. Per Campbell Collaboration conventions, we show all of the findings for each construct for this specific moderator analysis (we do not do so for the other moderator analyses), but again note the small *k* for most of these outcomes. For use of force, complaints, and assaults on officers/resistance, there are no significant differences in the mean RIRR between RCTs and quasi‐experiments, though the effect estimates are somewhat stronger for complaints and use of force (showing greater reductions) in RCTs.

**Table 8:** Mean percent change by design (RCTs vs. quasi‐experiments) and construct

				95% CI			
Construct	Design	*k*	Mean % change	Lower	Upper	*Q*_Between_	*p* (*Q*_Between_)
Arrests	QE	6	−13.5	−24.0	−1.5	3.889	.049
	RCT	7	2.4	−7.9	13.8		
Complaints against officer	QE	7	−10.8	−35.2	22.9	0.261	.609
	RCT	15	−19.3	−34.8	0.0		
Dispatched calls for service	QE	3	9.3	5.4	13.4	12.974	.000
	RCT	3	−0.6	−4.2	3.1		
Incident reports	QE	1	−19.6	−24.5	−14.4	46.705	.000
	RCT	2	0.0	−0.2	0.1		
Officer‐initiated calls for service	QE	3	34.1	13.3	58.8	11.577	.001
	RCT	5	0.1	−0.1	0.2		
Assault on officer/officer injuries/resistance	QE	3	4.7	−61.5	184.9	0.040	.841
	RCT	12	16.3	−5.3	42.8		
Response time	QE	1	−0.5	−2.4	1.4	0.253	.615
	RCT	2	0.3	−2.1	2.7		
Field interviews or stop and frisk	QE	1	−50.4	−66.2	−27.2	11.679	.001
	RCT	3	1.0	−12.0	15.9		
Traffic stops or traffic tickets	QE	3	−11.3	−45.1	43.2	0.275	.600
	RCT	2	10.7	−43.9	118.4		
Use of force	QE	7	−3.1	−28.5	31.4	0.091	.764
	RCT	19	−8.1	−22.8	9.3		

*Note:* *k* = number of effect sizes; *Q*_Between_ is the test of the difference between the means with associated significance value *p*(*Q*_Between_).

Abbreviations: QE, quasi‐experimental study; RCT, randomized controlled trial.

For arrests, however, the differences between quasi‐experimental and RCT studies are significant; quasi‐experimental studies are more likely to show that BWCs yield relative reductions in arrest (the RCTs show a null effect). Significant differences were also found between quasi‐experiments and RCTs for dispatched calls for service and officer‐initiated calls for service. For both, quasi‐experiments seemed to indicate relative increases in dispatched calls or self‐initiated activity for officers wearing BWCs, whereas for RCTs, the findings show a null effect. Other outcomes (incident reports, response time, and field interviews/stop and frisks) are harder to judge because each has only one quasi‐experimental effect size for comparison to multiple effect sizes from RCTs.

#### 6.7.2 Unit of assignment

As already discussed, Ariel and colleagues argue that randomization by shift as opposed to randomization by officer might be better able to control between‐subject contamination threats. However, randomization by shifts can suffer from within‐subject contamination. Perhaps outcome differences between shift‐based randomization and officer‐based randomization might reveal more insight into this discussion. Table 9 shows the mean percent change between BWC and non‐BWC use, comparing studies that allocated treatment and control by shifts, officers, or another unit (such as geographic areas). There are no significant differences in the mean RIRR between studies that use shift, officer, or another unit of analysis. It is unclear what this means for which method performs better with regard to contamination, a subject requiring much more research beyond the scope of this review.

**Table 9:** Mean percent change by unit of assignment

	Unit of assignment			95% CI			
Construct		*k*	Mean % change	Lower	Upper	*Q*_Between_	*p* (*Q*_Between_)
Complaints against officer	Officer	9	−16.7	−33.8	4.8	0.768	.681
	Other	6	−25.9	−50.1	9.9		
	Shift	7	0.7	−42.9	77.4		
Assault on officer/officer injuries/resistance	Officer	2	39.9	−12.8	124.2	0.837	.658
	Other	3	4.7	−62.0	188.0		
	Shift	10	9.5	−15.0	41.0		
Use of force	Officer	9	−7.2	−28.0	19.6	0.003	.998
	Other	5	−6.0	−37.2	40.7		
	Shift	12	−7.1	−25.7	16.1		

*Note:* *k* = number of effect sizes; *Q*_Between_ is the test of the difference among/between the means with associated significance value *p*(*Q*_Between_).

#### 6.7.3 Agency context and background

White (2019) and Gaub and White (2020) argue that agency context or background can potentially moderate the impacts of BWCs on outcomes. White (2019) notes that “troubled agencies that adopt BWCs may see the Rialto‐like declines in use of force and citizen complaints because there is much room for improvement. Highly professional agencies with robust employee selection, training, policy, supervision, and accountability processes will probably not experience those same large declines because there is less room for improvement” (p. 90).^21^ To test White's hypothesis, we coded whether, in the two years prior to camera adoption, the studied agency or jurisdiction experienced a collaborative reform, consent decree, U.S. Department of Justice review, or any other official review due to a sentinel event (like an officer‐involved shooting) as reported by the study authors. As Table 4 indicated, only five of our eligible studies noted such an occurrence in the jurisdiction studied. These locations, which were all in the United States, included Hallandale Beach (Headley et al., 2017); Las Vegas (Braga, Barao, et al., 2018; Braga, Sousa, et al., 2018; Sousa et al., 2016); Miami‐Dade (Stolzenberg et al., 2019); Milwaukee (Lawrence & Peterson, 2019; Peterson et al., 2018); and Spokane (Wallace et al., 2018; White et al., 2018). When comparing the studies of these agencies against others, we do not find statistically significant differences in mean percent change for use of force, complaints, arrests, or assaults on officers/resistance (Table 10). However, there are tentative indications that reductions in complaints were greater in agencies that had these concerns prior to BWC adoption. Although not statistically significant, BWC use resulted in a relative reduction of 38% in complaints in agencies in which background concerns were mentioned, compared to a relative reduction in 10% in agencies in which such concerns were not mentioned (*p* = .086). As noted, these findings should be interpreted cautiously because agency background issues were not reported for most of the studies.

**Table 10:** Mean percent change based by collaborative reform/sentinel event and construct

			Mean % change	95% CI			
Construct	Reform	*k*		Lower	Upper	*Q*_Between_	*p* (*Q*_Between_)
Arrests	No or not mentioned	8	−0.5	−12.9	13.6	0.534	.465
	Yes mentioned	5	−7.5	−19.8	6.7		
Complaints against officer	No or not mentioned	17	−9.8	−25.7	9.5	2.950	.086
	Yes mentioned	5	−37.9	−57.6	−9.2		
Assault on officer/officer injuries/resistance	No or not mentioned	12	15.4	−6.2	41.9	0.014	.904
	Yes mentioned	3	22.9	−54.7	233.2		
Use of force	No or not mentioned	21	−5.9	−19.9	10.5	0.125	.724
	Yes mentioned	5	−13.1	−42.5	31.3		

*Note:* *k* = number of effect sizes; *Q*_Between_ is the test of the difference among/between the means with associated significance value *p*(*Q*_Between_).

#### 6.7.4 Year of adoption

The 2010s were a fast‐moving decade with regard to BWC adoption, and by the middle of the decade, adoption had seemed to reach its half‐way mark (Hyland, 2018). It could be the case that officers within early‐adopting agencies were more resistant to BWCs, and their early adoption could have been due to a sentinel event (Gaub and White, 2020). Perhaps officers in later‐adopting agencies had already seen others using them and were more amenable to adoption given stories they had heard about BWCs protecting officers from frivolous complaints. Given that we knew the year of the initial adoption of BWCs by agencies for 20 of the studies, we ran a meta‐regression model for year of BWC adoption for arrests, assaults on officers, complaints, and use of force. Table 11 shows the linear regression coefficient of the effect that year has on the effect size for each construct (note that the regression coefficient reflects the predicted change in the logged RIRR for each year increase). At least within these twenty studies, findings do not significantly change depending on the year when BWCs were initially implemented in an agency.

**Table 11:** Meta‐regression for year BWC is initially implemented by construct predicting logged RIRR

		95% CI			
Construct	Regression coefficient	Lower	Upper	*z*	*p*(*z*)
Arrests	−0.022	−0.067	0.023	−0.960	.337
Complaints against officer	−0.006	−0.090	0.078	−0.139	.889
Assault on officer/officer injuries/resistance	−0.390	−1.468	0.688	−0.710	.478
Use of force	0.096	−0.024	0.216	1.572	.116

*Note:* *z* = *z*‐test; *p*(*z*) = *p*‐value for *z*‐test.

#### 6.7.5 Level of discretion in operating BWCs

We also examined whether there were differences in findings based on differences in the levels of discretion as dictated by policy or mandate for agencies studied. We coded studies according to whether officers had no to low, moderate, or higher levels of discretion when it came to operating their BWCs (see descriptions in Section 6.2). Table 12 shows the mean percent change for our four selected outcome constructs differentiated by level of discretion. The table also shows results from a meta‐regression for the linear relationship between discretion level and the logged RIRR for each outcome measured. For complaints, arrests, and assaults on officers/resistance, there is no statistically significant relationship between the officers’ level of discretion in operating BWCs and those outcomes. For use of force, however, a significant effect was found. The more restrictive an agency's policies were regarding officers’ discretion in BWC use, the greater the reduction in use of force that was found. It is important to note, however, that this does not mean that “no or low” discretion policies significantly reduce use of force. The regression finding is more limited in reflecting a decrease in the logged RIRR (as estimates shift from showing increases in force to showing decreases in force) when officers have less discretion, despite uncertainty as to whether there is a meaningful decrease for the more restrictive policies (as reflected in the confidence intervals for those policies). The credibility of this finding is increased by the lack of an effect for the three other outcomes, as these were not theoretically expected to be affected by discretion. These findings align with Ariel et al.'s (2016a) study, which found that wearing BWCs is more likely to be associated with increases in the use of force when officers are allowed higher levels of discretion with their BWC use. Additional studies, however, are needed to establish whether use of force can be reduced when an agency restricts officer discretion in how they use BWCs.

**Table 12:** Mean percent change based by discretion and construct and the meta‐regression for the linear relationship between discretion and logged RIRR

				95% CI				
Construct	Discretion	*k*	Mean % change	Lower	Upper	Regression coefficient	*z*	*p*(*z*)
Arrests	Higher	2	2.9	−23.8	39.0	−0.037	−0.464	.642
	Moderate	3	−15.6	−29.8	1.3			
	No or low	7	−0.7	−12.8	13.0			
Complaints against officer	Higher	5	−2.2	−49.6	90.0	−0.080	−0.507	.612
	Moderate	3	−35.8	−57.8	−2.5			
	No or low	11	−9.0	−28.2	15.2			
Assault on officer/officer injuries/resistance	Higher	8	3.8	−22.2	38.4	−0.121	−1.145	.252
	Moderate	1	−22.5	−83.8	270.7			
	No or low	6	32.8	−2.8	81.5			
Use of force	Higher	8	22.1	−4.0	55.4	0.182	2.244	.025
	Moderate	2	−59.2	−84.2	5.7			
	No or low	12	−15.8	−30.7	2.3			

*Note:* The regression coefficient is based on a meta‐regression of the linear relationship between discretion and logged relative incident rate ratio (RIRR). *k* = number of effect sizes; *z* = *z*‐test; *p*(*z*) = *p*‐value for *z*‐test.

#### 6.7.6 Contamination

One implementation challenge encountered by many BWC studies is the risk of contamination. Contamination may reduce the ability to find discernible differences between treatment and control conditions if officers in control conditions who are not wearing BWCs come into contact with officers wearing BWCs, or if the same officers participate in treatment and control conditions. Table 13 displays the moderator analysis for contamination. Again, given that a large proportion of BWC studies likely experience some form of contamination, readers should take this analysis only as suggestive. Studies that had a lower risk of contamination were few in number but more likely to find that BWCs led to a relative reduction in the construct. These differences, however, were only statistically significant for arrests (studies less likely to have contamination were more likely to see reductions in arrests with BWC use). Much more analysis is needed to determine why this might be the case.

**Table 13:** Mean percent change by whether it is more or less likely for contamination and construct

				95% CI			
Construct	Contamination	*k*	Mean % change	Lower	Upper	*Q*_Between_	*p* (*Q*_Between_)
Arrests	Less likely	3	−20.2	−30.3	−8.8	9.463	.002
	More likely	10	2.2	−6.0	11.1		
Complaints against officer	Less likely	5	−22.7	−45.8	10.5	0.209	.647
	More likely	17	−14.8	−31.0	5.2		
Assault on officer/officer injuries/resistance	Less likely	1	−22.5	−83.6	266.8	0.261	.610
	More likely	14	16.6	−4.6	42.6		
Use of force	Less likely	4	−7.3	−38.7	40.0	0.001	.981
	More likely	22	−6.8	−20.8	9.5		

*Note: k =* number of effect sizes; *Q*_Between_ is the test of the difference among/between the means with associated significance value *p*(*Q*_Between_).

#### 6.7.7 Compliance with treatment condition (fidelity)

Compliance with the treatment condition can come in various forms, from officers actually wearing BWCs when they are assigned to do so, to officers using them in the way that agency policy dictates. We coded studies as either having “higher” or “lower” levels of fidelity (as characterized by compliance to the treatment condition from what we could discern in an article or report). If we could not determine treatment fidelity for a study, we coded the study as “unsure.” As Table 4 showed, we were unsure about the level of fidelity to BWC implementation in 45% of the studies, even though in some of these studies we were able to discern the level of discretion that officers had in using their BWCs.

The findings from this moderator analysis are shown in Table 14, and we again caution the reader that any observed patterns may represent reporting artifacts by study authors rather than differential impacts from BWCs. Only one comparison is statistically significant: use of force.It seems that for use of force, there are clearer reductions when fidelity is high as compared to when it is low or uncertain (particularly the former). One lesson for researchers emerges from both this and the discretion moderator analysis above: researchers should try to more accurately document both official BWC policies regarding use and discretion as well as compliance with treatment conditions. Both would allow us to better discern whether these factors matter with regard to officer use of force.

**Table 14:** Mean percent change by intervention fidelity and construct

				95% CI			
Construct	Fidelity	*k*	Mean % change	Lower	Upper	*Q*_Between_	*p* (*Q*_Between_)
Arrests	Higher	2	4.2	−20.9	37.3	0.742	.690
	Lower	4	0.2	−18.1	22.6		
	Unsure	7	−6.9	−18.0	5.7		
Complaints against officer	Higher	4	−6.6	−34.3	32.6	3.474	.176
	Lower	8	−3.1	−28.1	30.7		
	Unsure	10	−32.2	−48.8	−10.3		
Assault on officer/officer injuries/resistance	Higher	4	33.3	−4.1	85.1	1.302	.522
	Lower	9	3.5	−22.2	37.6		
	Unsure	2	13.3	−72.5	366.5		
Use of force	Higher	5	−29.2	−46.4	−6.5	11.320	.003
	Lower	10	20.1	−1.4	46.2		
	Unsure	11	−17.1	−32.8	2.2		

*Note: k =* number of effect sizes; *Q*_Between_ is the test of the difference among/between the means with associated significance value *p*(*Q*_Between_).

#### 6.7.8 Researcher group

Finally, we examine the research organization behind the study as a possible moderating factor. In some cases, research groups may inadvertently influence intervention outcomes (see an example in Petrosino, Turpin‐Petrosino, & Guckenburg, 2014). BWC research has been dominated by researchers from two universities: The University of Cambridge (Barak Ariel and colleagues) and Arizona State University (ASU) (Michael White, Charles Katz, and colleagues). We compared mean RIRRs for use of force, complaints, arrests, and assaults on officers/resistance but did not find any significant differences between studies from these two universities or between them and other research groups (Table 15).

**Table 15:** Mean percent change by research team and construct

				95% CI			
Construct	Research Team	*k*	Mean % change	Lower	Upper	*Q*_Between_	*p* (*Q*_Between_)
Arrests	ASU	5	3.6	−14.6	25.7	0.814	.666
	Cambridge	1	−9.6	−34.0	23.9		
	Other	7	−5.8	−17.1	7.0		
Complaints against officer	ASU	4	−10.8	−37.5	27.3	0.683	.711
	Cambridge	9	−9.6	−40.2	36.5		
	Other	9	−23.5	−41.4	−0.2		
Assault on officer/officer injuries/resistance	ASU	2	16.5	−75.4	2727.9	0.974	.614
	Cambridge	10	9.6	−14.2	39.9		
	Other	3	30.3	−13.6	96.6		
Use of force	ASU	3	−0.5	−35.5	53.5	0.174	.916
	Cambridge	13	−6.3	−24.2	15.9		
	Other	10	−10.4	−31.2	16.7		

*Note: k =* number of effect sizes; *Q*_Between_ is the test of the difference among/between the means with associated significance value *p*(*Q*_Between_).

### 6.8 Publication bias

We strove to minimize publication and outcome selection bias through our search process that identified many unpublished works (e.g., technical reports, theses, etc.). We believe that it is unlikely that we missed unpublished eligible studies. Having access to a high proportion of unpublished reports also reduces, but does not eliminate, the possibility of differential outcome reporting based on the desirability of the findings. That said, roughly two‐thirds of the studies were only available in peer‐reviewed journal form (20 of 30). For nine studies, we had obtained a technical report or thesis, although all but three of these were also published as journal articles. One study was available only as a book publication.

Our analyses of publication selection bias focus on two outcomes: complaints and use of force. These two outcomes were the most commonly reported, both with over 20 effect sizes each, and both have a clear desirable or theoretically predicted direction of effect. That is, BWCs are expected to decrease both, whereas for many of the outcomes the expectation of direction of effect is less clear, making it more difficult to implement a trim‐and‐fill analysis that presumes censoring on the left or right side of the distribution. Comparing published studies (available in journal article or book only) to studies where we had access to a thesis or technical report shows that published‐only studies had a slightly more negative (desirable) average effect for use of force and a slightly less negative average effect for complaints. These differences between published‐only and other studies are not statistically significant.

**Figure 10:**
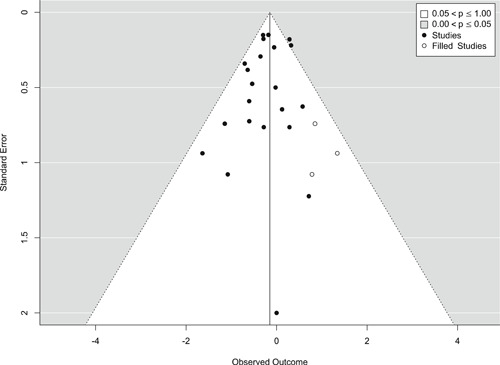
Funnel plot: complaints

**Figure 11:**
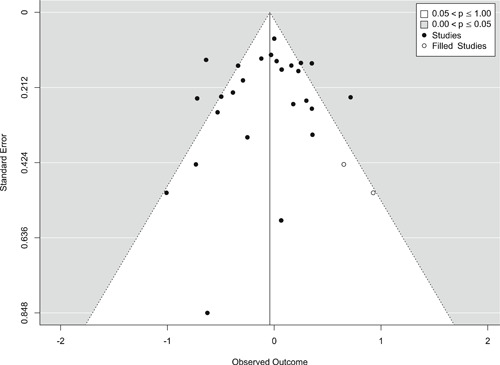
Funnel plot: use of force

The funnel plots for these two outcomes (Figures 10 and 11) are roughly symmetrical, which is what we would expect without publication selection bias. However, a trim‐and‐fill analysis detected a slight asymmetry suggesting three missing effect sizes to the right (positive values) of the mean for complaints and two missing effect sizes also to the right for use of force. The effect for complaints is slightly reduced (from −16.6 to −13.8). The use of force effect size is also reduced slightly, but it was initially small and nonsignificant. Egger's test for asymmetry was nonsignificant for both complaints (*p* = .28) and use of force (*p* = .22). Overall, these analyses suggest that any bias due to publication selection is likely to be small in magnitude. Furthermore, our overall results include a reasonable number of unpublished sources, reducing the potential seriousness of this threat. We are also fairly confident that we have identified all eligible studies in this area.

## 7 DISCUSSION

### 7.1 Summary of the results

Our meta‐analysis of 30 studies and 116 effects of police use of BWCs finds that this technology produces few clear or consistent impacts on police or citizen behaviors. Across a variety of outcome measures—including police use of force, complaints against officers, arrests, proactive police activities, assaults or resistance against officers, citizen calls for police service, and others—individual studies have produced a mix of positive, negative, and null findings. The average impact of BWCs on all of these outcomes but one is not statistically significant across studies. The one exception is with complaints—BWCs do seem to reduce complaints against police. The average relative reduction in complaints linked to BWCs is about 17% (and may be greater in agencies that have recent histories of more serious officer misconduct). It is unclear, however, to what extent this represents improvements in the behaviors of officers and citizens toward one another (and hence more positive interactions) or a decline in the willingness of citizens to file complaints against officers.

Additionally, the estimated effects of BWCs are quite variable (i.e., statistically heterogeneous) across studies, meaning that BWCs may increase these behaviors in some contexts and decrease them in others with considerable uncertainty about any typical effect across implementations. Our analysis examined several factors that may contribute to this variability, including whether randomized designs were used; the unit of assignment; susceptibility to contamination of treatment and control conditions; study fidelity; the level of discretion allowed by an agency's BWC policy; and an agency's recent history with reform initiatives or sentinel events. None of these factors were sufficient to explain the variability in BWC results, though there are tentative (albeit inconclusive) indications that BWCs are more effective in reducing police use of force (broadly defined) when agencies limit officer discretion in the use of the cameras. Further research is needed to better understand how these contextual factors and others—alone and in combination—influence the outcomes that police experience with BWCs, for better or worse.

Conclusions must also be tempered by limitations to the available evidence. Our analyses of use of force, complaints against police, assault/resistance against officers, and arrests are the most robust, as they are all based on 13 to 26 estimated effect sizes overall. These outcomes were more carefully studied in moderator analyses. Analyses of other outcome measures are based on eight or fewer estimated effect sizes overall and should be viewed more cautiously. Furthermore, some key moderator variables, such as an agency's BWC policy and officer compliance with BWC policies and protocols, have not been carefully described or measured in many or most BWC studies.

### 7.2 Overall completeness and applicability of evidence

We believe this systematic review captures the totality of the experimental and quasi‐experimental research on the impacts of BWCs on officer or citizen behaviors through September 2019. We also include the broadest range of outcomes collected, beyond only complaints and use of force, as collected in the Bureau of Justice Assistance's outcome directories (see White et al., 2019a, 2019b). The timeliness of this review (submitted to Campbell in April 2020) is reflected in the short time gap between its completion and the end date of the search (September 2019). Given the overall completeness and timeliness of this review, this study is applicable to current adoption and use of BWCs, which we discuss in greater detail in Section 8.1.

### 7.3 Quality of the evidence

Unlike research on other technologies within policing, a large proportion (over two‐thirds) of the BWC studies reviewed here used the “gold standard” of evaluation research—randomized controlled experiments. We did not find any evidence that study authors engaged in selective reporting of certain outcomes. However, a number of challenges remain within these experiments. For one, contamination and spillover of treatment and control conditions are present in most studies and are often unavoidable. As noted, many studies have tried to measure contamination, and our moderator analysis indicates that contamination could be obscuring the effects of BWCs, at least on arrests and assaults on officers. Another major concern is fidelity to treatment conditions, which could not be discerned from 45% of the studies.

### 7.4 Limitations and potential biases in the review process

Enough information was available in most studies to calculate effect sizes. In some cases, the review team requested additional data from study authors to facilitate effect size calculations. However, studies sometimes did not report key pieces of information that are regularly reported in experimental and quasi‐experimental research reports. Many BWC studies do not present detailed information about the agencies’ BWC policies, how closely officers adhered to the policies and study protocols, or the political or organizational context of BWC adoption. These omissions limited our ability to judge how findings might be influenced by an agency's background, BWC policy, and officers’ implementation of the policy in the field within the experimental or quasi‐experimental test.

Eligible studies also rely on officially reported measures (with the exception of Ready and Young, 2015). As we noted in Section 6.4, there may be risks of bias in the measurement of certain officially reported outcomes. Furthermore, the presumed direction of potential bias is toward the null, or bias against finding a positive effect of BWCs on specific outcomes. However, even this assumption needs further study, as emphasized by Braga et al. (2019).

Finally, there are challenges with the measures themselves in getting at some of the outcomes of interest to those who initially pushed for BWCs. Using the number of complaints against police or officer uses of force may not adequately measure police accountability or police–citizen relations, which were arguably the main concerns that prompted citizens and municipalities to push for the adoption of cameras in the first place. Studies using systematic social observations (both in‐person and also by observing videos) such as McCluskey et al. (2019), or police–citizen contact surveys, may provide more insights into these measurement challenges.

### 7.5 Agreements and disagreements with other studies or reviews

Overall, the mixed and null findings of this meta‐analysis reinforce the conclusions of Lum et al. (2019, p. 93) who stated the following based on a narrative review of the BWC literature:

Although officers and citizens are generally supportive of BWC use, BWCs have not had statistically significant or consistent effects on most measures of officer and citizen behavior or citizens’ views of police. Expectations and concerns surrounding BWCs among police leaders and citizens have not yet been realized by and large in the ways anticipated by each…BWCs will not be an easy panacea for improving police performance, accountability, and relationships with citizens. To maximize the positive impacts of BWCs, police and researchers will need to give more attention to the ways and contexts (organizational and community) in which BWCs are most beneficial or harmful.

Our finding that BWCs reduce complaints against officers is also consistent with inferences from Lum et al. (2019), though its interpretation continues to remain uncertain.

Our results are somewhat consistent with the hypothesis offered by various BWC experts (see Ariel et al., 2016a, 2016b, 2018; Braga et al., 2019; Gaub & White, 2020; White, 2019) that the effects of BWCs may vary across different contexts. This review shows that the effects of BWCs on a range of outcomes vary substantially, in both positive and negative ways, across different contexts. However, commonly suspected methodological and contextual moderators cannot completely account for this variation, if at all. Accordingly, there is still a need for further research and guidance on the uses of BWCs and contexts of BWC adoption that produce the most beneficial outcomes for police and communities—as well as those that can produce harmful outcomes that police should seek to avoid.

## 8 AUTHORS’ CONCLUSIONS

BWCs do not seem to impact officer or citizen behaviors—as measured by researchers—in the ways initially hoped for by either citizens or police leaders. The research indicates that the adoption of BWCs by police agencies will probably lead to a reduction in citizen complaints against officers. As Lum et al. (2019) hypothesize given the existing survey research on BWCs, this is likely due to a reduction in what officers feel are frivolous complaints and not due to significant changes in officer behaviors or in improvements in police–citizen relations. Additionally, the research does not seem to indicate that BWCs consistently impact officer use of force, assaults on, or resistance against police officers, arrest behaviors, officer‐initiated proactivity generally (or traffic and pedestrian stops more specifically), or dispatched calls for service. This review shows that the effects of BWCs on these outcomes vary substantially, in both positive and negative ways, across different contexts.

### 8.1 Implications for policing and communities

The majority of police agencies in the United States, and likely all of the police forces in the United Kingdom, have either already purchased BWCs or are about to purchase them. As discussed in Section 3, BWC acquisitions have been driven by community demands for police transparency and accountability (especially surrounding police use of force) and police agencies’ own interest in deterring misconduct among both their officers and citizens. Agencies may also have political motivations for camera acquisition beyond what we discuss here (see discussions by Nowacki & Willits, 2018; Smith, 2019). For example, irrespective of whether cameras impact levels of complaints or use of force, camera acquisition could signal to the community that agencies take citizens’ concerns about transparency seriously and that if an officer‐involved shooting occurs, an objective record will be captured. BWCs may also help victims of crime, as they seem to have evidentiary value for criminal cases, showing successes in increasing detection or clearance of crimes, or increasing the rate of guilty pleas (see Ellis et al., 2015; Goodall, 2007; Morrow et al., 2016; ODS Consulting, 2011; Owens et al., 2014).

At the same time, if law enforcement agencies continue to acquire BWCs, they and their communities should temper their expectations about them. This systematic review does not provide strong support that BWCs have the impacts on certain outcomes that were initially expected. This may be the result of the way researchers have measured outcomes, which might not capture these expectations very well. A decline in the volume of use of force in any agency does not necessarily indicate that accountability and transparency for use of force generally or any specific use of force has improved. Researchers have still yet to study whether BWCs impact the outcomes of internal or criminal investigations of police officers or the accountability infrastructures of police organizations. Reductions in the volume of citizen complaints do not necessarily speak to police–citizen relations, the extent to which community members see the police as legitimate, or the levels and types of disparity that may result from police actions. Declines in complaints may actually signal a weakening of accountability infrastructures. The police might define as “frivolous” some complaints that are, in aggregate, meaningful to community members, and the overall process of dealing with such complaints may reflect the strength of an agency's accountability system. Even if BWCs might save some agencies legal fees in not dealing with certain complaints as Braga, Barao, et al. (2018) and Braga, Sousa, et al. (2018) have found, such savings still do not speak to whether police–citizen relationships have been improved. These more substantive and organizational outcomes are likely what advocacy groups like Black Lives Matter and others are seeking with BWCs (see discussion by Lopez, 2017).

Similarly, some agencies and communities were worried that BWCs may cause the police to pull back on their proactive efforts, leading to possible increases in crime—that is, the “Ferguson Effect.” But the research reviewed here does not indicate that BWCs lead to lower officer proactivity generally, or traffic and pedestrian stops more specifically. But again, as pointed out by Lum et al. (2019), the important question is not whether proactivity increases or decreases, but what specific types of proactivity have increased or decreased and the impacts of those changes. Some proactive activities can be effective in reducing crime or improving citizen satisfaction, while other forms of proactivity may lead to community backlash and degrade police–citizen relationships. If BWCs have no impact on, for example, unconstitutional stop and searches, then this null effect may be viewed negatively. Further, recent research on the Ferguson effect indicates that other, more long‐standing factors cause officers to reduce their activity, in particular, low morale and cynicism (Marier & Fridell, 2020). A technology fix likely cannot undo or impact these deeper challenges in policing.

More generally, the broader research on police technology, which arguably predicted these findings (see Koper et al., 2014; Lum, Koper, & Willis, 2017), should not be ignored. The outcomes associated with police technologies are often a function of how agencies view and implement those technologies; as Manning (2008) points out, agencies shape technology use, not the other way around. Technologies are filtered through organizational and technological frames that shape their uses in specific ways (see Chan, Brereton, Legosz, & Doran, 2001; Orlikowski & Gash, 1994). Hopes for uses of BWCs that result in specific outcomes depend heavily on the abilities, infrastructures, and motivations of agencies. This idea bears out in the indication from our moderator analysis that reductions in use of force might be reaped if agencies highly restrict officer discretion in turning on and off cameras. Similarly, even if agencies have cameras to capture the most serious and rare sentinel events, it will not improve relations between police and citizens during those events if agencies do not have clear policies and practices for promptly releasing videos and handling internal investigations and disciplinary actions in ways that seem transparent, rigorous, and fair to the community (Lum & Nagin, 2017). An important question moving forward is whether and how police will use BWCs to strengthen their accountability systems.

We also encourage agencies (and their research partners) to expand their thinking about how cameras might be tested and used in other ways to achieve these goals. For example, as in the sports world, video playback can be used for mentorship, feedback, and every day in‐field training that ultimately can strengthen the agency's accountability to both the rule of law and to their various mandates of crime control and community legitimacy. Ultimately, the goal of police agencies is the prevention of sentinel events and bad behaviors in the first place, rather than paying for them later. Figuring out how to use cameras to reap long‐term gains of strengthening organizational accountability and functioning may be a better investment in camera use than the more short‐term gains measured here.

### 8.2 Implications for researchers

Researchers have provided the field with a great deal of knowledge about BWCs in less than a decade. The studies reviewed here are innovative and essential in advancing our knowledge of BWCs. Additionally, the broader BWC research—beyond outcome evaluations—provides important clues and contexts as to how BWCs work, how they are used, and the motivations behind their adoption. At the same time, this systematic review reveals many opportunities for researchers to expand and improve upon this area of research in a few key ways.

First, researchers can improve upon BWC studies by providing more details about several aspects of BWC use. One often missing piece of information is the context in which BWCs were adopted. We were only able to determine the history behind BWC adoption for five studies, but having this context for more studies may provide hints as to why certain results are found. Another often missing piece of information concerns the official policies that are in place for BWC use, in particular the specific types of discretion that officers have to use the cameras during their workdays and what information (if any) they need to provide to citizens about cameras. This would allow further testing in future meta‐analyses as to whether restrictive policies are needed to impact uses of force. Related to this is yet another request: that researchers more clearly differentiate elements of official policies from what actually happened during the implementation in the study itself. For example, officers may clearly adhere to treatment conditions by following agency policies precisely (high fidelity), but those policies may allow for a great deal of discretion with cameras. To examine this further, researchers might also replicate examples of studies that examine activation. For example, Katz and colleagues’ Phoenix studies examine activation explicitly using camera meta‐data (Hedberg et al., 2016; Katz et al., 2015, 2019). Yokum et al. (2019) also checked videos against calls for service to determine compliance with BWC policies. As another example, Henstock and Ariel (2017) used a police inspector to check treatment integrity on a daily basis and thus were able to provide this information with high confidence.

Second, finding ways to overcome, or at least measure, test, and control for contamination and spillover effects will advance this research area. This may be difficult to overcome in randomized controlled designs, although Bennett et al.'s (2019) study offers one possibility. In that study of the Fairfax County (Virginia) Police Department, officers were organized into squads who worked in the same locations but on different days (Squad “A” was assigned certain days, and Squad “B” was assigned other days to work). While there could be significant cultural differences between the two squads, such an approach, if there were enough squads that could be randomized, reduces the possibility of both within‐subject and across‐subject contamination. (However, citizen exposure to both BWC and non‐BWC conditions would still be a concern.) For studies where contamination is inevitable, more details about contamination effects can give readers a better understanding of the extent to which those effects occurred. Examples of studies that examine contamination explicitly by analyzing calls for service and multiple officer responses to calls are Braga, Barao, et al. (2018); Braga, Sousa, et al. (2018); Wallace et al. (2018); and Braga et al. (2019). Finally, Braga et al. (2019) and Ariel et al. (2017) also offer alternative ways to think about contamination effects. While contamination is seen as a problem in experimental research, both suggest that in the case of BWCs, contamination and spillover effects may lead to diffusion of benefits for the agency if officers (or the agency as a whole) are affected in positive ways by others wearing cameras. Further use of rigorous time series studies that examine changes after the complete or widespread adoption of BWCs would also be valuable in this regard and arguably has some advantages relative to the use of randomized experiments in studying this particular topic (see discussion by Chin‐Quee, 2018).

Third, researchers might also give more attention to the long‐term effects of BWC implementation. Time series studies with longer preintervention periods would be useful, but so would studies that examine long‐term compliance and activation, as well as the long‐term impacts of BWCs on the many outcomes discussed above. Perhaps once officers and citizens become used to cameras, effects might wear off (or become stronger). There may be unintended consequences of BWCs over long periods of time with regard to these outcomes.

A fourth, broader observation concerns the challenges of developing constructs that can measure BWC outcomes of interest. Almost all of the studies examined in this review use official measures of use of force, complaints, calls for service, and officer activity. Yet, for some, we hypothesize (although this remains to be tested) that the wearing of BWCs might influence the measurement of these outcomes. Reporting of the use of force, assaults against police officers, citizen complaints, and proactivity could all be influenced by camera wearing, thus biasing these measures. It is unclear what might be good alternatives for these measures. Systematic social observations like McCluskey et al. (2019) might be one alternative, but observational research cannot be sustained for long periods of time or across many officers. While developing more unbiased outcome measures may be a goal, it may not be realistic, given the outcomes available in policing research.

Most importantly, researchers might consider expanding their analysis to outcomes beyond those examined in this review. For example, finding ways to ascertain an agency's level of accountability or transparency to the public are important to understanding BWCs’ effects but are likely harder outcomes to measure. Understanding the impact that BWCs have on the reaction to and investigation of sentinel events such as officer‐involved shootings also is an important need to fill, especially since these events drove the adoption of BWCs in the first place. Finding ways to measure how BWCs impact police–citizen relationships or police agency legitimacy beyond the count of official complaints also seems especially needed, given that the number of complaints might not only reflect officer behavior but citizen reporting behavior. Another important question still yet to be examined is how BWCs might impact criminal justice disparities; determining what types of outcomes could be used to accurately gauge that effect would be a major advance in BWC research. Finally, as already mentioned, BWCs may prove to be an excellent tool for mentorship and everyday training. Determining how to measure whether BWCs strengthen first‐line supervision or officer accountability would contribute to this research area.

In summary, BWC research is a robust, rapidly growing, and responsive area of research. Researchers, practitioners, and funding agencies should be commended for developing this evidence‐base so quickly. This swift response is a model for new technologies that agencies and researchers might rapidly adopt in the future.

## ROLES AND RESPONSIBILITIES

C. L., C. S. K., and D. B. W. were responsible for writing the manuscript. C. L. and C. S. K. have primary responsibility in the organization and completion of this review, led the determination of eligible studies, the supplemental searches, the coding, and the organization of the review. D. B. W. led the meta‐analysis and statistical analysis for this review and developed the effect size calculations, as shown in the Supporting Information Appendices E and F. D. B. W. also guided the team in meta‐analysis and systematic review techniques and conventions. We are especially indebted to D. B. W. for his work on effect size calculations, given the unusual effects reported by BWC scholars. M. S. and M. G. served as the graduate research assistants on this review and contributed to the supplemental searches, eligibility determination, and coding of all study variables. E. E., A. H., and L. M. carried out the GPD search for this study, which was supplemented by additional searches by M. S. and M. G.

## SOURCES OF SUPPORT

Funding for this review comes from the Campbell Collaboration through support from the Laura and John Arnold Foundation. The views expressed in this report are those of the authors and do not necessarily reflect the views of the Laura and John Arnold Foundation or the Campbell Collaboration.

## CONFLICT OF INTERESTS

The authors declare that there are no conflict of interests. None of the authors are researchers on any evaluation study of BWCs examined in this review. None of the authors are, or have been, consultants, employees, or grant recipients for technology companies that produce and sell BWCs or related technologies, nor are they invested in government organizations supporting the use of BWCs.

## Supporting information

Supporting information

Supporting information

Supporting information

Supporting information

Supporting information

Supporting information

Supporting information

Supporting information

## Footnotes

1 See https://privacyinternational.org/sites/default/files/2019-02/Explainers-BWV.pdf.

2 See https://blacklivesmatter.com/.

3 Miller (2019) also documents the marked increase in the BWC market leader—Axon—after the investment of funds by the U.S. Department of Justice in 2015.

4 See https://civilrights.org/.

5 One of the most visible police leaders in the United States, James Comey, then Director of the Federal Bureau of Investigation, discussed this in a speech at the University of Chicago (see https://www.fbi.gov/news/speeches/law-enforcement-and-the-communities-we-serve-bending-the-lines-toward-safety-and-justice; see also Graham, 2015).

6 In White (2014), this is cited as a personal correspondence between White, Katz, and Kurtenbach (of the Phoenix Police Department).

7 We are specifically referring to crimes between citizens that the police are investigating (for example, domestic violence cases). There are no studies that we are aware of that evaluate the impact of BWCs on the criminal or internal investigation of officer misconduct. Nor are the studies on the impacts of cameras on criminal prosecutions easily interpreted in the context of internal investigations of the police or even criminal investigations of the police.

8 See http://www.gpd.uq.edu.au. The GPD search was part of the overall funding contract by Arnold Ventures to the Campbell Collaboration.

9 While the GPD data extends back to 1950, to date full‐text documents have only been screened back to 2003. For this systematic review, the authors believe the use of the GPD is justified, as the earliest recorded evaluation for BWCs according to Lum et al. was Goodall (2007), and the first experimental evaluation was Farrar (2012; as described in Ariel et al., 2015). Additionally, per the GPD search protocol, gray literature was also be searched (see Supporting Information Appendices A and B).

10 See https://www.bja.gov/bwc/Resources.html.

11 See https://www.libreoffice.org/.

12 See: https://osf.io/4t9wq/.

13 We debated whether to include Demir et al. (2020) in this review. This is a controlled quasi‐experimental study that examined the impact of BWCs on citizens’ perceptions of procedural justice. While we did not include studies focusing on perceptions, in this study, perceptions of officer behaviors were recorded soon after the actual encounter, thus supporting one argument that perceptions examined actual behaviors. Ultimately, this study was excluded.

14 The eligible pool consisted of 467 officers from six of the agency's seven precincts who gave preliminary consent to participate in a study of BWCs. Katz et al.'s analysis also included an additional group of officers that were randomly selected to wear BWCs but declined the request. Comparisons involving this group are not included in our review, as they were not wearing BWCs (a criteria for inclusion in this review).

15 In total, there were 11 studies presented in some of these reports, but after extensive conversations with the authors, one site was removed—“G”—because the authors said that it was an identified site in another study that appears in our review. However, the authors stated that no other site was one of the other studies that we had collected.

16 See: https://osf.io/4t9wq/.

17 During the course of our coding, we reached out to numerous study authors to ask for clarification on a wide variety of study aspects to clarify or enhance our coding. Almost all study authors replied to our inquiries.

18 We note that the Cochrane risk‐of‐bias tool was often difficult to apply to BWC research, and had a number of aspects that did not apply to this research area. For example, the tool's Domain 2, questions 2.1 and 2.2 ask about whether participants were aware of the assigned intervention. In this case, police officers wearing cameras were certainly aware they were wearing cameras, although citizens sometimes did or did not notice the cameras (even if officers alerted them to the cameras). It is also difficult to determine “deviations” from the intervention (2.3, 2.4, 2.5), as officers in some cases had discretion whether to turn on or off the cameras. Challenges with Domain 3 and 4 are also noted shortly.

19 The RIRR for citations is based on only two effect sizes and should be interpreted cautiously.

20 Some studies reported on even more categories (e.g., Lawrence & Peterson, 2019) but these three proactive activity categories were the most commonly reported across multiple studies.

21 We note that from the documents available about Rialto, there is no indication that Rialto was troubled, and both Farrar and Ariel (2013) and Ariel et al. (2015) describe the use of force and complaints in Rialto as “rare.” Nonetheless, White's hypothesis is reasonable, and we explore it here.
