# Supplementary material for: Body‐worn cameras’ effects on police officers and citizen behavior: A systematic review
Source: Campbell Syst Rev. 2020 Sep 9;16(3):e1112. doi: 10.1002/cl2.1112 (PMC8356344; doi:10.1002/cl2.1112)
Supplement: Supplementary file 1 — Supporting information [file CL2-16-e1112-s004.pdf]

APPENDIX A: GLOBAL POLICE DATABASE SYSTEMATIC SEARCH STRATEGY

Part of: Lum, C., Koper, C.S., Wilson, D.B., …et al. (2020). Body-worn cameras' effects on police officers and citizen behavior: A systematic review. Campbell Systematic Reviews 2020;e1112. https://doi.org/10.1002/cl2.1112.

Search Terms

To ensure optimum sensitivity and specificity, the GPD search strategy utilises a combination of free-text and controlled vocabulary search terms. Because controlled vocabularies and search capabilities vary across databases, the exact combination of search terms and field codes are adapted to each database. Final search syntax for each location will be reported in the final review.

The free-text search terms for the GPD are provided in Table 1 and are grouped by substantive (i.e., some form of policing) and evaluation terminology. Although the search strategy across search locations may vary slightly, the search follows a number of general rules:

- Search terms will be combined into search strings using Boolean operators "AND" and "OR". Specifically, terms within each category will be combined with "OR" and categories will be combined with "AND". For example: (police OR policing OR "law#enforcement") AND (analy* OR ANCOVA OR ANOVA OR …).
- Compound terms (e.g., law enforcement) will be considered single terms in search strings by using quotation marks (i.e., "law*enforcement") to ensure that the database searches for the entire term rather than separate words.
- Wild cards and truncation codes will be used for search terms with multiple iterations from a stem word (e.g., evaluation, evaluate) or spelling variations (e.g., evaluat* or randomi#e).
- If a database has a controlled vocabulary term that is equivalent to "POLICE", we will combine the term in a search string that includes both the policing and evaluation free-text search terms. This approach will ensure that we retrieve documents that do not use policing terms in the title/abstract but have been indexed as being related to policing in the database. An example of this approach is the following search string: (((SU: "POLICE") OR (TI,AB,KW: police OR policing OR "law*enforcement")) AND (TI,AB,KW: intervention* OR evaluat* OR compar* OR …)).
- For search locations with limited search functionality, we will implement a broad search that uses only the policing free-text terms.
- Multidisciplinary database searches will be limited to relevant disciplines (e.g., include social sciences but exclude physical sciences).
- Search results will be refined to exclude specific types of documents that are not suitable for systematic reviews (e.g., newspapers, front/back matter, book reviews).

Table 1. Free-text search terms for the GPD systematic search

Policing Search Terms	Evaluation Search Terms			
police<br>policing<br>"law*enforcement"<br>constab*<br>detective*<br>sheriff*	analy*<br>ANCOVA<br>ANOVA<br>"ABAB design"<br>"AB design"<br>baseline<br>causa*<br>"chi#square"<br>coefficient*<br>"comparison condition*"<br>"comparison group*"<br>"control condition*"<br>"control group*"<br>correlat*<br>covariat*<br>"cross#section*"	data<br>effect*<br>efficacy<br>eval*<br>experiment*<br>hypothes*<br>impact*<br>intervent*<br>interview*<br>longitudinal<br>MANCOVA<br>MANOVA<br>"matched group"<br>measure*<br>"meta-analy*"<br>"odds#ratio*	outcome*<br>paramet*<br>"post-test"<br>posttest<br>"post test"<br>predict*<br>"pre-test"<br>pretest<br>program*<br>"propensity score*"<br>quantitative<br>"quasi#experiment*"<br>questionnaire*<br>random*<br>RCT<br>regress*	result*<br>"risk#ratio*"<br>sampl*<br>"standard deviation*"<br>statistic*<br>studies<br>study<br>survey*<br>"systematic review*"<br>"t#test*"<br>"time#series"<br>treatment*<br>variable*<br>variance

Search Locations

To reduce publication and discipline bias, the GPD search strategy adopts an international scope and involves searching for literature across a number of disciplines (e.g., criminology, law, political science, public health, sociology, social science and social work). The search captures a comprehensive range of published (i.e., journal articles, book chapters, books) and unpublished literature (e.g., working papers, governmental reports, technical reports, conference proceedings, dissertations) by implementing a search strategy across bibliographic/academic, grey literature, and dissertation databases or repositories.

We note that there is substantial overlap of the content coverage between many of the databases. Therefore, we have used the *Optimal Searching of Indexing Databases* (OSID) computer program (Neville & Higginson, 2014) to analyse the content crossover for all databases that have accessible content coverage lists. OSID analyses the content coverage and creates a search location solution that provides the most comprehensive coverage via the least number of databases. For example, if the content for the set of databases seen in Figure 1 were imported, OSID would provide a solution that entails searching only databases 3 and 4 because the content covered by databases 1 and 2 is covered by database 4. Another advantage of using OSID when designing a search strategy is the reduction in the number of duplicates that would need to be removed prior to the screening phase. Databases with >10 unique titles were be searched in full, whereas for databases with ≤10 unique titles, we searched only the unique titles and any non-serial content (e.g., reports, conference proceedings). Where a modified search of a database would be more labour intensive than a full search and export results, conducted a full search of the database. The final search locations and solution are reported in Table 2.

Table 2. GPD search locations and protocol (January 1st 1950 – December 2018)

INDEXED & ACADEMIC DATABASES		CONTENT COVERAGE FED INTO OSID?	FULL OR MODIFIED SEARCH?	SEARCH MODIFICATIONS
**ProQuest**	Criminal Justice	Yes	Full	None.
	Dissertation and Theses Database Global	Not Available	Modified	Social Sciences subset.
	Political Science	Yes	Full	None.
	Periodical Archive Online	Yes	Full	None.
	Research Library	Yes	Modified	Social Sciences subset.
	Social Science Journals	Yes	Full	None.
	Sociology	Yes	Modified	Search 2 unique journal titles and non-serial content only.
	Applied Social Sciences Index and Abstracts	Yes	Full	None.
	International Bibliography of the Social Sciences	Yes	Full	None.
	Public Affairs Information Service	Yes	Full	None.
	Social Services Abstracts	Yes	Modified	Search 5 unique journal titles and non-serial content only.
	Sociological Abstracts	Yes	Full	None.
	Worldwide Political Sciences Abstracts	Yes	Modified	Search 9 unique journal titles and non-serial content only.
**EBSCO**	Academic Search Premier	Yes	Full	None.
	Criminal Justice Abstracts	Yes	Full	None.
	EconLit	Yes	Full	None.
	MEDLINE with Full-Text	Yes	Full	None.
	Social Sciences Full-Text	Yes	Full	None.
**OVID**	International Political Science Abstracts	Not Available	Full	None.
	PsycARTICLES	Yes	Modified	Search 4 unique journal titles only.

	PsycEXTRA	Not Available	Full	None.
	PsycINFO	Yes	Full	None.
	Social Work Abstracts	Not Available	Full	None.
**Web of Science**	Current Contents Connect – Social and Behavioural Sciences Edition	Yes	Modified	Search 1 unique journal title and non-serial content only.
	Book Citation Index (Social Sciences and Humanities)	Not Available	Full	None.
	Conference Proceedings Citation Index (Social Sciences and Humanities)	Not Available	Full	None.
	Social Science Citation Index	Yes	Full	None.
**Informit**	Australian Attorney General Information Service	Yes	Full	None.
	Australian Criminology Database (CINCH)	Yes	Full	None.
	Australian Federal Police Database	Yes	Full	None.
	Australian Public Affairs Full-Text	Yes	Full	None.
	DRUG	Yes	Full	None.
	Health & Society Database	Yes	Modified	Search unique journal titles and non-serial content only.
	Humanities and Social Sciences Collection	Yes	Full	None.
**Gale-Cengage**	Expanded Academic ASAP	Yes	Full	None.
**STANDALONE & OPEN ACCESS DATABASES**	Cambridge Journals Online	Yes	Modified	Search 4 unique journal titles in Law and Political Science collections and full search of Social Studies collection.
	Directory of Open Access Journals	Yes	Full	None.
	HeinOnline	Yes	Modified	Law Journals Online collection only.
	JSTOR	Yes	Modified	Search unique titles across the Law, Political Science, Public Health, Public Policy, Social Work and Sociology collections only. The Criminal Justice collection had no unique content and so will be excluded from the search. Only 10% of content in this database have abstracts and a full-text search returns >250,000 results because of inability to construct complex search strings. Therefore, a modified search of the unique titles

				across these collections will be more pragmatic than a full search of the database.
	Oxford Scholarship Online	Yes	Full	None.
	Sage Journals Online and Archive (Sage Premier)	Yes	Modified	Search 5 unique journal titles and non-serial content only.
	ScienceDirect	Yes	Full	None.
	SCOPUS	Yes	Full	None.
	SpringerLink	Yes	Full	Although this database has low uniqueness when combined with the full set of databases, a full search using only the policing search terms will be more pragmatic than a modified search on unique titles because of the restricted search functionality of this database.
	Taylor & Francis Online	Yes	Modified	Although this database has low uniqueness when combined with the full set of databases, a full search using only the policing search terms will be more pragmatic than a modified search on unique titles because of the restricted search functionality of this database.
	Wiley Online Library	Yes	Full	None.
	California Commission on Peace Officer Standards & Training Library	No	Full	None.
	Cochrane Library	No	Full	None.
	CrimeSolutions.gov	No	Full	None.
	Database of Abstracts of Reviews of Effectiveness (DARE)	No	Full	None.
	FBI – The Fault (Reports and Publications)	No	Full	None.
	Evidence-Based Policing Matrix	No	Full	None.
	International Initiative for Impact Evaluation Database (3ie)	No	Full	None.
	National Criminal Justice Reference Service	No	Full	None.
	Safety Lit Database	No	Full	None.
	Australian Institute of Criminology	No	Full	None.
	Bureau of Police Research and Development (India)	No	Full	None.

	Canadian Police Research Catalogue	No	Full	None.
	Centre for Problem-Oriented Policing	No	Full	None.
	College of Policing (including POLKA and Crime Reduction Toolkit)	No	Full	None.
	European Police College (CEPOL)	No	Full	None.
	Evidence for Policy and Practice Information and Coordinating Centre	No	Full	None.
	National Research Institute of Police Science (Japanese)	No	Full	None.
	Office of Community Oriented Policing Services	No	Full	None.
	Police Executive Research Forum (US)	No	Full	None.
	Police Foundation (US)	No	Full	None.
	Tasmania Institute of Law Enforcement Studies (Australia)	No	Full	None.
	Policing Online Information System (POLIS, Europe)	No	Full	None.
	Scottish Institute for Policing Research	No	Full	None.
	Centre of Excellence in Policing and Security (Australian, now archived)	No	Full	None.

APPENDIX B: GLOBAL POLICE DATABASE SYSTEMATIC COMPILATION STRATEGY

Inclusion Criteria

Each record captured by the GPD systematic search must satisfy all inclusion criteria to be included in the GPD: timeframe, intervention and research design. There are no restrictions applied to the types of outcomes, participants, settings or languages considered eligible for inclusion in the GPD.

Types of interventions

Each document must contain an impact evaluation of a policing intervention. We define a policing intervention is some kind of a strategy, program, technique, approach, activity, campaign, training, directive, or funding / organisational change that involves police in some way (other agencies or organisations can be involved). Police involvement is broadly defined as:

- Police initiation, development or leadership
- Police are recipients of the intervention or the intervention is related, focused or targeted to police practices
- Delivery or implementation of the intervention by police

Types of study designs

The GPD includes quantitative impact evaluations of policing interventions that utilise randomised experimental (e.g., RCTs) or quasi-experimental evaluation designs with a valid comparison group that does not receive the intervention. The GPD includes designs where the comparison group receives 'business-as-usual' policing, no intervention or an alternative intervention (treatment-treatment designs).

The specific list of research designs included in the GPD are as follows:

- Systematic reviews with or without meta-analyses
- Cross-over designs
- Cost-benefit analyses
- Regression discontinuity designs
- Designs using multivariate controls (e.g., multiple regression)
- Matched control group designs with or without pre-intervention baseline measures (propensity or statistically matched)
- Unmatched control group designs with pre-post intervention measures which allow for difference-in-difference analysis
- Unmatched control group designs without pre-intervention measures where the control group has face validity
- Short interrupted time-series designs with control group (less than 25 pre- and 25 post-intervention observations (Glass, 1997))
- Long interrupted time-series designs with or without a control group (≥25 pre- and post-intervention observations (Glass, 1997))
- Raw unadjusted correlational designs where the variation in the level of the intervention is compared to the variation in the level of the outcome

The GPD excludes single group designs with pre- and post-intervention measures as these designs are highly subject to bias and threats to internal validity.

Systematic Screening

To establish eligibility, records captured by the GPD search are progress through a series of systematic stages which are summarised in Figure 1, with additional detail provided in the following subsections.

All research staff working on the GPD undergo standardised training before beginning work within any of the stages detailed below. Staff then complete short training simulations to enable an assessment of their understanding of the GPD protocols and highlight any areas for additional training. In addition, random samples of each staff's work are regularly cross-checked to ensure adherence to protocols. Disagreements between staff are mediated by either the project manager or GPD chief investigators.

Title and abstract screening

After removing duplicates, the title and abstract of record captured by the GPD systematic search is screened by trained research staff to identify potentially eligible research that satisfy the following criteria:

- Document is dated between 1950 – present
- Document is unique (i.e., not a duplicate)
- Document is about police or policing
- Document is an eligible document type (e.g., not a book review)

Records are excluded if the answer to any one of the criteria is unambiguously 'No', and will be classified as potentially eligible otherwise. Records classified as potentially eligible progress to full-text document retrieval and screening stages.

Full-text eligibility screening

Wherever possible, a full-text electronic version of eligible records will be imported into SysReview. For records without an electronic version, a hardcopy of the record will be located to enable full-text eligibility screening. The full-text of each document will be screened to identify studies that satisfy the following criteria:

- Document is dated between 1950 – present;
- Document is unique;
- Document reports a quantitative statistical comparison;
- Document reports on policing evaluation;
- Document reports in a quantitative impact evaluation of a policing intervention; and
- Evaluation uses an eligible research design.

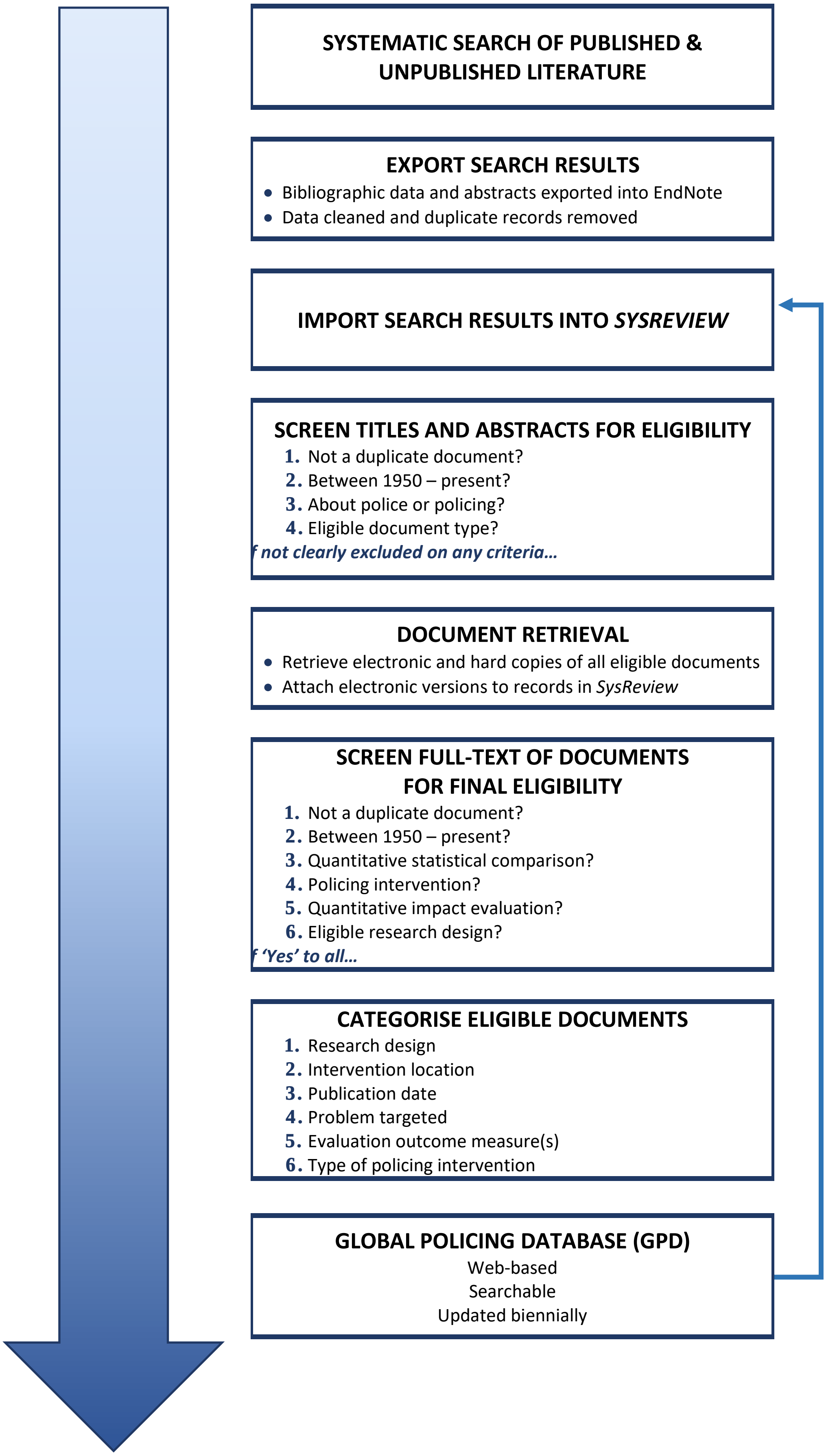

SYSTEMATIC SEARCH OF PUBLISHED & UNPUBLISHED LITERATURE
EXPORT SEARCH RESULTS
Bibliographic data and abstracts exported into EndNote
Data cleaned and duplicate records removed
IMPORT SEARCH RESULTS INTO *SYSREVIEW*
SCREEN TITLES AND ABSTRACTS FOR ELIGIBILITY
1. Not a duplicate document?
2. Between 1950 – present?
3. About police or policing?
4. Eligible document type?
f not clearly excluded on any criteria…
DOCUMENT RETRIEVAL
Retrieve electronic and hard copies of all eligible documents
Attach electronic versions to records in *SysReview*
SCREEN FULL-TEXT OF DOCUMENTS FOR FINAL ELIGIBILITY
1. Not a duplicate document?
2. Between 1950 – present?
3. Quantitative statistical comparison?
4. Policing intervention?
5. Quantitative impact evaluation?
6. Eligible research design?
f 'Yes' to all…
CATEGORISE ELIGIBLE DOCUMENTS
1. Research design
2. Intervention location
3. Publication date
4. Problem targeted
5. Evaluation outcome measure(s)
6. Type of policing intervention
GLOBAL POLICING DATABASE (GPD)
Web-based
Searchable
Updated biennially
