# Supplementary material for: Body‐worn cameras’ effects on police officers and citizen behavior: A systematic review
Source: Campbell Syst Rev. 2020 Sep 9;16(3):e1112. doi: 10.1002/cl2.1112 (PMC8356344; doi:10.1002/cl2.1112)
Supplement: Supplementary file 2 — Supporting information [file CL2-16-e1112-s006.pdf]

## APPENDIX C: LIST OF BWC SCHOLARS AND EXPERTS CONTACTED DURING THE SEARCH PROCESS

*Part of: Lum, C., Koper, C.S., Wilson, D.B., …et al. (2020). Body-worn cameras' effects on police officers and citizen behavior: A systematic review. Campbell Systematic Reviews 2020;e1112. https://doi.org/10.1002/cl2.1112.*

Ian Adams, University of Utah
Barak Ariel, University of Cambridge
Brad Bartholomew, American University
Richard Bennett, American University
Anthony Braga, Northeastern University
David Choate, Arizona State University
James Coldren, C.N.A. Corporation
Alexander Coppock, Yale University
Matthew Crow, University of West Florida
Scott Culhane, University of Wyoming
Stewart D'Alessio, Florida International University
Tom Ellis, University of Portsmouth
William Farrar, Mt. San Jacinto College
Jamie Flexon, Florida International University
Lorie Fridell, University of South Florida
Janne Gaub, East Carolina University
Martin Goodall, Keystone Law
Sean Goodison, Police Executive Research Forum
Joseph Gramaglia, Buffalo, NY Police Department
Rob Guerette, Florida International University
Paul Guerin, University of New Mexico
Elliot Harkavy, C.N.A. Corporation
Andrea Headley, Ohio State University
Eric Hedberg, National Opinion Research Center
Darren Henstock, Western Australia Police
Jessica Huff, Arizona State University
Jordan Hyatt, Drexel University
Shelley Hyland, Bureau of Justice Statistics
Wesley Jennings, University of Mississippi
Robert Kaminski, University of South Carolina
Charles Katz, Arizona State University
Erin Kerrison, University of California, Berkeley
Marthinus Koen, University of Southern Indiana
Wendy Koslicki, Ball State University
Michael Kyle, Southern Illinois University
Nancy La Vigne, Urban Institute
Daniel Lawrence, Urban Institute
David Makin, Washington State University

John Markovic, USDOJ COPS Office
Jon Maskaly, University of Texas, Dallas
Sharon Mastracci, University of Utah
Linda Merola, George Mason University
David McClure, Police Executive Research Forum
John McCluskey, Rochester Institute of Technology
Jack McDevitt, Northeastern University
Kyle McLean, Florida State University
Renee Mitchell, RTI International
Weston Morrow, University of Nevada, Reno
Bryce Newell, University of Kentucky
Peter Neyroud, University of Cambridge
Jeffrey Nowacki, Colorado State University
Jonah Obasi, Walden University
Catherine Owens, College of Policing
Megan Parry, University of Rhode Island
Jon Peha, Carnegie Mellon University
William Pelfrey, Virginia Commonwealth University
Bryce Peterson, Urban Institute
Joshua Phelps, Norwegian Police University College, Oslo
Justin Ready, Griffith University
Denise Rodriguez, C.N.A. Corporation
Mike Rose, University of California, Los Angeles
Mike Rowe, University of Liverpool
Auzeen Shariati, St. Joseph's College
John Smykla, Florida Atlantic University
William Sousa, University of Nevada, Las Vegas
Lisa Stolzenberg, Florida International University
Alex Sutherland, The Behavioral Insights Team
Justice Tankebe, Cambridge University
Emmeline Taylor, City, University of London
Natalie Todak, The University of Alabama at Birmingham
Paige Thompson, Urban Institute
Craig Uchida, Justice & Security Strategies, Inc.
Danielle Wallace, Arizona State University
Brandon Welsh, Northeastern University
Michael White, Arizona State University
James Willis, George Mason University
Dale Willits, Washington State University
Scott Wolfe, Michigan State University
Rob Worden, John F. Finn Institute for Public Safety
David Yokum, Brown University
Jacob Young, Arizona State University
Greg Zimmerman, Northeastern University
