# Supplementary material for: Body‐worn cameras’ effects on police officers and citizen behavior: A systematic review
Source: Campbell Syst Rev. 2020 Sep 9;16(3):e1112. doi: 10.1002/cl2.1112 (PMC8356344; doi:10.1002/cl2.1112)
Supplement: Supplementary file 3 — Supporting information [file CL2-16-e1112-s005.pdf]

APPENDIX D: CODING INSTRUMENT

Part of: Lum, C., Koper, C.S., Wilson, D.B., …et al. (2020). Body-worn cameras' effects on police officers and citizen behavior: A systematic review. Campbell Systematic Reviews 2020;e1112. https://doi.org/10.1002/cl2.1112.

This study employed three levels of coding: study, outcome, and effect size. The data collected for each level is detailed below. Please note that the coding instrument in the Protocol was a “living document”, and is modified here in terms of ordering, grouping, and expanded explanations and questions. The listing and names of variables follows the coding sheets that were used in LibreOffice.

STUDY LEVEL CODING

Identification information

1. Study ID. Denotes independent studies. StudyIDs begin with “S” followed by a three-digit number that is one of the record numbers of one of the documents associated with this study (e.g., S600).
2. SubStudy ID. Denotes special cases, as described in detail above in Section 5.3, “Determination of independent studies.” For the ten anonymous Ariel global studies, the SubStudy ID corresponds to the Site Letter that Ariel et al. uses. For the Katz et al. (2019) Phoenix, Arizona non-Maryvale study, the SubStudy ID refers to two separate studies: “A” Randomized Controlled Experiment and “B” Quasi Experiment. For the White et al. (2018) and Wallace et al. (2018) studies, the SubStudy ID separates White et al. (A) from Wallace et al. (B).
3. Author & Year. Unique label created to identify each independent study (except in the case of the Spokane study where it identifies the two separate articles) that is used in the display of results. (e.g., “Ariel (2016, 2017) DENVER, CO” or “Braga et al. (2019) BOSTON, MA”). See corresponding Table X in the review for more information.
4. Coder's Initials. The initials of the coder who examined this study. Note: for reconciliations, the third party was not listed.
5. Date Modified. Auto-generated date and timestamp for the last time any changes were made to this record.
6. ReferenceID. The initial number assigned to the abstract. This begins with “R” followed by a three digit number (e.g., R600).
7. Reference. The full reference, in APA format, associated with the Reference ID.

Descriptive features of studies

8. Treatment description. Text box that indicates the treatment condition (i.e. “Officers wearing BWCs”).
9. Control description. Text box that indicates the control condition (i.e. “Officers not wearing BWCs”).
10. Location name. Specific location or jurisdiction in which the study was conducted (i.e., “Spokane” or “Boston”).
11. State or Province. Broader state or province of the location (i.e., “Washington” or “Massachusetts”).
12. Country. Country that houses the location (i.e., “USA,” “UK,” or “Uruguay”).
13. Agency name. Law enforcement agency name if known (i.e., “Boston Police Department”).
14. Primary organization in which the study was conducted. Denotes usually the university (i.e., “Arizona State University”) or organization (i.e., “Urban Institute) under the auspices by which the study was conducted).

    a. Agency type.
    b. Law enforcement only.
    c. Law enforcement agency with correctional duties (e.g., Sheriff's agencies).
    d. Regional agency that oversees multiple jurisdictions, or a national agency
15. Number of sworn officers. Number of full-time sworn officers in the agency in the year in which the intervention was initiated. While some studies provided estimates, in cases where precise numbers were not provided, statistics for the closest available year to the intervention year were obtained from the Bureau of Justice of Statistics Census of State and Local Law Enforcement Agencies, the Law Enforcement Management and Statistics Survey or the Uniformed Crime Reports.
16. Number of officers involved in the study.
17. Intervention start date (mm/dd/year). The exact date, if known, when the study intervention began.
18. Intervention end date (mm/dd/year). The exact date, if known, when the study intervention ended.
19. Data collection start date (mm/dd/year). For example, studies may collect data that are dated much earlier than the start date of the intervention, to develop pre-intervention comparison periods.
20. Data collection end date (mm/dd/year).
21. Year of initial implementation of BWCs. If known, this is the year that BWCs were first implemented by the agency. In some cases, study authors might not indicate whether the year was the implementation or the acquisition year.
22. In the two years prior to camera adoption, had this agency or jurisdiction undergone a collaborative reform, consent decree, USDOJ review, or another official review due to sentinel events?
    a. Yes, mentioned in the article.
    b. No, or not mentioned in the article.

BWC Features

23. Were BWCs used in the agency prior to the study?
    a. BWCs were already in use by agency before the study began across the agency (not selective use).
    b. BWCs were already in use by agency before the study began, but only by specific and very limited units/people or in a pilot testing phase.
    c. Use of BWCs began very close to the time of the study or for the purposes of the study.
    d. Unknown.
24. Nature of BWC use during the intervention?
    a. Uniformed patrol only.
    b. Specialized units only (traffic, investigative, community-oriented, etc.).
    c. Combination uniformed patrol and specialized units.
    d. Training environments (academy, in-service).
    e. Other [text box]
25. BWC is turned on by default for the entire shift or is turned on for any police encounter (e.g., turned on when an officer leaves her vehicle)? This is about the official policy related to BWCs and how they were to be operated by officers. This may not fully capture the level of discretion officers have within that policy (Question 26, below) or the compliance with the treatment condition (Questions 28 and 29, below).
    a. Yes
    b. No
    c. Cannot tell

26. Discretion regarding on/off decision?[1] This question refers to an agency's official policies or mandates regarding the use of the BWCs. In some cases, policies already existed, and in other cases, rules were created from the study treatment conditions.
    a. No or low discretion (as specifically described in policy or police agency directives; default is on except in very specific circumstances).
    b. Moderate discretion (guidance regarding circumstances for discretion are given in policy or policy agency directive).
    c. Complete discretion (policies or police agency directives leave it up to individual officers to decide).
    d. Cannot tell.
27. Must inform citizens that BWC is on? (as dictated by policy or agency directive).
    a. Yes
    b. No
    c. Not specified
28. Fidelity: Describe any concerns regarding fidelity of the intervention.
29. Level of compliance with BWC condition. This is a very specific fidelity concern that was collected for the moderator analysis.
    a. Higher
    b. Lower
    c. Unsure

Design Features

30. How the sample was selected?
    a. All available units of analysis were used.
    b. Convenience sampling: only those who volunteered were used.
    c. Specific sample: Agency only wanted specific units to use BWCs and/or be involved in the study.
    d. Hybrid – sample is selected partly from convenience and partly from random selection.
31. Research design?
    a. RCT (randomized control trial): simple
    b. RCT: block randomized
    c. RCT: cluster randomized
    d. QE (quasi-experiment): simple matching
    e. QE: propensity score
    f. QE: statistical adjustment for baseline
    g. QE: historical/cohort design
    h. QE: time series
    i. QE: other
32. BWC use phased in during study period?
    a. Yes
    b. No
    c. Cannot tell
33. Sample sizes at start of study (e.g., at the point of randomization)
    a. Treatment Group sample size: [number]
    b. Control Group sample size: [number]
34. Unit of allocation (random assignment unit)
    a. Officer
    b. Shift

[1] In the initial Protocol, Question 26 and 27 were a single question on activation requirements. However, to clarify that question better, two questions were preferred.

    c. Officer-shift combination
    d. Enforcement groups (squads, specialized units)
    e. Police-defined geographic areas (beats, districts, sectors, etc.)
    f. Other geographic areas (hot spots, census blocks, etc.)
    g. Other [text box]

35. Did the author(s) report an a priori power analysis?
    a. Yes (explicitly stated)
    b. No (no mention or post hoc)
36. Description of sample selection bias concerns. [text box]
37. Attrition concerns described. [text box]
38. Contamination concerns. [text box]
39. Dichotomous coding of contamination concerns.
    a. More likely
    b. Less likely

Miscellaneous

40. Publication Types (check all that apply [multiple documents may be coded as part of one study]):
    a. Journal
    b. Book
    c. Book Chapter
    d. Technical report (government agency)
    e. Technical report (university/research institute)
    f. Dissertation/thesis
    g. Presentation
    h. Other
41. Population density [number]. Calculated as close to the year of BWC implementation as possible using data taken from the U.S. census. For anonymous sites, author reporting of population is used.
42. Diversity index [number]. To calculate measures of racial and ethnic diversity/heterogeneity, we used the diversity index developed by Meyer & McIntosh (1991). This index determines the probability/likelihood that two people chosen at random from a population will be different ethnicities. The index ranges from 0-1, with a value of zero indicating no difference in diversity in a population, and 1 indicating complete differences of diversity in a population. In 2000 and 2010, due to changes in categorization by the U.S. Census, we used the following formula: Diversity index = 1- [(percent white^2 + percent black^2 + percent Native American^2 + percent Asian^2 + percent Pacific Islander^2) * (percent non-Hispanic^2 + percent Hispanic^2)].
43. Additional notes.

Cochrane Risk-of-Bias Tool

(Y=Yes; PY=Probably Yes; PN=Probably No; N=No; NI=No information)

Domain 1: Risk of bias arising from the randomization process

44. (1.1) Was the allocation sequence random? (Y/PY/PN/N/NI)
45. (1.3) Did baseline differences between intervention groups suggest a problem with the randomization [or other selection] process? (Y/PY/PN/N/NI)
46. Optional: Were there violations to the randomization process? For example, did the officers who were assigned to wear BWCs refuse to wear them or did the officers assigned to the control condition/group wear BWCs anyway (e.g., they refused to give up BWCs that they were already using)? (Y/PY/PN/N/NI)

47. Was there contamination between the treatment and control conditions? For example, were there incidents in which a control officer responded to an incident/call for service, but a treatment officer provided backup so both were present on the scene simultaneously? Alternatively, were the same officers part of both the treatment and control conditions but assigned to one or the other based on different shifts? (Y/PY/PN/N/NI)
48. Optional: What is the predicted direction of bias arising from the randomization [or other selection] process? This includes selection bias and contamination bias.
    a. NA (no evidence bias)
    b. Towards the null
    c. Away from the null
    d. Unpredictable

Domain 2: Risk of bias due to deviations from the intended interventions (effect of assignment to intervention)

49. (2.6) Was an appropriate analysis used to estimate the effect of assignment to intervention? (Y/PY/PN/N/NI)
50. (2.7) Was there potential for a substantial impact (on the result) of the failure to analyze participants in the group to which they were randomized? (Y/PY/PN/N/NI)

Domain 5: Risk of bias in selection of the reported result

51. (5.1) Were the data that produced this result [the results for this study] analyzed in accordance with a pre-specified analysis plan that was finalized before unblinded outcome data were available for analysis? (Y/PY/PN/N/NI)
52. (5.2) Is (Are) the numerical result [results] being assessed likely to have been selected, on the basis of the results, from multiple outcome measurements (e.g. scales, definitions, time points) within the outcome domain? (Y/PY/PN/N/NI)
53. (5.3) Is (Are) the numerical result [results] being assessed likely to have been selected, on the basis of the results, from multiple analyses of the data? (Y/PY/PN/N/NI)

<u>OUTCOME-LEVEL CODING</u>

Each eligible dependent variable or measure will be coded as a separate record. The Primary Key that is the unique identifier for each record in the outcome table is the combination of the following fields: StudyID, SubStudyID, OutcomeID, and CoderID. The StudyID and SubStudyID.

1. Study ID & Substudy ID. As described above.
2. Author & Year. As described above.
3. Outcome ID and Outcome Label. Each study may have multiple outcome. Each outcome and its associated effect sizes were coded separately. The Outcome ID and its associated labels are:
    a. 1=Complaints
    b. 2=Use of force
    c. 3=Officer-initiated calls for service (unless only specified Stop and Frisk or Field Interrogation, then see (8) below)
    d. 4=Arrests
    e. 5=Citations (ordinance, not traffic citations)
    f. 6=Dispatched calls for service
    g. 7=Incident reports
    h. 8=Stop and frisk or field interrogation stops

    i. 9=Warnings (not related to traffic)
    j. 10=Assault on officers/officer injuries
    k. 11=Resistance against officers
    l. 12=Response time
    m. 13=Time on scene
    n. 14=Traffic stops or tickets
4. Coder's Initials. As described above.
5. Date Modified. As described above.
6. Construct measured by this outcome. This corresponds to the Outcome label as described above and are grouped by officer behavior and citizen behavior.
   Officer Behavior
    a. Complaints against officer
    b. Use of force
    c. Officer-initiated calls for service
    d. Arrests
    e. Citations (ordinance, not traffic citations)
    f. Incident reports
    g. Stop and frisk or field interrogation stops
    h. Warnings (not related to traffic)
    i. Response time
    j. Time on scene
    k. Traffic stops or tickets

   Civilian Behavior
    l. Dispatched calls for service
    m. Assault on officers/officer injuries
    n. Resistance against officers
7. Measurement Scale
    a. Dichotomous
    b. Discrete ordinal scaled measure (<10 categories)
    c. Discrete ordinal scaled measure (10+ categories)
    d. Count
    e. Ratios
    f. Continuous measures
    g. Unclear
8. Unit-of-measurement
    a. Officer
    b. Citizen
    c. Incident
    d. Shift
    e. Police unit
    f. Time period
    g. Geographic area
    h. Unclear

Cochrane Risk-of-Bias Tool
(Y=Yes; PY=Probably Yes; PN=Probably No; N=No; NI=No information)

Domain 3: Missing outcome database

9. (3.1) Were data for this outcome available for all, or nearly all, participants randomized? (Y/PY/PN/N/NI)
10. (3.2) If N/PN/NI to 3.1: Is there evidence that the result was not biased by missing outcome data? (Y/PY/PN/N/NI)

11. (3.3) If N/PN to 3.2: Could missingness in the outcome depend on its true value? (Y/PY/PN/N/NI)
12. (3.4) If N/PN to 3.2: Could missingness in the outcome depend on its true value? (Y/PY/PN/N/NI)
13. Optional: What is the predicted direction of bias due to missing outcome data?
    a. N/A
    b. Favors experimental
    c. Favors comparator
    d. Towards the null
    e. Away from the null
    f. Unpredictable

Domain 4: Risk of bias in measurement of the outcome

14. (4.2) Could measurement or ascertainment of the outcome have differed between intervention groups? (Y/PY/PN/N/NI)
15. (4.3) If N/PN/NI 4.2: Were outcome assessors aware of the intervention received by study participants? (Y/PY/PN/N/NI)
16. (4.4) If Y/PY/NI to 4.3: Could assessment of the outcome have been influenced by knowledge of intervention received? (Y/PY/PN/N/NI)
17. (4.5) If Y/PY/NI to 4.4: Is it likely that assessment of the outcome was influenced by knowledge of intervention received? (Y/PY/PN/N/NI)
18. Optional: What is the predicted direction of bias in measurement of the outcome?
    a. N/A
    b. Favors experimental
    c. Favors comparator
    d. Towards the null
    e. Away from the null
    f. Unpredictable

EFFECT SIZE LEVEL CODING

Each unique codeable effect for an eligible outcome is coded as a separate record. The Primary Key that is the unique identifier for each record in the effect size table is the combination of the following fields: StudyID, SubStudyID, OutcomeID, ESID, and CoderID. The StudyID and SubStudyID should uniquely identify which study/substudy is being coded. The OutcomeID indicates which outcome coded at the outcome level is associated with this effect.

For specific effect size computational methods, see Appendix XX. The coding form has input fields for five different methods in which effect sizes could be coded. There are times when a unique effect can be computed in more than one way. The method that is most accurate is selected. For example, a study may report means, standard deviations, and sample size information, as well as an independent t-test associated with these means. Both the former and latter can be used to compute the effect size and should produce the same value. However, the t-test, unless reported out to 3 or more digits,   will lack the precision of the raw means and standard deviations, mainly due to rounding error

In contrast to the above, the same outcome may be analyzed in different ways that would produce different effect sizes. For example, a study may report the raw means and t-test but also report a regression model with a treatment dummy code that adjusts for baseline covariates. In such a situation, code two effect sizes, one based on the means and one on the

regression model (i.e., method 7 on the coding form). These should be coded as separate records in the database.

1. Study ID & Substudy ID. As described above.
2. Outcome ID and Outcome Label. As described above.
3. Effect Size ID. Each effect size is assigned a unique number, starting at 1. For example, if a Study/SubStudy has four effect sizes, these should be numbered 1, 2, 3, 4 in this field.
4. Coder's Initials. As described above.
5. Date Modified. As described above.
6. Page number. The page number in the document in which the specific effect size coded can be located.
7. Notes. [text box]
8. Description of the timing for the effect size. Describes the timing for the effect size. For example, the data for the effect size may reflect a 6-month period following the start of the use of body-worn cameras.
9. Timing for the effect size. For all effect sizes after the start of the use of BWC, select the post-test 1, 2, 3, or 4 sequentially (e.g., the first post-test is 1, next is 2, etc.).
    a. Post-test 1
    b. Post-test 2
    c. Post-test 3
    d. Post-test 4
10. Direction of effect size. Choices here are “favors treatment”; “favors control”; “favors neither”; “unclear”. With body-worn cameras, for many outcomes the term “favors” treatment and control is ambiguous. While it may be the case that a reduction in use of force might clearly “favor treatment,” a reduction in proactive policing may either be favorable or not to the treatment group, depending on one’s perspective. Thus, the following specific rule was adopted for purposes of this study for all of the outcomes: Whenever the outcome declined more for the treatment group than the control group, this was labeled as “favors treatment” in the data for all outcomes. Whenever the outcome increased more for the treatment group than the control group, the label “favors control” was used.
11. Effect size adjusted for baseline variables
    a. Yes
    b. No
    c. Unclear
12. Is the effect reported as significant at p <= .05?
    a. Yes
    b. No
    c. Not tested
    d. Unclear
13. Unit-of-analysis for this effect
    a. Officer
    b. Citizen
    c. Incident
    d. Shift
    e. Police unit
    f. Time period
    g. Geographic area
    h. Other (specify in the notes)
14. Clustered or nested data for this effect?
    a. Yes

    b. No
    c. Unclear
    d. If yes, what is the ICC? (-9 if missing) [number box]
15. Effect size method 1
    a. Treatment group N
    b. Control group N
    c. Treatment group mean/rate
    d. Control group mean/rate
    e. Treatment group standard deviation
    f. Control group standard deviation
    g. Treatment group standard error
    h. Control group standard error
    i. t-value
    j. p-value
    k. Baseline (optional) treatment group N
    l. Baseline (optional) control group N
    m. Baseline (optional) treatment group mean/rate
    n. Baseline (optional) control group mean/rate
    o. Baseline (optional) treatment group standard deviation
    p. Baseline (optional) control group standard deviations
    q. Baseline (optional) treatment group standard error
    r. Baseline (optional) control group standard error
16. Effect Size Method 2 (Binary Outcome – could be represented as a 2 by 2 (treatment/control by success/failure or some other binary outcome)
    a. Treatment group N
    b. Control group N
    c. Treatment group frequency (success or failure)
    d. Control group frequency (success or failure)
    e. Treatment group proportion (success or failure)
    f. Control group proportion (success or failure)
    g. Baseline (optional) treatment group frequency (success or failure)
    h. Baseline (optional) control group frequency (success or failure)
    i. Baseline (optional) treatment group proportion (success or failure)
    j. Baseline (optional) treatment group frequency (success or failure)
17. Effect Size Method 3
    a. Treatment group N
    b. Control group N
    c. Treatment group event (incident) count
    d. Control group event (incident) count
    e. Baseline treatment group event (incident) count
    f. Baseline control group event (incident) count
18. Effect Size Method 4
    a. Treatment group N
    b. Control group N
    c. Type of regression model:
        i. OLS
        ii. Logistic
        iii. Poisson
        iv. quasi-Poisson/negative binomial
        v. OLS multi-level
        vi. Logistic multi-level
        vii. Poisson multi-level

viii. quasi-Poisson/negative binomial multi-level
ix. Time series (ARIMA)
x. Other (specify in notes)

d. B (unstandardized) for treatment effect
e. Standard error for B
f. t or z for B
g. Beta (standardized coefficient)
h. Standard deviation for y (if OLS type model)
i. Odds Ratio (if logistic regression)
j. IRR (if Poisson/NB regression)

19. Effect Size Method 5
    a. Treatment N
    b. Control N
    c. Hand calculated effect size. This is for more complex situations where you, for example, use the online effect size calculator or computed the effect size using R.
    d. Hand calculated variance.
    e. Type
        i. Cohen’s d (these will not be used)
        ii. Logged RIRR (incident rate ratio or relative incident rate ratio)
