# Supplementary material for: Body‐worn cameras’ effects on police officers and citizen behavior: A systematic review
Source: Campbell Syst Rev. 2020 Sep 9;16(3):e1112. doi: 10.1002/cl2.1112 (PMC8356344; doi:10.1002/cl2.1112)
Supplement: Supplementary file 4 — Supporting information [file CL2-16-e1112-s007.pdf]

APPENDIX E: EFFECT SIZE COMPUTATION METHODS

Part of: Lum, C., Koper, C.S., Wilson, D.B., …et al. (2020). Body-worn cameras' effects on police officers and citizen behavior: A systematic review. Campbell Systematic Reviews 2020;e1112. https://doi.org/10.1002/cl2.1112.

This appendix documents the formulas and R functions (i.e., the R statistical programing language) used to compute effect sizes for the meta-analysis of body-worn camera (BWC) studies in this Campbell systematic review. The appendix also simulates data and demonstrates that the formulas and functions generate results that agree with analyses of count data performed using either Poisson or quasi-Poisson regression, depending on the availability of information on the dispersion of the counts, such as standard deviations.

The regression coefficients in a Poisson model (as well as a quasi-Poisson and negative binomial model) are logged incident rate ratios. Once exponentiated (i.e., taking the antilogarithm), they become incident rate ratios. In a difference-in-differences (DiD) analysis where the treatment effect is estimated by the interaction of time (before and during implementation of the body-worn camera) and condition (experimental and control), the exponentiated coefficient for the interaction effect is the ratio of two incident rate ratios, or a relative incident rate ratio (the size of the treatment effect relative to the baseline difference). Below we use the relative incident rate ratio (RIRR) to refer to a DiD analysis and the incident rate ratio (IRR) to refer to a simple ratio in counts or rates during the implementation period (i.e., no baseline data). To make the presentation more general, the term “pre” refers to the baseline period and “post” refers to the period during BWC implementation.

Functions and Formulas

Seven different methods are detailed below that were used to compute effect sizes and associated variances (or standard errors), depending on the data type employed in each study. Most of the studies have outcomes measured in counts, such as the counts of use-of-force incidents, calls-for-service, complaints, etc. The counts may be reported as the total count during the pre and post time periods for the treatment and control groups (method 1 below), or the post time period count for each group (method 2 below). Neither of these methods has information regarding the variability in counts and as such no direct method of estimating over-dispersion.

In contrast, other studies will report the rate or mean count for some unit of time, geographic area, or other unit-of-analysis (e.g., officer or groups of officers) for both the pre and post time periods for treatment and control groups (method 3 below) or for just the post time periods by group (method 4 below). Note that mean counts are incident rates (incidents per month, officer, etc.). In these two situations, it is possible to estimate over-dispersion, a common issue with crime counts. Finally, studies may dichotomize count data, such as coding whether a use-of-force incident did or did not occur during a particular time period (such as a shift) or for an officer during a given time period. Methods 5 and 6 handle these situations with the latter handling both pre and post data. Method 7 includes three methods for handling Poisson related regression models, logistic regression models, and linear regression models (e.g., OLS). There were also

studies for which effect sizes were calculated using "one-off" computations. These are discussed separately for each study with associated R code documenting the computations.

To summarize, the methods used in this meta-analysis to calculate effect sizes are:

- Method 1: Pre and post counts for treatment and control
- Method 2: Post counts only for treatment and control
- Method 3: Pre and post means and standard deviations for treatment and control
- Method 4: Post means and standard deviations for treatment and control
- Method 5: Post only dichotomous outcome data for treatment and control
- Method 6: Pre and post dichotomous outcome data for treatment and control
- Method 7: Regression models

Method 1: Pre and post counts for treatment and control

This method is used for pre and post count data for the treatment and control group with no other information given. Thus, the only information reported is four count values with no information on variability in the counts across areas or across times. The formula for the logged RIRR will work with rates as well, such as crimes per 100,000. However, the variance formula requires raw count totals.

The logged RIRR based on pre/post by treatment/control counts is:

$$\log \text{RIRR} = \log\left(\frac{T_2 C_1}{T_1 C_2}\right),$$

where T is the treatment group count, C is the control group count, and the subscripts represent time—1 for pre and 2 for post. This formula is the same as the cross-product method of computing the odds ratio, leading some to incorrectly label this statistic as a logged odds ratio. An odds ratio is the ratio of two odds, and an odds is the ratio of successes to failures. Typical odds ratio data would be a sample of treatment and control individuals on which a binary outcome, such as recidivism or disease status, is observed post-treatment. In such cases, there is no pre data, and each observation could have been a success or failure. For the pre/post count data by group there is no binary outcome and therefore, no successes or failures. To construct an odds ratio, you would need to assume that the pre counts were failures and the post counts were successes or visa-versa. This is nonsensical, given that each incident contributing to a count isn't free to be observed during either the pre or post time periods. As will be shown via simulation, the above produces the regression coefficient for a time by group interaction in a Poisson model and, as such, is a relative incidence rate ratio, not an odds ratio.

The variance of the logged RIRR, unadjusted for any over-dispersion, is:

$$v_{\log \text{RIRR}} = \frac{1}{T_1} + \frac{1}{T_2} + \frac{1}{C_1} + \frac{1}{C_2}$$

where the terms are defined as above. Note that this method is only appropriate if the counts are directly comparable. That is, it assumes that counts are derived from comparable time periods or areas, at least within each group (i.e., pre and post are comparable). For example, if the pre period is 24 months and the post period is 12 months, then the raw counts cannot be directly

compared and must be converted to rates (i.e., counts divided by the number of months). Methods 3 and 4 below are adapted to handle such mean counts or incident rates.

The R functions for these formulas are:

```
logRIRRCounts <- function(T2,C2,T1,C1) {
     log((T2*C1)/(C2*T1))
}
logRIRRVCounts <-  function(T2,C2,T1,C1) {
     1/T2 + 1/T1 + 1/C2 + 1/C1
}
```

Method 2: Post counts only for treatment and control

This method is used for studies that only report a post count for the treatment and control groups with no other information given. Because there is no information on dispersion or variability in the counts, we must make the assumption that the counts are Poisson distributed.

The logged IRR based on the post only treatment and control counts is:

$$\log IRR = \log\left(\frac{T_2/N_T}{C_2/N_C}\right),$$

where T_2 is the treatment group count for the post time period, C_2 is the control group count for the post time period, and N_T and N_C are the respective sample sizes. The sample sizes might be 1 if the two time periods are equal (e.g., 1 year). If the counts are for a different number of geographic areas, then this is the number of areas (e.g., 10 police sectors versus 15 sectors). N could also be the population of the two areas, or the number of officers or calls for service in each group. In the parlance of count-based models, the N is an offset that converts the counts into incident rates that are directly comparable. The N does not affect statistical power. You could divide a year count by 1, 12, 52, or 365 and get the same logged IRR and the same variance (or standard error). When the two Ns are equal, they cancel each other out and have no effect on the resulting effect size.

The variance, unadjusted for any over-dispersion, is:

$$v_{\log IRR} = \frac{1}{T_1} + \frac{1}{C_2},$$

where the terms are defined as above.

If a study reports the results as incident rates, the logged IRR equation simplifies to the ratio of the two rates. However, the variance must be based on the total counts within each group and *not* the incident rates. Thus, if a study reports the results as the rate per 100,000, these must be converted back into the raw counts to determine the variance.

The R functions for these formulas are:

```
logIRRCountsPost <- function(T2,C2,TN2,CN2) {
       T2 <- ifelse(T2==0,.5,T2)
       C2 <- ifelse(C2==0,.5,C2)
       log((T2/TN2)/(C2/CN2))
```

```
}
logIRRVCountsPost <- function(T2,C2) {
     T2 <- ifelse(T2==0,.5,T2)
     C2 <- ifelse(C2==0,.5,C2)
    1/T2 + 1/C2
}
```

Method 3: Pre and post means and standard deviations for treatment and control

The data for this method are pre and post mean counts (incident rates) by time, area, officer, etc., and standard deviations for the treatment and control groups. The logged RIRR is computed similarly as Method 1, with the exception that we take the cross-products of the means (rates), and not the total counts. Recall that Method 1 assumes that within each condition, the pre and post time periods or areas are comparable. In this method, they need not be. The formula for the logged RIRR is:

$$\log \text{RIRR} = \log\left(\frac{\bar{x}_{T_2}\bar{x}_{C_1}}{\bar{x}_{T_1}\bar{x}_{C_2}}\right),$$

where $\bar{x}$ is a mean count and the subscripts indicate group and time as above. Since this is the same as Method 1 just with means instead of counts, there is no separate R function for this formula.

The standard deviations in the counts within group by time allow for the estimation of over-dispersion using the quasi-Poisson approach. This approach adjusts the standard errors for over-dispersion. The regression coefficients in a quasi-Poisson model are identical to those from a Poisson model, only the standard errors are different. Conveniently, the over-dispersion parameter can be computed from the means, standard deviations, and sample sizes. Note that the sample size must be whatever offset was used for converting the total count into the mean or rate.

Given these data, the quasi-Poisson over-dispersion parameter is computed as:

$$\phi = \left(\sum_{i=1}^{4} \frac{s_i^2(n_i - 1)}{\bar{x}_i}\right) \frac{1}{\sum_{i=1}^{4} n_i - 4}$$

where s_i, n_i, and $\bar{x}_i$ are the standard deviation, sample size, and mean, respectively, for each group and time period. This is set to 1 if the computation returns a value of less than 1. The variance of the logged RIRR is first computed using the variance estimate from Method 1 and then multiplied by the over-dispersion estimate:

$$v_{\text{log RIRR adjusted}} = v_{\text{log RIRR}} \times \phi\ .$$

The R functions used for computing the over-dispersion adjusted variance estimate are:

```
phi1 <- function(TM2,CM2,TM1,CM1,
                TS2,CS2,TS1,CS1,
                TN2,CN2,TN1,CN1) {
    sds <- c(TS2,CS2,TS1,CS1)
    ms <- c(TM2,CM2,TM1,CM1)
```

```
    ns <- c(TN2,CN2,TN1,CN1)
    d <- sum((sds^2*(ns-1)/ms)) * 1/(sum(ns)-4)
    d <- ifelse(d>1,d,1)
    return(d)
    }
logRIRRVOverD <- function(TM2,CM2,TM1,CM1,
                         TS2,CS2,TS1,CS1,
                         TN2,CN2,TN1,CN1) {
    v <- logRIRRVCounts(TM2*TN2,CM2*CN2,TM1*TN1,CM1*CN1)
    phi <- phi1(TM2,CM2,TM1,CM1,
               TS2,CS2,TS1,CS1,
               TN2,CN2,TN1,CN1)
    return(v*phi)
}
```

Method 4: Post only means and standard deviations for treatment and control

This method is essentially the same as Method 3, with the exception of missing pre or baseline data. The comparison is between post mean counts for the treatment and control groups. As with Method 3, data analyzed with Method 4 also have standard deviations associated with each mean. All other statistical issues are the same. The logged IRR is the log of the ratio of the treatment to control mean:

$$\log IRR = \log\left(\frac{\bar{x}_{T_2}}{\bar{x}_{C_2}}\right).$$

This is also the same as the formula as for Method 2, just using the means rather than counts and sample sizes.

As with Method 3, the means, standard deviations, and sample sizes for Method 4 are used to estimate the quasi-Poisson over-dispersion parameter. The modified formula is:

$$\phi = \left(\sum_{i=1}^{2} \frac{s_i^2(n_i - 1)}{\bar{x}_i}\right)\frac{1}{\sum_{i=1}^{2} n_i - 2}.$$

The R functions for the logged IRR and for the over-dispersion adjusted variance estimate are:

```
logIRRMeansPost <- function(TM2,CM2) {
     log(TM2/CM2)
}
phi2 <- function(TM2,CM2,TS2,CS2,TN2,CN2) {
    sds <- c(TS2,CS2)
    ms <- c(TM2,CM2)
    ns <- c(TN2,CN2)
    d <- sum((sds^2*(ns-1)/ms)) * 1/(sum(ns)-2)
    d <- ifelse(d>1,d,1)
    return(d)
    }
logIRRVOverDPost <- function(TM2,CM2,TS2,CS2,TN2,CN2) {
    v <- logIRRVCountsPost(TM2*TN2,CM2*CN2)
    phi <- phi2(TM2,CM2,TS2,CS2,TN2,CN2)
```

```
    return(v*phi)
}
```

Method 5: Post only dichotomous outcome data for treatment and control

In conducting this meta-analysis, we found studies that dichotomized the count data to reflect the presence or absence of an event during some time period or for some other unit-of-analysis, such as an officer. For example, a study might have dichotomized whether or not a use-of-force incident occurred during a shift. In a post-only situation, one could compute either an odds ratio or risk ratio on these data. The risk ratio is the ratio of the probability of success in the treatment group relative to the success in the control group. This probability can be thought of as the rate of successes and has a consistent scaling and similar interpretation as the incident rate ratio. As will be shown through simulation below, if we treat the dichotomized data as counts (0 for no incidents, and 1 for 1 or more incidents), the regression coefficient from a Poisson regression model is the logged risk ratio. Because the data have been censored at one, they are under-dispersed, and do not fully meet the assumptions of the Poisson model. However, the quasi-Poisson model can adjust for both over-dispersion and under-dispersion and, interestingly, the quasi-Poisson model produces standard errors that are close approximations to the correct standard error for a logged risk ratio. This provides the justification for using the risk ratio (and its standard error) as an estimate for our desired IRR effect size for these data.

The formula for the IRR estimated via the risk ratio (RR) is:

$$\log \widehat{\mathrm{IRR}} = \log \mathrm{RR} = \log\left(\frac{T/N_T}{C/N_C}\right) = \log\left(\frac{T \times N_C}{C \times N_T}\right),$$

where T and C are the number of positive events in the treatment and control groups and N_T and N_C are the respective sample sizes.

The formula for the variance of the logged risk ratio is:

$$v_{\log \widehat{\mathrm{IRR}}} = v_{\log \mathrm{RR}} = \frac{N_T - T}{T \times N_T} + \frac{N_C - C}{C \times N_C},$$

where the terms are defined as above.

The R functions for these two formulas are:

```
logRRPostOnly <- function(TN2,CN2,T2,C2) {
    log((T2*CN2)/(C2*TN2))
}
logRRVPostOnly <- function(TN2,CN2,T2,C2) {
    (TN2 - T2)/(T2*TN2) +
     (CN2 - C2)/(C2*CN2)
}
```

Method 6: Pre and post dichotomous outcome data for treatment and control

A variation on Method 5 is dichotomized data both pre and post for the treatment and control groups. For example, a study may report the proportion of officers with a complaint both pre and post for the treatment and control groups. The goal is to have an estimate of the treatment effect

adjusted for baseline differences, or a DiD estimate. This is accomplished by subtracting the logged risk ratio on the pre data from the logged risk ratio on the post data, as shown below.

$$\log \widehat{\mathrm{RIRR}} = \log\left(\frac{T_2 \times N_C}{C_2 \times N_T}\right) - \log\left(\frac{T_1 \times N_C}{C_1 \times N_T}\right).$$

The subscripts 1 and 2 denote the pre and post time periods and other terms are defined as above.

The variance for this method is an extension of the variance for Method 5, adding the variability introduced from the pre data.

$$v_{\log \widehat{\mathrm{RIRR}}} = \frac{N_T - T_2}{T_2 \times N_T} + \frac{N_C - C_2}{C_2 \times N_C} + \frac{N_T - T_1}{T_1 \times N_T} + \frac{N_C - C_1}{C_1 \times N_C}.$$

The R functions for these equations are:

```
logRRPrePost <- function(TN,CN,T2,C2,T1,C1) {
    log((T2 * CN)/(C2 * TN)) - log((T1 * CN)/(C1 * TN))
}
logRRVPrePost <- function(TN,CN,T2,C2,T1,C1) {
    (TN-T2)/(T2*TN) + (CN-C2)/(C2*CN) +
     (TN-T1)/(T1*TN) + (CN-C1)/(C1*CN)
}
```

Method 7: Regression models

Research on the impacts of BWCs also uses Poisson-based regression models (Poisson, quasi-Poisson, or negative-binomial), logistic regression models, and ordinary least squares models. Often there were also multi-level or mixed-effects models that adjusted for hierarchical or clustered data.

Method 7a: Poisson/negative binomial models

In the case of Poisson type models, the regression coefficient for either the treatment by time effect (DiD) or the treatment effect (non-DiD) was the logged RIRR or logged IRR, depending on whether the model included pre and post data or only post data. Thus, we coded as our effect size the regression coefficient and associated standard error for treatment by time or just for treatment.

Method 7b: Logistic regression models

The regression coefficient for the treatment effect from a logistic regression model is a logged odds ratio. In the case of post only data, it is a logged odds ratio for the treatment effect adjusted for any other variables in the model. In the case of both pre and post data, the treatment by time interaction is a DiD coefficient and reflects the logged odds ratio post minus the logged odds ratio pre, adjusted for any coefficients. When base-rates are low (or high), such as less than .1, the odds ratio and risk ratio are fairly similar. For example, if the treatment success rate is .08 and the control success rate is .10, then the risk ratio is .8 whereas the odds ratio is .783. They diverge as the probabilities approach .5. Odds ratios can be converted to risk ratio using the following formula:

$$\mathrm{RR} = \frac{\mathrm{OR}}{(1-p) + (\mathrm{OR} \times p)},$$

where OR is the odds ratio and p is the probability of success in the control group, assuming that the control group was in the denominator of the odds ratio. If the odds ratio is less than 1, then p is the larger of the two proportions, and conversely if the odds ratio is greater than 1, then p is the smaller of the two proportions.

Since many of these models are either adjusting for covariates or are multi-level models, the simple variance based on the 2 by 2 frequencies is biased. To obtain a more valid estimate of the variance and one that is consistent with the logistic regression model, we rescaled the reported standard error for the logistic regression coefficient to maintain the coefficient to standard error ratio. Put more simply, we ensured that the t or z for the calculated effect size is was same as for the original coefficient. The formula for this is:

$$v_{\log \mathrm{RR}} = \left(\frac{\log(\mathrm{RR}) \times \mathrm{SE}_{\log(\mathrm{OR})}}{\log(\mathrm{OR})}\right)^2,$$

where log(OR) is the regression coefficient from the logistic regression model, $SE_{\log(\mathrm{OR})}$ is the standard error for the regression coefficient from the logistic regression model, and log(OR) is the risk ratio obtained from the prior formula that converts the odds ratio into a risk ratio.

The R functions for these equations are:

```
logRRlogOR <- function(lgOR,p1,p2) {
  OR <- exp(lgOR)
  psmall <- ifelse(p1<p2,p1,p2)
  plarge <- ifelse(p1>p2,p1,p2)
  p <- ifelse(OR<1, plarge, psmall)
  return(log(OR/((1-p)+(p*OR))))
}
logRRVlogOR <- function(lgRR,lgOR,se) {
  ((lgRR*se)/lgOR)^2
}
```

This method is accurate for post only models. For DiD models, the conversion of the odds ratio into a risk ratio is only approximate, given that it is actually the ratio of two odds ratios. When possible for the DiD cases, we estimated the proportions by each group by time from the regression model, assuming the full model was reported. We then used these four proportions to compute the logged RIRR. These computations are detailed in the section on study-specific computations. If this was not possible, the above conversion was used.

Method 7c: Ordinary and generalized linear regression models

Several studies reported results from ordinary least squares (OLS) regression or mixed-effects linear models. In some cases, the study also reported means. If the model did not include baseline covariates, then we computed the logged RIRR or logged IRR from the reported means. In cases where the statistical model adjusted for covariates, then the logged RIRR (for DiD

models) or logged IRR for a simple mean difference models was computed using the following formula:

$$\log \text{RIRR} = \log\left(\frac{\bar{x}_c + B}{\bar{x}_c}\right),$$

where $\bar{x}_c$ is the post period mean for the control group and B is the regression coefficient for the treatment effect. If the mean for the control group was not reported, then it was estimated from the regression model.

The variance for the effect size was calculated from the standard error for the regression coefficient if the model adjusted for clustering or other statistical issues. This maintains the effect size to standard error ratio that was present for the regression coefficient and its standard error, thus accounting for clustering, covariates, etc. The formula was:

$$v_{\log \text{RIRR}} = \left(\frac{\log(\text{RIRR}) \times \text{SE}_B}{B}\right)^2,$$

where B is the regression coefficient and SE_B is the standard error of the regression coefficient.

The R functions for these formulas are:

```
logRIRRolsB <- function(B,x) {
  log((x+B)/x)
}
logRIRRVolsB <- function(lgrirr,B,s) {
  ((lgrirr*s)/B)^2
}
```

Simulations and Verification of Function Accuracy

Below we simulated data for each of the situations above and compare the results from either a Poisson and quasi-Poisson regression model to the results of the above functions. For Methods 1-4, the formulas and functions reproduce the regression model results. For Methods 5-6, the effect sizes are reproduced but the standard errors (variances) differ slightly. However, for these models the computed standard errors are actually more accurate as the method is consistent with the nature of the data. The methods for logistic and OLS type regression models are also shown to be sufficiently accurate for our purpose.

The simulated data below is generated using the negative binomial distribution function with a moderate amount of over-dispersion. A total of 48 counts are generated, with 12 counts pre and post for each group.

```
set.seed(12345)
Crime <- c(rnbinom(12, 500, .2),
           rnbinom(12, 400, .2),
           rnbinom(24, 500, .2))
Time <- c(rep(1,12),rep(2,12),rep(1,12),rep(2,12))
Group   <- c(rep(1,24),rep(2,24))
data <- as.data.frame(cbind(Crime,Group,Time))
```

```r
table <- aggregate(Crime ~ Time + Group,
                   data=data, function(x) {
             c("Count"=sum(x),"Mean"=mean(x),
               "SD"=sd(x),"N"=length(x)) })
table$Count <- table$Crime[,1]
table$Mean  <- table$Crime[,2]
table$SD    <- table$Crime[,3]
table$N     <- table$Crime[,4]
table$Crime <- NULL
pander(table)
```

Time	Group	Count	Mean	SD	N
1	1	24055	2005	116.8	12
2	1	19721	1643	110.9	12
1	2	23926	1994	94.69	12
2	2	23712	1976	102.6	12

Verification of Method 1

Below is a simple Poisson regression model with time, group, and the interaction for time and group as the independent variables as well as the results from the R functions for Method 1. These functions accurately reproduce the regression results. For the Poisson model, the result of interest is the time by group interaction. Because the model reports standard errors, we have reported the square-root of the variance returned from the function.

```r
logRIRR <- logRIRRCounts(table$Count[4],table$Count[2],
                         table$Count[3],table$Count[1])
SE <-   sqrt(logRIRRVCounts(table$Count[4],table$Count[2],
                         table$Count[3],table$Count[1]))
pander(summary(glm(Crime ~ Time*Group,
                   family="poisson", data=data)))
```

	Estimate	Std. Error	z value	Pr(>|z|)
**(Intercept)**	7.997	0.03282	243.6	0
**Time**	-0.3883	0.02129	-18.24	2.317e-74
**Group**	-0.1951	0.02065	-9.446	3.506e-21
**Time:Group**	0.1897	0.01328	14.29	2.629e-46

(Dispersion parameter for poisson family taken to be 1)

Null deviance:	860.3 on 47 degrees of freedom
Residual deviance:	266.3 on 44 degrees of freedom

```r
pander(as.data.frame(cbind(logRIRR,SE)))
```

logRIRR	SE
0.1897	0.01328

Verification of Method 2

Below is a simple Poisson regression model with group as the only independent variable, given that the data is post-test only. The R functions for Method 2 are also applied to the counts. These functions accurately reproduce the regression results.

```
logIRR <- logIRRCountsPost(table$Count[4],
                           table$Count[2],
                           table$N[4],
                           table$N[2])
SE <-  sqrt(logIRRVCountsPost(table$Count[4],
                              table$Count[2]))
pander(summary(glm(Crime ~ Group,
                   family="poisson", data=data[ Time==2, ])))
```

	Estimate	Std. Error	z value	Pr(>|z|)
**(Intercept)**	7.22	0.01565	461.3	0
**Group**	0.1843	0.009637	19.12	1.623e-81

(Dispersion parameter for poisson family taken to be 1)

Null deviance:	508.1 on 23 degrees of freedom
Residual deviance:	140.8 on 22 degrees of freedom

```
pander(as.data.frame(cbind(logIRR,SE)))
```

logIRR	SE
0.1843	0.009637

Verification of Method 3

Method 3 adjusts for over-dispersion using the quasi-Poisson over-dispersion estimator. The R functions for Method 3 and the quasi-Poisson regression model on these data produce identical results, establishing the accuracy of the formulas and R functions that implement them.

```
logRIRR <- logRIRRCounts(table$Mean[4],table$Mean[2],
                         table$Mean[3],table$Mean[1])
phi <- phi1(table$Mean[4],table$Mean[2],
            table$Mean[3],table$Mean[1],
            table$SD[4],table$SD[2],
            table$SD[3],table$SD[1],
            table$N[4],table$N[2],
            table$N[3],table$N[1])
SE <- sqrt(logRIRRVOverD(table$Mean[4],table$Mean[2],
            table$Mean[3],table$Mean[1],
            table$SD[4],table$SD[2],
            table$SD[3],table$SD[1],
            table$N[4],table$N[2],
            table$N[3],table$N[1]))
```

```
pander(summary(glm(Crime ~ Time*Group,
                   family="quasipoisson", data=data)))
```

	Estimate	Std. Error	t value	Pr(>|t|)
**(Intercept)**	7.997	0.08058	99.24	2.177e-53
**Time**	-0.3883	0.05226	-7.431	2.682e-09
**Group**	-0.1951	0.05069	-3.848	0.0003814
**Time:Group**	0.1897	0.03259	5.82	6.212e-07

(Dispersion parameter for quasipoisson family taken to be 6.027303)

Null deviance:	860.3 on 47 degrees of freedom
Residual deviance:	266.3 on 44 degrees of freedom

```
pander(as.data.frame(cbind(logRIRR,SE,phi)))
```

logRIRR	SE	phi
0.1897	0.03259	6.027

Verification of Method 4

Recall that Method 4 is just Method 3 without the pre-test data. The analyses below establish the accuracy of the formulas and R functions which implement them.

```
logRIRR <- logIRRCountsPost(table$Mean[4],table$Mean[2],table$N[4],table$N[2])
phi <- phi2(table$Mean[4],table$Mean[2],
            table$SD[4],table$SD[2],
            table$N[4],table$N[2])
SE <- sqrt(logIRRVOverDPost(table$Mean[4],table$Mean[2],
            table$SD[4],table$SD[2],
            table$N[4],table$N[2]))
pander(summary(glm(Crime ~ Group, family="quasipoisson",
                   data=data[ Time==2, ])))
```

	Estimate	Std. Error	t value	Pr(>|t|)
**(Intercept)**	7.22	0.03961	182.3	1.787e-36
**Group**	0.1843	0.02439	7.557	1.499e-07

(Dispersion parameter for quasipoisson family taken to be 6.402818)

Null deviance:	508.1 on 23 degrees of freedom
Residual deviance:	140.8 on 22 degrees of freedom

```
pander(as.data.frame(cbind(logRIRR,SE,phi)))
```

logRIRR	SE	phi
0.1843	0.02439	6.403

Verification of Method 5

Method 5 and 6 are based on binary or dichotomous data. We simulate binary data for the treatment and control group with a population failure rate (proportion of 1s) for the treatment group of 0.10 and 0.15 for the control group. We then estimate the logged RIRR using Method 5. We also analyze these data using a quasi-Poisson model. Notice that the over-dispersion parameter is less than 1, reflecting under-dispersion. The regression coefficient for the treatment effect equals the logged risk ratio computed on these data. The computed standard error is approximately equal to the standard error for the treatment effect from the quasi-Poisson model. In this case, the computed standard error is more accurate than that from the quasi-Poisson model.

```
set.seed(54321)
y <- c(rbinom(100,1,.10),rbinom(100,1,.15))
group <- c(rep(0,100),rep(1,100))
data <- as.data.frame(cbind(y,group))
data$group <- factor(group, labels=c("Treatment","Control"))
table <- table(data$group,data$y)
table <- addmargins(table, 2)
logRR <-logRRPostOnly(table[2,3],table[1,3],
                      table[2,2],table[1,2])
SE <- sqrt(logRRVPostOnly(table[2,3],table[1,3],
                          table[2,2],table[1,2]))
pander(table)
```

	0	1	Sum
**Treatment**	88	12	100
**Control**	83	17	100

```
pander(summary(glm(y~group, family="quasipoisson", data=data)))
```

	Estimate	Std. Error	t value	Pr(>|t|)
**(Intercept)**	-2.12	0.2683	-7.903	1.851e-13
**groupControl**	0.3483	0.3504	0.9941	0.3214

(Dispersion parameter for quasipoisson family taken to be 0.8636364)

Null deviance:	112.0 on 199 degrees of freedom
Residual deviance:	111.1 on 198 degrees of freedom

```
pander(as.data.frame(cbind(logRR,SE)))
```

logRR	SE
0.3483	0.3495

Verification of Method 6

For Method 6, we need both pre and post binary data for each group. Such data is simulated below, adding to the data used from the verification of Method 5. The pre data is simulated twice, first with no correlation between the pre and post data (the actual data may have a non-

zero correlation due to chance, but the random distributions from which they are sampled are uncorrelated), and second with a correlation between the pre and post observations. As with Method 5, we analyzed these data using a quasi-Poisson model. In this case, the treatment effect is the time by group interaction. The regression coefficient for the treatment by group effect equals the computed RIRR or difference between the pre and post logged risk ratios. The computed standard error is approximately equal to the standard error for the treatment effect from the quasi-Poisson model. In this case, the computed standard error is more accurate.

```
library("tidyr")
data$y1 <- c(rbinom(100,1,.10),rbinom(100,1,.15))
data$y2 <- data$y
data$y <- NULL ## drop y (it is now y2)
datalong <- gather(data, time, y, y1:y2, factor_key=TRUE)
table <- table(datalong$time,datalong$group,datalong$y)
logRIRR <- logRRPrePost(100,100,table[2,2,2], table[2,1,2],
                              table[1,2,2],table[1,1,2])
SE <- sqrt(logRRVPrePost(100,100,table[2,2,2], table[2,1,2],
                              table[1,2,2],table[1,1,2]))
pander(table)
```

		0	1
y1	Treatment	89	11
	Control	85	15
y2	Treatment	88	12
	Control	83	17

```
pander(summary(glm(y~group*time, family="quasipoisson", data=datalong)))
```

	Estimate	Std. Error	t value	Pr(>|t|)
**(Intercept)**	-2.207	0.2814	-7.843	4.122e-14
**groupControl**	0.3102	0.3705	0.8371	0.403
**timey2**	0.08701	0.3896	0.2233	0.8234
**groupControl:timey2**	0.03815	0.511	0.07466	0.9405

(Dispersion parameter for quasipoisson family taken to be 0.8712122)

Null deviance:	218.3 on 399 degrees of freedom
Residual deviance:	216.6 on 396 degrees of freedom

```
pander(as.data.frame(cbind(logRIRR,SE)))
```

logRIRR	SE
0.03815	0.5096

```
data$y1 <- rbinom(200,1,.40)*data$y2
PrePostR <- cor(data$y1,data$y2)
```

```r
datalong <- gather(data, time, y, y1:y2, factor_key=TRUE)
table <- table(datalong$time,datalong$group,datalong$y)
logRIRR <- logRRPrePost(100,100,table[2,2,2], table[2,1,2],
                              table[1,2,2],table[1,1,2])
SE <- sqrt(logRRVPrePost(100,100,table[2,2,2], table[2,1,2],
                              table[1,2,2],table[1,1,2]))
pander(table)
```

		0	1
y1	Treatment	95	5
	Control	92	8
y2	Treatment	88	12
	Control	83	17

```r
pander(summary(glm(y~group*time,
                   family="quasipoisson", data=datalong)))
```

	Estimate	Std. Error	t value	Pr(>|t|)
**(Intercept)**	-2.996	0.4252	-7.045	8.257e-12
**groupControl**	0.47	0.542	0.8671	0.3864
**timey2**	0.8755	0.5061	1.73	0.08444
**groupControl:timey2**	-0.1217	0.6499	-0.1873	0.8515

(Dispersion parameter for quasipoisson family taken to be 0.9040406)

Null deviance:	189.3 on 399 degrees of freedom
Residual deviance:	181.5 on 396 degrees of freedom

```r
pander(as.data.frame(cbind(logRIRR,SE,PrePostR)))
```

logRIRR	SE	PrePostR
-0.1217	0.6536	0.6403

Verification of Method 7b

This method converts the results from a logistic regression model, including mixed-effects (hierarchical) logistics regression models into the desired logged IRR or logged RIRR. We first simulate the former. The conversion functions reproduce the logged risk ratio from the 2 by 2 table and the standard error is a close approximation. If the model includes covariates then this would be a logged risk ratio adjusted for covariates. Additionally, the value computed from the raw proportions would differ somewhat from those computed with this method. This logged risk ratio is our estimate of the logged incident rate ratio.

```r
set.seed(54321)
y <- c(rbinom(100,1,.10),rbinom(100,1,.15))
group <- c(rep(0,100),rep(1,100))
```

```r
data <- as.data.frame(cbind(y,group))
data$group <- factor(group, labels=c("Treatment","Control"))
table <- table(data$group,data$y)
table <- addmargins(table, 2)
logRR <-logRRPostOnly(table[2,3],table[1,3],
                     table[2,2],table[1,2])
SE <- sqrt(logRRVPostOnly(table[2,3],table[1,3],
                         table[2,2],table[1,2]))
model <- glm(y~group, family="binomial", data=data)
logRRfromOR <- logRRlogOR(model$coefficients[2],
                          table[1,2]/table[1,3],
                          table[2,2]/table[2,3])
SEfromOR <- sqrt(logRRVlogOR(logRRfromOR,
                          summary(model)$coefficients[2,1],
                          summary(model)$coefficients[2,2]))
pander(table)
```

	0	1	Sum
**Treatment**	88	12	100
**Control**	83	17	100

```r
pander(model)
```

Fitting generalized (binomial/logit) linear model: y ~ group

	Estimate	Std. Error	z value	Pr(>|z|)
**(Intercept)**	-1.992	0.3077	-6.475	9.501e-11
**groupControl**	0.4068	0.4069	0.9998	0.3174

```r
pander(as.data.frame(cbind(logRR,SE,logRRfromOR,SEfromOR)))
```

	logRR	SE	logRRfromOR	SEfromOR
**groupControl**	0.3483	0.3495	0.3483	0.3484

The formulas and function for converting the results from logistic regression models with both pre and post data also work reasonably well, although in this case the conversion is approximate. The conversion is assuming a simple odds ratio and not the ratio of two odds ratios. Ideally in these cases, we would estimate the pre and post success proportions from the regression model and compute the RIRR directly from those. The conversion method tested where was only used if that was not possible (i.e., the study did not report the full regression model and enough information about model to recover these proportions).

```r
library("tidyr")
data$y1 <- c(rbinom(100,1,.10),rbinom(100,1,.15))
data$y2 <- data$y
data$y <- NULL ## drop y (it is now y2)
datalong <- gather(data, time, y, y1:y2, factor_key=TRUE)
table <- table(datalong$time,datalong$group,datalong$y)
logRIRR <- logRRPrePost(100,100,table[2,2,2], table[2,1,2],
                              table[1,2,2],table[1,1,2])
```

```
SE <- sqrt(logRRVPrePost(100,100,table[2,2,2], table[2,1,2],
                               table[1,2,2],table[1,1,2]))

model <- glm(y~group*time, family="binomial", data=datalong)
logRIRRfromOR <- logRRlogOR(summary(model)$coefficients[4,1],
                          table[2,1,2]/100,
                          table[2,2,2]/100)
SEfromOR <- sqrt(logRRVlogOR(logRIRRfromOR,
                          summary(model)$coefficients[4,1],
                          summary(model)$coefficients[4,2]))
pander(table)
```

		0	1
y1	Treatment	89	11
	Control	85	15
y2	Treatment	88	12
	Control	83	17

```
#pander(summary(model))
pander(as.data.frame(cbind("log RIRR" =logRIRR,SE,
                          "log RIRR from logistic Reg." = logRIRRfromOR,
                          "SE" =SEfromOR)))
```

log RIRR	SE	log RIRR from logistic Reg.	SE
0.03815	0.5096	0.04445	0.5161

Finally, the code below demonstrates that the original proportions can be recovered from the regression coefficients.

```
b <- model$coefficients
control_pre  <- sum(b*c(1,1,0,0))
control_post <- sum(b*c(1,1,1,1))
tx_pre       <- sum(b*c(1,0,0,0))
tx_post      <- sum(b*c(1,0,1,0))
exp(control_pre)/(exp(control_pre)+1)

## [1] 0.15

exp(control_post)/(exp(control_post)+1)

## [1] 0.17

exp(tx_pre)/(exp(tx_pre)+1)

## [1] 0.11

exp(tx_post)/(exp(tx_post)+1)

## [1] 0.12
```

Verification of Method 7c

Linear regression models, such as OLS and linear mixed-effects GLM models, presented a special problem in this meta-analysis. For method detailed in this section to work, either the means must be available and reported, or it must be possible to estimate them from the model. This method works for both a post-only treatment effect and a DiD model. For the post-only model, the formula and function reproduce the same value for the logged IRR as one gets from a direct calculation from the raw data, as well as a close approximation to the standard error, as expected. For pre and post data, the logged RIRR will differ from that of the Poisson model as the difference between the pre treatment and control means or counts differ. The OLS is comparing absolute change, whereas the Poisson is comparing relative change. When the two baseline counts are equal, this method and the Poisson model will be equal. The more the baseline counts differ, the more these methods will differ. However, this verifies that this method provides a reasonable approximation to the desired logged RIRR. In RCTs, the baseline counts should be roughly equal and only differ by chance. In quasi-experiments, the baseline counts might differ more substantially. However, this method still produces a workable effect size estimate.

```
set.seed(12345)
Crime <- c(rnbinom(12, 500, .2),
           rnbinom(12, 400, .2),
           rnbinom(24, 500, .2))
Time <- c(rep(0,12),rep(1,12),rep(0,12),rep(1,12))
Group   <- c(rep(0,24),rep(1,24))
data <- as.data.frame(cbind(Crime,Group,Time))
table <- aggregate(Crime ~ Group,
                   data=data, function(x) {
             c("Count"=sum(x),"Mean"=mean(x),
               "SD"=sd(x),"N"=length(x)) })
table$Count <- table$Crime[,1]
table$Mean  <- table$Crime[,2]
table$SD    <- table$Crime[,3]
table$N     <- table$Crime[,4]
table$Crime <- NULL
pander(table)
```

Group	Count	Mean	SD	N
0	43776	1824	215.5	24
1	47638	1985	96.97	24

```
model1 <- lm(Crime ~ Group, data=data)
model2 <- glm(Crime ~ Group, data=data, family="quasipoisson")
logRIRR <- logRIRRolsB(model1$coefficients[2],table$Mean[1])
SE <- sqrt(logRIRRVolsB(logRIRR,model1$coefficients[2],
                        summary(model1)$coefficients[2,2]))
pander(table)
```

Group	Count	Mean	SD	N
0	43776	1824	215.5	24

1	47638	1985	96.97	24

```
pander(summary(model1))
```

	Estimate	Std. Error	t value	Pr(>|t|)
**(Intercept)**	1824	34.11	53.48	4.561e-43
**Group**	160.9	48.23	3.336	0.001687

Fitting linear model: Crime ~ Group

Observations	Residual Std. Error	R^2	Adjusted R^2
48	167.1	0.1948	0.1773

```
pander(summary(model2))
```

	Estimate	Std. Error	t value	Pr(>|t|)
**(Intercept)**	7.509	0.01857	404.3	2.505e-83
**Group**	0.08455	0.02573	3.286	0.001947

(Dispersion parameter for quasipoisson family taken to be 15.09718)

Null deviance:	860.3 on 47 degrees of freedom
Residual deviance:	697.0 on 46 degrees of freedom

```
pander(as.data.frame(cbind(logRIRR,SE)))
```

	logRIRR	SE
**Group**	0.08455	0.02534

```
Crime <- c(rnbinom(12, 500, .2),
           rnbinom(12, 400, .2),
           rnbinom(24, 500, .2))
Time <- c(rep(0,12),rep(1,12),rep(0,12),rep(1,12))
Group   <- c(rep(0,24),rep(1,24))
data <- as.data.frame(cbind(Crime,Group,Time))
table <- aggregate(Crime ~ Group + Time,
                   data=data, function(x) {
             c("Count"=sum(x),"Mean"=mean(x),
               "SD"=sd(x),"N"=length(x)) })
table$Count <- table$Crime[,1]
table$Mean  <- table$Crime[,2]
table$SD    <- table$Crime[,3]
table$N     <- table$Crime[,4]
table$Crime <- NULL
model1 <- lm(Crime ~ Group * Time, data=data)
model2 <- glm(Crime ~ Group * Time, data=data, family="quasipoisson")
logRIRR <- logRIRRolsB(model1$coefficients[4],table$Mean[3])
SE <- sqrt(logRIRRVolsB(logRIRR,model1$coefficients[4],
                          summary(model1)$coefficients[4,2]))
pander(table)
```

Group	Time	Count	Mean	SD	N

0	0	23910	1992	96.61	12
1	0	24083	2007	85.71	12
0	1	19269	1606	79.7	12
1	1	24249	2021	101.7	12

```
pander(summary(model1))
```

	Estimate	Std. Error	t value	Pr(>|t|)
**(Intercept)**	1993	26.37	75.57	3.269e-48
**Group**	14.42	37.29	0.3866	0.7009
**Time**	-386.7	37.29	-10.37	2.139e-13
**Group:Time**	400.6	52.73	7.597	1.542e-09

*Fitting linear model: Crime ~ Group * Time*

Observations	Residual Std. Error	R^2	Adjusted R^2
48	91.33	0.7982	0.7844

```
pander(summary(model2))
```

	Estimate	Std. Error	t value	Pr(>|t|)
**(Intercept)**	7.597	0.01349	563	1.623e-86
**Group**	0.007209	0.01905	0.3785	0.7069
**Time**	-0.2158	0.0202	-10.68	8.373e-14
**Group:Time**	0.2227	0.02772	8.033	3.619e-10

(Dispersion parameter for quasipoisson family taken to be 4.353928)

Null deviance:	984.0 on 47 degrees of freedom
Residual deviance:	193.3 on 44 degrees of freedom

```
pander(as.data.frame(cbind(logRIRR,SE)))
```

	logRIRR	SE
**Group:Time**	0.2227	0.02932

R Code for Effect Size Calculations for Specific Studies

Data were not always presented in studies in such a way as to allow direct calculation of the logged RIRR or logged IRR using the methods detailed in the prior sections. In these cases, either preliminary calculations were necessary or one-off methods needed to be implemented. These computations are detailed below for each study where this was the case.

Study ID S272: Ariel (2016, 2017) Denver, CO

Coding for study S272 is based on two references:

1. R272: Ariel, B. (2016). Increasing cooperation with the police using body worn cameras. *Police Quarterly, 19,* 326-362.

2. R273: Ariel, B. (2016). Police body cameras in large police department. *Journal of Criminal Law & Criminology, 106(4),* 729-768.

This study has one geographic area for the treatment group and five for the control group. The raw counts were reported for the five control areas for the following outcomes: use of force incidents, misconduct complaints, and force complaints. Standard deviations were not reported but can be computed from these control counts for both the baseline (pre) and implementation (post) periods. These standard deviations are then used to estimate the variance, adjusting for overdispersion, using Method 3. These data are reported in reference ID R273.

The code below computes the total count and standard deviation of the counts for the control group across these three outcomes.

```
## use of force incidents data
force_pre  <- c(101,118,135,157,58)
force_post <- c(67,49,57,77,44)
## misconduct complaints data
mis_pre <- c(83,120,91,84,30)
mis_post <- c(44,63,64,41,33)
## force complaints data
force_comp_pre <- c(10,17,14,22,6)
force_comp_post <- c(18,18,23,11,8)
## create table
sds <- c("Force Pre" = sd(force_pre), "Force Post" = sd(force_post),
         "Misconduct Pre" = sd(mis_pre), "Miconduct Post" = sd(mis_post),
         "Complaints Pre" = sd(force_comp_pre), "Complaints Post" = sd(force_comp
_post))
counts <- c("Force Pre" = sum(force_pre), "Force Post" = sum(force_post),
         "Misconduct Pre" = sum(mis_pre), "Miconduct Post" = sum(mis_post),
         "Complaints Pre" = sum(force_comp_pre), "Complaints Post" = sum(force_co
mp_post))
table <- rbind(counts,sds)
row.names(table) <- c("Counts","Standard Deviation")
pander(table)
```

Table continues below

	Force Pre	Force Post	Misconduct Pre
**Counts**	569	294	408
**Standard Deviation**	37.45	13.39	32.53

	Miconduct Post	Complaints Pre	Complaints Post
**Counts**	245	69	78
**Standard Deviation**	13.84	6.181	6.025

Study ID S416: Wallace et al. (2018) Spokane, WA

Coding for study S416 is based on two references:

1. R416: White, M.D., Gaub, J.E., & Todak, N. (2018). Exploring the potential for body-worn cameras to reduce violence in police-citizen encounters. *Policing: A Journal of Policy and Practice, 12(1),* 66–76.

2. R411: Wallace, D., White, M.D., Gaub, J.E., & Todak, N. (2018). Body-worn cameras as a potential source of de-policing: Testing for camera-induced passivity. *Criminology, 56(3),* 481–509. doi: 10.1111

The computations below are based on information from R411, tables 1, 3, and 4. The effect size is based on the regression coefficient for TX Officer x RCT Period (i.e., treatment and control officers by pre and during periods). These are logistic regression models. Method 7b needs the base-rate each outcome to convert the logged odds ratio into our logged RIRR. We could use the overall proportions listed in table 1, but below we estimate the proportions based on the overall mean and the regression coefficients. This makes a fairly trivial difference.

```
## officer initiated call
m <- .407 # overall mean
mlogit <- log(m/(1-m))
b <- .077
logit1 <- mlogit + b/2
logit2 <- mlogit - b/2
p1 <- exp(logit1)/(exp(logit1)+1)
p2 <- exp(logit2)/(exp(logit2)+1)
table <- rbind("Officer initialed calls Treatment p =" = p1,
  "Officer initialed calls Control p =" = p2)
pander(table)
```

**Officer initialed calls Treatment p =**	0.4163
**Officer initialed calls Control p =**	0.3977

```
## arrest
m <- .055 # overall mean
mlogit <- log(m/(1-m))
b <- .139
logit1 <- mlogit + b/2
logit2 <- mlogit - b/2
p1 <- exp(logit1)/(exp(logit1)+1)
p2 <- exp(logit2)/(exp(logit2)+1)
table <- rbind("Arrests Treatment p =" = p1,
  "Arrests Control p =" = p2)
pander(table)
```

**Arrests Treatment p =**	0.05873
**Arrests Control p =**	0.0515

S600: Braga et al. (2018) Boston, MA

The is one reference associated with this study.

R600: Braga, A.A., Barao, L.M., Zimmerman, G.M., Douglas, S., & Sheppard, K. (2019). Measuring the direct and spillover effects of body worn cameras on the civility of police-citizen encounters and police work activities. *Journal of Quantitative Criminology*. doi:10.1007/s10940-019-09434-9

There were seven effect sizes extracted from this study. Two were based on Poisson models, and five were from panel OLS models. For these models, we extracted the mean counts pre and post by group and then computed the logged RIRR using Method 3. The standard error was rescaled as detailed in Method 7c. The code below shows the computations.

Note that crime incidents effect changes directions but is essentially null in the OLS and in the estimated model. This reflects the difference between a DiD in absolute change versus a DiD in relative (percent) change.

The models are reported on page 23. The independent variables are in the following order and dummy coded as follows:

- Treatment by time interaction: multiple the treatment and time dummy codes
- Treatment: 1 = treatment; 0 = control
- Time: 1 = intervention; 0 = baseline
- Constant equals 1 for all observations

```
## regression coefficients
dispatched <- c(-1.687,3.846, .548,16.451)
oic        <- c(-.870, 2.264, .336, 7.979)
ci         <- c(-.055, 1.621, -.795, 8.941)
ar         <- c( .018,  .126,-.074, 1.72)
fio        <- c( .456,-1.048,-.981, 3.067)

## Contrasts (independent variable dummy coding)
control_t1_mean <- c(0, 0, 0, 1)
control_t2_mean <- c(0, 0, 1, 1)
tx_t1_mean      <- c(0, 1, 0, 1)
tx_t2_mean      <- c(1, 1, 1, 1)
## Logged RIRRs
logRIRRCounts <- function(T2,C2,T1,C1) {
    log((T2*C1)/(C2*T1))
}
## Compute logged RIRR
dispatchedRIRR <- logRIRRCounts(sum(tx_t2_mean*dispatched),
              sum(control_t2_mean*dispatched),
              sum(tx_t1_mean*dispatched),
              sum(control_t1_mean*dispatched))
oicRIRR <- logRIRRCounts(sum(tx_t2_mean*oic),
              sum(control_t2_mean*oic),
              sum(tx_t1_mean*oic),
              sum(control_t1_mean*oic))
```

```r
ciRIRR <- logRIRRCounts(sum(tx_t2_mean*ci),
              sum(control_t2_mean*ci),
              sum(tx_t1_mean*ci),
              sum(control_t1_mean*ci))
arRIRR <- logRIRRCounts(sum(tx_t2_mean*ar),
              sum(control_t2_mean*ar),
              sum(tx_t1_mean*ar),
              sum(control_t1_mean*ar))
fioRIRR <- logRIRRCounts(sum(tx_t2_mean*fio),
              sum(control_t2_mean*fio),
              sum(tx_t1_mean*fio),
              sum(control_t1_mean*fio))
RIRRs <- c(dispatchedRIRR,oicRIRR,ciRIRR,arRIRR,fioRIRR)
## Estimate standard errors from the models
b <- c(-1.687,-.873,-.055,.018,.456)
se <- c(1.369,.789,.808,.061,.351)
seRIRRs <- (RIRRs*se)/b
## Results
table <- as.data.frame(cbind(b,RIRRs,seRIRRs^2))
names(table) <- c("B (OLS)","log RIRR","Variance log RIRR")
rownames(table) <- c("Dispatched","OfficerInitiated",
                     "CrimeIncidents","ArrestReports","FIO")
pander(table)
```

	B (OLS)	log RIRR	Variance log RIRR
**Dispatched**	-1.687	-0.09052	0.005396
**OfficerInitiated**	-0.873	-0.09479	0.007339
**CrimeIncidents**	-0.055	0.00922	0.01835
**ArrestReports**	0.018	0.01317	0.001992
**FIO**	0.456	0.08431	0.004211

S627: Koslicki et al. (2019) Northwest City

There is one reference associated with this study.

- R627: Koslicki, W.M., Makin, D.A., & Willits, D. (2019). When no one is watching: Evaluating the impact of body-worn cameras on use of force incidents. *Policing and Society*, doi:10.1080/10439463.2019.1576672.

The one outcome, use of force, is analyzed using an OLS based interrupted time series method, accounting for autocorrelation. Unfortunately, there is no single coefficient associated with the treatment effect (they estimated the immediate change at the start of the intervention and the slope during the intervention period). They reported the means needed for Method 2, using the baseline as the control and post as the treatment. Using PlotDigitizer, we obtained recovered approximate data from Figure 1 in Koslicki et al. (2019). The purpose of using this method was to obtain the standard deviations for estimating over-dispersion. The means for these data are close to those reported by Koslicki et al. These means were not used but suggest that the

digitized data are approximately close to the actual values. The standard deviations on these data were used.

```
month <- c(seq(1:89))
prepost <- c(rep(0,52),rep(1,37))
y <- c(7.042514,11.03512,2.9944546,4.990758,9.066544,
      7.042514,2.0240295,7.014787,2.9944546,9.038817,5.046211,
      3.9926064,15,8.040666,0.9981516,7.042514,3.022181,
      1.025878,0,6.044362,4.990758,9.038817,12.033272,
      8.040666,2.9944546,3.9926064,4.990758,15.027726,
      5.046211,0,3.022181,12.033272,14.029574,13.031424,
      9.066544,1.9963032,6.044362,2.0240295,14.029574,
      6.044362,1.025878,4.020333,0,7.042514,7.014787,
      6.016636,5.018484,1.025878,3.9926064,0.9981516,7.014787,
      6.016636,0,0.9981516,3.022181,0.9704251,1.9963032,
      1.9685767,2.9944546,2.0240295,9.066544,11.062846,0,
      4.020333,3.9926064,1.025878,3.022181,9.038817,6.016636,
      3.022181,5.046211,4.020333,9.038817,3.022181,
      3.9926064,10.036968,1.9963032,5.018484,0,7.042514,
      10.036968,7.042514,0.9981516,2.9944546,3.9926064,
      11.03512,8.040666,5.046211,3.9926064)
data <- as.data.frame(cbind(month,prepost,y))
table <- aggregate(y ~ prepost, data=data, function(x) {c("Mean" = mean(x),
                                                           "SD" = sd(x))})
print(table)

##   prepost   y.Mean     y.SD
## 1       0 5.963849 4.055043
## 2       1 4.503672 3.280490
```

S633: Yokum et al. (2019) Washington, DC

There is one reference associated with this study.

- Yokum, D., Ravishankar, A., & Coppock, A. (2019). A randomized control trial evaluating the effects of police body-worn cameras. *Proceedings of the National Academy of Sciences, 116(21)*, 10329-10332.

Yokum and colleagues provided us a copy of these data but they are now available online at DC Body-Worn Camera Evaluation. These data have been anonymized and therefore do not have officer level covariates. Below, we convert the data from the wide format (one record per office) to the long format (two records per officer, one pre and one post). We also select variables of interest. There are five quasi-Poisson models that we run. The regression coefficient of interest is the time (PrePost) by treatment (Z) interaction, a difference-in-difference analysis (logged RIRR). The unit-of-analysis is the individual officer.

```
## Load needed libraries
library("MASS")
library("nlme")
library("dplyr")
```

```
##
## Attaching package: 'dplyr'

## The following object is masked from 'package:nlme':
##
##     collapse

## The following object is masked from 'package:MASS':
##
##     select

## The following objects are masked from 'package:stats':
##
##     filter, lag

## The following objects are masked from 'package:base':
##
##     intersect, setdiff, setequal, union

library("reshape")

##
## Attaching package: 'reshape'

## The following object is masked from 'package:dplyr':
##
##     rename

## The following objects are masked from 'package:tidyr':
##
##     expand, smiths

library("coefplot")

## Loading required package: ggplot2

library("estimatr")
library("knitr")
library("kableExtra")

##
## Attaching package: 'kableExtra'

## The following object is masked from 'package:dplyr':
##
##     group_rows

## set working director
setwd("~/new/bwc/search/pdfs/R633_data")
## Load anonymized dataset
officer_level <- read.csv("officer_level_anon.csv",
                          stringsAsFactors = FALSE)
## Drop specialty units (as per Yokum's analysis)
officer_level <- subset(officer_level,
                        district!="NSID" & district!="NSID_2" &
                          district!="SOD" & district!="SOD" &
                          district!="SSD" & district!="1Ds")
## Select Variables and Reshape to Long Format
```

```r
df <- officer_level %>%
      select(Z, block_id, district, district_block_id,
       weights, ID_anon,
       all_complaints1 =        all_complaints_pre,
       all_complaints2 =       all_complaints_post,
       all_complaints_1000_rate1 = all_complaints_1000_rate_pre,
       all_complaints_1000_rate2 = all_complaints_1000_rate_post,
       use_of_force1 =          use_of_force_pre,
       use_of_force2 =         use_of_force_post,
       use_of_force_1000_rate1 =  use_of_force_1000_rate_pre,
       use_of_force_1000_rate2 = use_of_force_1000_rate_post,
       disorderly_conduct1 =    disorderly_conduct_pre,
       disorderly_conduct2 =   disorderly_conduct_post,
       simple_assault1 =        simple_assault_pre,
       simple_assault2 =       simple_assault_post,
       traffic_arrest1 =        traffic_arrest_pre,
       traffic_arrest2 =       traffic_arrest_post,
       tickets1 =               tickets_pre,
       tickets2 =              tickets_post,
       warnings1 =              warnings_pre,
       warnings2 =              warnings_post,
       dv_arrests1 =             dv_arrests_pre,
       dv_arrests2 =             dv_arrests_post,
       dv_report_taken1 =       dv_report_taken_pre,
       dv_report_taken2 =       dv_report_taken_post,
       assault_on_po1   =       assault_on_po_pre,
       assault_on_po2   =       assault_on_po_post,
    )
dflong <- reshape(df, varying=c("all_complaints1",
                             "all_complaints2",
                             "all_complaints_1000_rate1",
                             "all_complaints_1000_rate2",
                             "use_of_force1", "use_of_force2",
                             "use_of_force_1000_rate1",
                             "use_of_force_1000_rate2",
                             "disorderly_conduct1",
                             "disorderly_conduct2",
                             "simple_assault1",
                             "simple_assault2",
                             "traffic_arrest1",
                             "traffic_arrest2",
                             "tickets1", "tickets2",
                             "warnings1", "warnings2",
                             "dv_arrests1",
                             "dv_arrests2",
                             "dv_report_taken1",
                             "dv_report_taken2",
                             "assault_on_po1",
                             "assault_on_po2"),
                 direction="long", timevar="PrePost",
```

```
                idvar=c("Z", "block_id", "district",
                        "district_block_id",
                        "weights", "ID_anon"),
                sep="")
rownames(dflong) <- NULL
## create dataframe with just those issuing
## traffic tickets and warnings
df_traffic <- subset(df, tickets1>0 & tickets2>0 &
                       warnings1>0 & warnings2>0,
                     select=c("Z", "block_id", "district",
                              "district_block_id",
                              "weights","ID_anon",
                              "tickets1","tickets2",
                              "warnings1","warnings2"))
dflong_traffic <- reshape(df_traffic, varying=c("tickets1",
                                              "tickets2",
                                              "warnings1",
                                              "warnings2"),
                direction="long", timevar="PrePost",
                idvar=c("Z", "block_id", "district",
                        "district_block_id",
                        "weights", "ID_anon"),
                sep="")
## combine: disorderly, simple, traffic
## combine: tickets, warnings
dflong$arrests <- dflong$disorderly_conduct +
                  dflong$simple_assault +
                  dflong$traffic_arrest
dflong_traffic$traffic <- dflong_traffic$warnings +
                          dflong_traffic$tickets
```

The table below shows the mean counts per officer by condition and time for each of the outcomes of interest.

```
means <- aggregate(cbind(all_complaints, use_of_force, dv_arrests,
                         dv_report_taken, assault_on_po, arrests)
                   ~ PrePost+Z, data=dflong, mean)
sds   <- aggregate(cbind(all_complaints, use_of_force, dv_arrests,
                         dv_report_taken, assault_on_po, arrests)
                   ~ PrePost+Z, data=dflong, sd)
means7 <- aggregate(traffic ~ PrePost+Z, data=dflong_traffic, mean)
sds7   <- aggregate(traffic ~ PrePost+Z, data=dflong_traffic, sd)
means <- cbind(means,means7)
sds   <- cbind(sds,sds7)
means <- t(round(means,3))[ c(3:8,11), ]
sds <- t(round(sds,3))[ c(3:8,11), ]
control_ns <- c(rep(table(df$Z)[[1]],6),table(df_traffic$Z)[[1]])
tx_ns      <- c(rep(table(df$Z)[[2]],6),table(df_traffic$Z)[[2]])
means <- cbind(means,control_ns,tx_ns)
sds   <- cbind(sds,control_ns,tx_ns)
colnames(means) <- c("Control/Pre","Control/Post","Tx/Pre","Tx/Post","Control
```

```
N","BWC N")
colnames(sds) <- c("Control/Pre","Control/Post","Tx/Pre","Tx/Post","Control N"
,"BWC N")
pander(means)
```

Table continues below

	Control/Pre	Control/Post	Tx/Pre	Tx/Post
**all_complaints**	0.225	0.162	0.209	0.195
**use_of_force**	0.409	0.471	0.33	0.515
**dv_arrests**	2.284	2.36	2.276	2.284
**dv_report_taken**	0	143.1	0	120.6
**assault_on_po**	0.575	0.782	0.43	0.853
**arrests**	4.972	9.078	4.604	9.31
**traffic**	76.85	69.04	49.71	64.8

	Control N	BWC N
**all_complaints**	888	1034
**use_of_force**	888	1034
**dv_arrests**	888	1034
**dv_report_taken**	888	1034
**assault_on_po**	888	1034
**arrests**	888	1034
**traffic**	130	156

```
pander(sds)
```

Table continues below

	Control/Pre	Control/Post	Tx/Pre	Tx/Post
**all_complaints**	0.573	0.49	0.591	0.527
**use_of_force**	0.945	1.05	0.721	1.204
**dv_arrests**	4.474	5.532	4.398	5.638
**dv_report_taken**	0	142.2	0	132.3
**assault_on_po**	1.544	1.785	1.247	1.853
**arrests**	9.322	13.42	7.861	13.08
**traffic**	212.7	194.4	137.1	240.5

	Control N	BWC N
**all_complaints**	888	1034
**use_of_force**	888	1034
**dv_arrests**	888	1034
**dv_report_taken**	888	1034
**assault_on_po**	888	1034

**arrests**	888	1034
**traffic**	130	156

This section estimates the logged relative incident rate ratios with standard errors adjusted for overdispersion using quasi-Poisson models. These models are difference-in-differences models that use both the *pre* and *post* counts. The treatment effect is the interaction between time and condition. Note that for the outcome *dv_report_taken*, there was no baseline (*pre*) data, so the treatment effect is a logged incident rate ratio between treatment and control during the *post* period. Also note that for *traffic_stops*, only those officers with non-zero counts both pre and post were included. These models do not take into account that the data represent repeated measures on the officers. However, the significance levels are roughly similar to the above models for *complaints* and *use of force*.

```
dvs <- c("all_complaints", "use_of_force", "dv_arrests",
         "dv_report_taken","assault_on_po", "arrests")
models1 <- lapply(dvs, function(x) {
  glm(substitute(i ~ Z*PrePost + factor(district),
                 list(i = as.name(x))), data = dflong,
      weights=weights, family=quasipoisson(link="log"))})
## traffic only has traffic officers
models1_7 <- glm(traffic ~ Z*PrePost + factor(district),
                 data = dflong_traffic,
                 weights=weights, family=quasipoisson(link="log"))
## dv_report_taken has no baseline (pre) data
models1_4 <- glm(dv_report_taken2  ~ Z + factor(district),
                 data = df,
                 weights=weights, family=quasipoisson(link="log"))
estimates1  <- unlist(lapply(seq(1:6), function(x)
   {summary(models1[[x]])$coefficients[[10,1]]} ))
std.errors1 <- unlist(lapply(seq(1:6), function(x)
   {summary(models1[[x]])$coefficients[[10,2]]} ))
estimates1  <- c(estimates1,  summary(models1_7)$coefficients[[10,1]])
std.errors1 <- c(std.errors1, summary(models1_7)$coefficients[[10,2]])
estimates1[4]  <- summary(models1_4)$coefficients[[2,1]]
std.errors1[4] <- summary(models1_4)$coefficients[[2,2]]
control_ns <- c(rep(table(df$Z)[[1]],6),table(df_traffic$Z)[[1]])
tx_ns      <- c(rep(table(df$Z)[[2]],6),table(df_traffic$Z)[[2]])
results1 <- cbind(estimates1,std.errors1^2,control_ns,tx_ns)
rownames(results1) <- c(dvs,"traffic")
colnames(results1) <- c("Estimate","Variance","Control N", "BWC N")
pander(results1)
```

	Estimate	Variance	Control N	BWC N
**all_complaints**	0.2841	0.03221	888	1034
**use_of_force**	0.3523	0.02069	888	1034
**dv_arrests**	-0.06713	0.0162	888	1034
**dv_report_taken**	-0.04188	0.001403	888	1034

**assault_on_po**	0.3048	0.02502	888	1034
**arrests**	0.09696	0.01091	888	1034
**traffic**	0.3412	0.2008	130	156

S637: Bennett et al. (2019) Fairfax County, VA

There is one reference associated with this study.

- R637: Bennett, R.R., Bartholomew, B., & Champagne, H. (2019). *Fairfax County Police Department's Body-Worn Camera Pilot Project: An Evaluation.* Washington, DC: Department of Justice, Law and Criminology, American University. Retrieved from https://www.fairfaxcounty.gov/police/sites/police/files/assets/documents/fcpd%20final%20report%2006_25_19.pdf

We extracted four effect sizes from this study. The study reported the results from ARIMA models. To get the logged RIRR that we wanted, we re-analyzed the data using a quasi-Poisson model. For two of the four outcomes, we could easily determine each value of the time series. For two of the time series, this was not possible. The authors kindly provided us with these data. The re-analysis to get our desired effect sizes is below.

```
## Figure 3.5 (complaints)
complaints_tx <- c(1,0,1,1,0,8,0,4,0,4,6,8,2,2,5,4,1,5,4,0,
                   3,5,4,1,0,0,1,1,7,0,2,3,7,0,0,1,2,1,1,1)
complaints_cg <- c(1,2,0,3,0,2,3,0,1,2,2,2,1,1,1,1,3,3,0,0,
                   2,0,0,1,4,2,1,3,2,0,3,0,0,1,4,0,4,1,0,0)
complaints <- c(complaints_tx, complaints_cg)
## Figure 3.6 (Use of Force)
force_tx  <- c(8, 8, 14, 9, 8, 11, 12, 15, 1, 1, 5, 0, 12, 2, 6, 9,
               12, 4, 10, 7, 8, 11, 9, 17, 17, 14, 8, 4, 23, 10, 7,
               0, 18, 13, 4, 3, 4, 7, 6, 0)
force_cg  <- c(3, 23, 24, 7, 7, 3, 3, 6, 6, 6, 0, 4, 11, 5, 1, 2, 4,
               11, 7, 10, 6, 4, 1, 15, 4, 12, 8, 14, 1, 3, 10, 8, 2,
               1, 11, 5, 8, 13, 3, 1)
force   <- c(force_tx,force_cg)
## Figure 3.1 Traffic Stops (provided by Bennett)
traffic_tx <- c(648, 136, 712, 338, 520, 310, 429, 201, 446, 253, 350,
                242, 352, 263, 464, 317, 435, 299, 524, 299, 326, 235,
                357, 257, 330, 255, 331, 284, 426, 269, 556, 359, 577,
                301, 498, 296, 477, 287, 488, 314, 369, 405, 382, 213,
                488, 474, 717, 351, 486, 364, 518, 305, 423, 407, 433,
                348, 634, 366, 524, 283, 479, 327, 310, 356, 495, 364,
                672, 383, 659, 387, 527, 327, 365, 335, 509, 343, 590,
                397, 513, 312, 497, 232, 487, 325, 452, 223, 412, 191,
                410, 319, 603, 331, 697, 378, 491)
traffic_cg <- c(217, 492, 248, 662, 424, 368, 338, 466, 254, 324, 311,
                396, 242, 377, 364, 429, 348, 398, 359, 514, 325, 330,
                271, 392, 310, 404, 301, 408, 304, 339, 354, 417, 385,
                555, 316, 323, 325, 397, 312, 439, 312, 456, 208, 321,
                324, 451, 358, 449, 381, 407, 299, 414, 426, 446, 268,
```

```
                436, 426, 647, 334, 451, 350, 487, 271, 436, 330, 367,
                447, 537, 333, 419, 401, 495, 350, 393, 337, 341, 296,
                496, 357, 385, 307, 367, 336, 452, 355, 430, 271, 339,
                217, 383, 285, 327, 301, 311, 174)
traffic <- c(traffic_tx,traffic_cg)
## Figure 3.2 Incidents Responded to by the Police (provided by Bennett)
incidents_tx <- c(327, 205, 296, 257, 312, 237, 340, 210, 305, 229, 254,
                  230, 296, 250, 300, 270, 331, 275, 334, 265, 292, 239,
                  349, 275, 326, 273, 352, 213, 294, 283, 306, 237, 273,
                  207, 290, 226, 288, 212, 305, 222, 274, 348, 216, 185,
                  290, 210, 305, 251, 345, 252, 317, 213, 292, 192, 328,
                  231, 315, 222, 300, 181, 302, 241, 304, 236, 345, 221,
                  308, 259, 312, 238, 317, 233, 315, 243, 346, 250, 344,
                  255, 334, 201, 328, 193, 391, 271, 269, 207, 316, 219,
                  292, 236, 258, 221, 306, 253, 295)
incidents_cg <- c(249, 288, 263, 358, 268, 307, 302, 327, 277, 325, 323,
                  339, 306, 460, 344, 376, 322, 350, 305, 416, 288, 348,
                  284, 401, 339, 381, 278, 377, 276, 391, 255, 348, 310,
                  405, 201, 403, 279, 324, 288, 306, 258, 356, 237, 327,
                  236, 293, 225, 341, 250, 348, 271, 333, 234, 333, 261,
                  311, 275, 323, 254, 373, 255, 373, 260, 359, 240, 337,
                  287, 409, 257, 351, 258, 380, 261, 350, 298, 323, 255,
                  329, 243, 316, 235, 285, 270, 350, 232, 312, 271, 359,
                  241, 313, 240, 311, 258, 324, 199)
incidents <- c(incidents_tx,incidents_cg)

## Create independent variables and dataframe for  complaints and force
week <- c(seq(7,46,by=1))
week <- c(week,week)
prepost <- c(rep(0,20),rep(1,13),rep(0,7))
prepost <- c(prepost,prepost)
group <- c(rep(1,40),rep(0,40))
data_fig5_fig6 <- as.data.frame(cbind(group,prepost,week,complaints,force))
data_fig5_fig6$group <- factor(data_fig5_fig6$group,levels=c(1,0),
                    labels=c("Treatment","Control"))
data_fig5_fig6$prepost <- factor(data_fig5_fig6$prepost,levels=c(0,1),
                      labels=c("Pre","Post"))
## Drop post intervention data
data_fig5_fig6_thru40 <- subset(data_fig5_fig6, week<41)


## Create independent variables and dataframe of traffic and incidents
week <- c(seq(1:95),seq(1:95))
prepost <- c(rep(0,53),rep(1,25),rep(0,17))
prepost <- c(prepost,prepost)
group <- c(rep(1,95),rep(0,95))
data_fig1_fig2 <- as.data.frame(cbind(group,prepost,week,incidents,traffic))
data_fig1_fig2$group <- factor(data_fig1_fig2$group,levels=c(1,0),
                               labels=c("Treatment","Control"))
data_fig1_fig2$prepost <- factor(data_fig1_fig2$prepost,levels=c(0,1),
```

```r
                                  labels=c("Pre","Post"))
data_fig1_fig2_thru79 <- subset(data_fig1_fig2, week<80)
## quasi-Poisson models
mod_complaints <- glm(complaints ~ prepost*group + week, data=data_fig5_fig6_thru40,
                       family = quasipoisson(link = "log"))
mod_force <- glm(force ~ prepost*group + week, data=data_fig5_fig6_thru40,
                       family = quasipoisson(link = "log"))
mod_incidents <- glm(incidents ~ prepost*group + week, data=data_fig1_fig2_thru79,
                     family = quasipoisson(link = "log"))
mod_traffic   <- glm(traffic   ~ prepost*group + week, data=data_fig1_fig2_thru79,
                     family = quasipoisson(link = "log"))
## Print results
summary(mod_complaints)
```

```
##
## Call:
## glm(formula = complaints ~ prepost * group + week, family = quasipoisson(link = "lo
g"),
##     data = data_fig5_fig6_thru40)
##
## Deviance Residuals:
##     Min       1Q   Median       3Q      Max
## -2.3672  -1.6509  -0.3442   0.7681   2.6544
##
## Coefficients:
##                             Estimate Std. Error t value Pr(>|t|)
## (Intercept)                 0.941659   0.366570   2.569   0.0126 *
## prepostPost                -0.053385   0.402049  -0.133   0.8948
## groupControl               -0.658056   0.308718  -2.132   0.0369 *
## week                        0.002215   0.017985   0.123   0.9024
## prepostPost:groupControl  0.022067   0.500025   0.044   0.9649
## ---
## Signif. codes:  0 '***' 0.001 '**' 0.01 '*' 0.05 '.' 0.1 ' ' 1
##
## (Dispersion parameter for quasipoisson family taken to be 1.820924)
##
##     Null deviance: 148.66  on 67  degrees of freedom
## Residual deviance: 134.89  on 63  degrees of freedom
## AIC: NA
##
## Number of Fisher Scoring iterations: 5
```

```r
summary(mod_force)
```

```
##
## Call:
## glm(formula = force ~ prepost * group + week, family = quasipoisson(link = "log"),
##     data = data_fig5_fig6_thru40)
##
## Deviance Residuals:
##     Min       1Q   Median       3Q      Max
## -4.5198  -1.4041  -0.4116   1.3783   4.5674
##
## Coefficients:
##                            Estimate Std. Error t value Pr(>|t|)
## (Intercept)                 2.39010    0.28215   8.471 5.37e-12 ***
## prepostPost                 0.63657    0.31972   1.991   0.0508 .
## groupControl               -0.14818    0.22244  -0.666   0.5077
```

```
## week                      -0.01850    0.01431  -1.292   0.2009
## prepostPost:groupControl -0.35809    0.34519  -1.037   0.3035
## ---
## Signif. codes:  0 '***' 0.001 '**' 0.01 '*' 0.05 '.' 0.1 ' ' 1
##
## (Dispersion parameter for quasipoisson family taken to be 3.825859)
##
##     Null deviance: 282.44  on 67  degrees of freedom
## Residual deviance: 254.55  on 63  degrees of freedom
## AIC: NA
##
## Number of Fisher Scoring iterations: 5
```

```
summary(mod_incidents)
```

```
##
## Call:
## glm(formula = incidents ~ prepost * group + week, family = quasipoisson(link = "log
"),
##     data = data_fig1_fig2_thru79)
##
## Deviance Residuals:
##     Min       1Q   Median       3Q      Max
## -6.7045  -2.5882  -0.0218   2.0287   7.4732
##
## Coefficients:
##                            Estimate Std. Error t value Pr(>|t|)
## (Intercept)               5.6405700  0.0355382 158.718  < 2e-16 ***
## prepostPost               0.0324916  0.0559870   0.580    0.563
## groupControl              0.1338435  0.0328820   4.070  7.5e-05 ***
## week                     -0.0009350  0.0009456  -0.989    0.324
## prepostPost:groupControl -0.0135612  0.0586431  -0.231    0.817
## ---
## Signif. codes:  0 '***' 0.001 '**' 0.01 '*' 0.05 '.' 0.1 ' ' 1
##
## (Dispersion parameter for quasipoisson family taken to be 8.545517)
##
##     Null deviance: 1514.1  on 157  degrees of freedom
## Residual deviance: 1310.2  on 153  degrees of freedom
## AIC: NA
##
## Number of Fisher Scoring iterations: 4
```

```
summary(mod_traffic)
```

```
##
## Call:
## glm(formula = traffic ~ prepost * group + week, family = quasipoisson(link = "log")
,
##     data = data_fig1_fig2_thru79)
##
## Deviance Residuals:
##     Min       1Q   Median       3Q      Max
## -13.981   -3.592   -1.137    3.145   15.733
##
## Coefficients:
##                           Estimate Std. Error t value Pr(>|t|)
## (Intercept)               5.909826   0.056588 104.436   <2e-16 ***
## prepostPost               0.061048   0.085044   0.718    0.474
## groupControl             -0.042692   0.052942  -0.806    0.421
## week                      0.001505   0.001507   0.999    0.320
```

```
## prepostPost:groupControl -0.026764   0.090871  -0.295    0.769
## ---
## Signif. codes:  0 '***' 0.001 '**' 0.01 '*' 0.05 '.' 0.1 ' ' 1
##
## (Dispersion parameter for quasipoisson family taken to be 28.48526)
##
##     Null deviance: 4421.5  on 157  degrees of freedom
## Residual deviance: 4198.8  on 153  degrees of freedom
## AIC: NA
##
## Number of Fisher Scoring iterations: 4
```
