# Supplementary material for: Body‐worn cameras’ effects on police officers and citizen behavior: A systematic review
Source: Campbell Syst Rev. 2020 Sep 9;16(3):e1112. doi: 10.1002/cl2.1112 (PMC8356344; doi:10.1002/cl2.1112)
Supplement: Supplementary file 5 — Supporting information [file CL2-16-e1112-s001.pdf]

APPENDIX F: SCRIPT FILE (R CODE) FOR ALL ANALYSES

Part of: Lum, C., Koper, C.S., Wilson, D.B., …et al. (2020). Body-worn cameras' effects on police officers and citizen behavior: A systematic review. Campbell Systematic Reviews 2020;e1112. https://doi.org/10.1002/cl2.1112.

This appendix includes the R script file for all analyses reported in this review, including code for generating all tables.

```
##
## BWC Meta-analysis
## R Code to Read Data from MySQL server
## and run analyses
## Created by D.B.Wilson
##
##

##-------------------------------------------------------------------
##
## Load libraries
##
##-------------------------------------------------------------------
library('RMySQL')
library('DBI')
library("tidyverse")
library("dplyr")
library("knitr")
library("kableExtra")
library("metafor")
library("janitor")
library("data.table")
library("tools")
library("stringr")
library("lubridate")
library("svglite")
options(width=200,scipen=999)

##-------------------------------------------------------------------
##
## Read data from database stored on Amazon AWS Server
##
##-------------------------------------------------------------------
## The data is stored on an Amazon AWS Server running MySQL database
## server (actually mariaDB, an opensource version of MySQL). The code
## below requires a password which is not provided.

## Create connection
bwc_db <- dbConnect(MySQL(), user='david', password='*****',
                 dbname='bwc_db',
                 host='ec2-52-14-126-221.us-east-2.compute.amazonaws.com')
## Read data tables
study       <- dbReadTable(conn = bwc_db, name = '2_Study')
outcome     <- dbReadTable(conn = bwc_db, name = '3_Outcome')
effectsize  <- dbReadTable(conn = bwc_db, name = '4_EffectSize')
## Disconnect from MySQL server
```

```
dbDisconnect(bwc_db)

##-------------------------------------------------------------------
##
## Drop S613, UK Rail study based on peer reviewer feedback
## and further consideration of consistentcy with eligibility
## criteria
##
##-------------------------------------------------------------------
study      <- subset(study, StudyID!="S613")
outcome    <- subset(outcome, StudyID!="S613")
effectsize <- subset(effectsize, StudyID!="S613")


##-------------------------------------------------------------------
##
## Save Data for Future Replicability
##
##-------------------------------------------------------------------
## Code saves the three dataframes in R's native data format in a
## single file prior to any manipuations. They can be read into R
## using the "load()" command as shwon below (commented out).
#setwd("~/new/bwc/data/R/")
save(study, outcome, effectsize, file="bwc_data.Rdata")
#load(file="bwc_data.Rdata")

##-------------------------------------------------------------------
##
## Study level data manipulations
##
##-------------------------------------------------------------------
## Create composite variable for publication type
study$PubType <- ifelse(study$PubType_J==1,"Journal/","")
study$PubType <-
ifelse(study$PubType_B==1,paste0(study$PubType,"Book/"),study$PubType)
study$PubType <- ifelse(study$PubType_BC==1,paste0(study$PubType,"Book
Chapter/"),study$PubType)
study$PubType <- ifelse(study$PubType_T1==1 |
                        study$PubType_T2==1,paste0(study$PubType,"Tech
Report/"),study$PubType)
study$PubType <-
ifelse(study$PubType_Dis==1,paste0(study$PubType,"Thesis/"),study$PubType)
study$PubType <-
ifelse(study$PubType_P==1,paste0(study$PubType,"Presentation/"),study$PubType)
study$PubType <-
ifelse(study$PubType_O==1,paste0(study$PubType,"Other/"),study$PubType)
study$PubType <- substr(study$PubType,1,nchar(study$PubType)-1)

##-------------------------------------------------------------------
##
## Outcome level data manipulations
##
##-------------------------------------------------------------------
outcome$Construct <- toTitleCase(outcome$Construct)
outcome$Construct <- factor(outcome$Construct)

##-------------------------------------------------------------------
##
## Functions for computing effect sizes and associated variance
## See Appendix XX for documentation and verification of these
## functions
```

```
##
##---------------------------------------------------------------------
##
## Method 1. Pre and post counts for treatment and control
## Method 2. Post counts only for treatment and control
## Method 3. Pre and post means and SDs for treatment and control
## Method 4. Post means and SDs for treatment and control
## Method 5. Post only dichotomous outcome data for treatment and control
## Method 6. Pre and post dichotomous outcome data for treatment and control
## Method 7. Regression models
##
## Method 1
logRIRRCounts <- function(T2,C2,T1,C1) {
    log((T2*C1)/(C2*T1))
}
logRIRRVCounts <-  function(T2,C2,T1,C1) {
     1/T2 + 1/T1 + 1/C2 + 1/C1
}
## Method 2
logIRRCountsPost <- function(T2,C2,TN,CN) {
     T2 <- ifelse(T2==0,(.5*TN)/(TN+CN),T2)
     C2 <- ifelse(C2==0,(.5*CN)/(TN+CN),C2)
     log((T2/TN)/(C2/CN))
}
logIRRVCountsPost <- function(T2,C2) {
    T2 <- ifelse(T2==0,.5,T2)
    C2 <- ifelse(C2==0,.5,C2)
    1/T2 + 1/C2
}
## Method 3
phi1 <- function(TM2,CM2,TM1,CM1,
                TS2,CS2,TS1,CS1,
                TN2,CN2,TN1,CN1) {
    sds <- c(TS2,CS2,TS1,CS1)
    ms <- c(TM2,CM2,TM1,CM1)
    ns <- c(TN2,CN2,TN1,CN1)
    d <- sum((sds^2*(ns-1)/ms)) * 1/(sum(ns)-4)
    d <- ifelse(d>1,d,1)
    return(d)
    }
logRIRRVOverD <- function(TM2,CM2,TM1,CM1,
                         TS2,CS2,TS1,CS1,
                         TN2,CN2,TN1,CN1) {
    v <- logRIRRVCounts(TM2*TN2,CM2*CN2,TM1*TN1,CM1*CN1)
    phi <- phi1(TM2,CM2,TM1,CM1,
               TS2,CS2,TS1,CS1,
               TN2,CN2,TN1,CN1)
    return(v*phi)
}
## Method 4
logIRRMeansPost <- function(TM2,CM2) {
     log((TM2)/(CM2))
}
phi2 <- function(TM2,CM2,TS2,CS2,TN2,CN2) {
    sds <- c(TS2,CS2)
    ms <- c(TM2,CM2)
    ns <- c(TN2,CN2)
    d <- sum((sds^2*(ns-1)/ms)) * 1/(sum(ns)-2)
    d <- ifelse(d>1,d,1)
    return(d)
    }
```

```
logIRRVOverDPost <- function(TM2,CM2,TS2,CS2,TN2,CN2) {
    v <- logIRRVCountsPost(TM2*TN2,CM2*CN2)
    d <- phi2(TM2,CM2,TS2,CS2,TN2,CN2)
    return(d*v)
}
## Method 5
logRRPostOnly <- function(TN2,CN2,T2,C2) {
    log((T2*CN2)/(C2*TN2))
}
logRRVPostOnly <- function(TN2,CN2,T2,C2) {
    (TN2 - T2)/(T2*TN2) +
    (CN2 - C2)/(C2*CN2)
}
## Method 6
logRRPrePost <- function(TN,CN,T2,C2,T1,C1) {
    log((T2 * CN)/(C2 * TN)) - log((T1 * CN)/(C1 * TN))
}
logRRVPrePost <- function(TN,CN,T2,C2,T1,C1) {
    (TN-T2)/(T2*TN) + (CN-C2)/(C2*CN) +
    (TN-T1)/(T1*TN) + (CN-C1)/(C1*CN)
}

## Method 7b
logRRlogOR <- function(lgOR,p1,p2) {
  OR <- exp(lgOR)
  psmall <- ifelse(p1<p2,p1,p2)
  plarge <- ifelse(p1>p2,p1,p2)
  p <- ifelse(OR<1, plarge, psmall)
  return(log(OR/((1-p)+(p*OR))))
}
logRRVlogOR  <- function(lgRR,lgOR,se) {
  ((lgRR*se)/lgOR)^2
}

## Method 7c
logRIRRolsB <- function(B,x) {
  log((x+B)/x)
}
logRIRRVolsB <- function(lgrirr,B,s) {
  ((lgrirr*s)/B)^2
}


##---------------------------------------------------------------------------
##
## Merge Study, Outcome, and Effect Size Data into One File call "es"
## Only keep "CL" and "CK" coding; drop doubling coding by "MS" and "MG"
##
##---------------------------------------------------------------------------
study <- subset(study, (CoderID=="CL" | CoderID=="CK"))
study$SubStudyID <- toupper(study$SubStudyID)
study <- rename(study, StudyTxN = TxN)
study <- rename(study, StudyCgN = CgN)
dv <- subset(outcome, CoderID=="CL" | CoderID=="CK")
dv$SubStudyID <- toupper(dv$SubStudyID)
es <- subset(effectsize, Coder=="CL" | Coder=="CK")
es$SubStudyID    <- toupper(es$SubStudyID)
## cleanup column order
es <- es[ ,c(1,2,3,4,5,6,7,8,9,10,11,12,13,14,15,
                 16,17,18,19,20,21,22,57,58,44,45,54,55,50,51,
                 23,24,25,26,46,47,48,49,27,28,42,43,29,
```

```
                 30,31,32,52,33,41,53,38,39,56,37,34,35,36,
                 40)]
## merge files
es <- full_join(es, dv,
                by=c("StudyID","SubStudyID","OutcomeID"),
                type='left', match='all')
es <- full_join(es, study,
                by=c("StudyID","SubStudyID"),
                type='left', match='all')
## create unique StudyID+SubStudyID
es$StudyIDSub <- paste0(es$StudyID,es$SubStudyID)
tabyl(es$StudyIDSub)
tabyl(es$Author)

##-------------------------------------------------------------------
##
## Compute effect sizes and associated variance
##
##-------------------------------------------------------------------
## determine which ES method to use
es <- es %>%
    dplyr::mutate(
        estype = case_when(
            !is.na(RegType) & grepl("Poisson",RegType) ~ "Method 7a" ,
            !is.na(RegType) & grepl("Logistic",RegType) ~ "Method 7b" ,
            !is.na(RegType) & grepl("OLS",RegType) ~ "Method 7c" ,
            !is.na(HandES) & !is.na(HandESV) ~ "Method 8" ,
            !is.na(TxMean) & !is.na(CgMean) &
            !is.na(TxSD) & !is.na(CgSD) &
            !is.na(TxMeanPre) & !is.na(CgMeanPre) &
            !is.na(TxSDPre) & !is.na(CgSDPre) ~ "Method 3" ,
            !is.na(TxMean) & !is.na(CgMean) &
            !is.na(TxSD) & !is.na(CgSD) &
            is.na(TxMeanPre) & is.na(CgMeanPre) &
            is.na(TxSDPre) & is.na(CgSDPre) ~ "Method 4" ,
            !is.na(TxFreq) & !is.na(CgFreq) &
            is.na(TxFreqPre) & is.na(CgFreqPre) ~ "Method 5" ,
            !is.na(TxFreq) & !is.na(CgFreq) &
            !is.na(TxFreqPre) & !is.na(CgFreqPre) ~ "Method 6" ,
            !is.na(TxProp) & !is.na(CgProp) &
            is.na(TxPropPre) & is.na(CgPropPre) ~ "Method 5" ,
            !is.na(TxProp) & !is.na(CgProp) &
            !is.na(TxPropPre) & !is.na(CgPropPre) ~ "Method 6" ,
            !is.na(TxCount) & !is.na(CgCount) &
            !is.na(TxCountPre) & !is.na(CgCountPre) ~ "Method 1" ,
            !is.na(TxCount) & !is.na(CgCount) &
            is.na(TxCountPre) & is.na(CgCountPre) ~ "Method 2" ,
            !is.na(TxMean) & is.na(CgMean) &
            !is.na(TxSD) & is.na(CgSD) &
            is.na(TxMeanPre) & !is.na(CgMeanPre) &
            is.na(TxSDPre) & !is.na(CgSDPre) ~ "Method 9" ,
            TRUE ~ "Unclassified (Error)"))
tabyl(es$estype)

## Break data apart by ES Method
es1 <- subset(es, estype=="Method 1")
es2 <- subset(es, estype=="Method 2")
es3 <- subset(es, estype=="Method 3")
es4 <- subset(es, estype=="Method 4")
es9 <- subset(es, estype=="Method 9") ## same as Method 4, just Cg is pre
es5 <- subset(es, estype=="Method 5")
```

```
es6 <- subset(es, estype=="Method 6")
es7a <- subset(es, estype=="Method 7a")
es7b <- subset(es, estype=="Method 7b")
es7c <- subset(es, estype=="Method 7c")
es8 <- subset(es, estype=="Method 8")

##
## Actually compute effect size
##
## Method 1
es1$lgrirr  <- with(es1, logRIRRCounts(TxCount,CgCount,TxCountPre,CgCountPre))
es1$vlgrirr <- with(es1, logRIRRVCounts(TxCount,CgCount,TxCountPre,CgCountPre))
## Method 2
es2$lgrirr  <- with(es2, logIRRCountsPost(TxCount,CgCount,TxN,CgN))
es2$vlgrirr <- with(es2, logIRRVCountsPost(TxCount,CgCount))
## Method 3
es3$lgrirr  <- with(es3, logRIRRCounts(TxMean,CgMean,TxMeanPre,CgMeanPre))
es3$vlgrirr <- with(es3, logRIRRVOverD(TxMean,CgMean,TxMeanPre,CgMeanPre,
                                       TxSD,CgSD,TxSDPre,CgSDPre,
                                       TxN,CgN,TxNPre,CgNPre))
es3$phi <- with(es3, phi1(TxMean,CgMean,TxMeanPre,CgMeanPre,
                                       TxSD,CgSD,TxSDPre,CgSDPre,
                                       TxN,CgN,TxNPre,CgNPre))
## Method 4 and 9
es4$lgrirr  <- with(es4, logIRRMeansPost(TxMean,CgMean))
es4$vlgrirr <- with(es4, logIRRVOverDPost(TxMean,CgMean,TxSD,CgSD,TxN,CgN))
es4$phi     <- with(es4, phi2(TxMean,CgMean,TxSD,CgSD,TxN,CgN))
es9$lgrirr  <- with(es9, logIRRMeansPost(TxMean,CgMeanPre))
es9$vlgrirr <- with(es9, logIRRVOverDPost(TxMean,CgMeanPre,
                                          TxSD,CgSDPre,TxN,CgN))
es9$phi     <- with(es9, phi2(TxMean,CgMean,TxSD,CgSD,TxN,CgN))
## Method 5
es5$TxFreq <- with(es5, ifelse(is.na(TxFreq),TxProp*TxN,TxFreq))
es5$CgFreq <- with(es5, ifelse(is.na(CgFreq),CgProp*CgN,CgFreq))
es5$lgrirr  <- with(es5, logRRPostOnly(TxN,CgN,TxFreq,CgFreq))
es5$vlgrirr <- with(es5, logRRVPostOnly(TxN,CgN,TxFreq,CgFreq))
## Method 6
es6$TxFreq    <- with(es6, ifelse(is.na(TxFreq),TxProp*TxN,TxFreq))
es6$CgFreq    <- with(es6, ifelse(is.na(CgFreq),CgProp*CgN,CgFreq))
es6$TxFreqPre <- with(es6, ifelse(is.na(TxFreqPre),TxPropPre*TxN,TxFreqPre))
es6$CgFreqPre <- with(es6, ifelse(is.na(CgFreqPre),CgPropPre*CgN,CgFreqPre))
es6$lgrirr  <- with(es6, logRRPrePost(TxN,CgN,TxFreq,CgFreq,
                                      TxFreqPre,CgFreqPre))
es6$vlgrirr <- with(es6, logRRVPrePost(TxN,CgN,TxFreq,CgFreq,
                                       TxFreqPre,CgFreqPre))
## Method 7a
es7a$lgrirr  <- es7a$BOLS
es7a$vlgrirr <- es7a$BSEOLS^2
## Method 7b
es7b$lgrirr <- with(es7b, logRRlogOR(BOLS, TxProp, CgProp))
es7b$vlgrirr <- with(es7b, logRRVlogOR(BOLS,lgrirr,BSEOLS))
## Method 7c
es7c$lgrirr  <- with(es7c, logRIRRolsB(BOLS, CgMean))
es7c$vlgrirr <- with(es7c, logRIRRVolsB(lgrirr,BOLS,BSEOLS))
es7c$lgrirr  <- ifelse(!is.na(es7c$HandES),es7c$HandES, es7c$lgrirr)
es7c$vlgrirr  <- ifelse(!is.na(es7c$HandESV),es7c$HandESV, es7c$vlgrirr)
## Method 8 (note that Cohen's d type effect sizes are dropped)
table(es8$HandType)
es8 <- subset(es8, HandType!="Cohen's d")
es8$lgrirr <- es8$HandES
es8$vlgrirr <- es8$HandESV
```

```
## put data back together
es <- bind_rows(es1,es2,es3,es4,es5,es6,es7a,es7b,es7c,es8,es9)
rm(es1,es2,es3,es4,es5,es6,es7a,es7b,es7c,es8,es9)
rm(list=ls(pattern="log"))
rm(phi1,phi2,effectsize)
## Compute Effect size Standard error and Weight
es$selgrirr <- sqrt(es$vlgrirr)
es$wlgrirr <- 1/es$vlgrirr

##------------------------------------------------------------------
##
## Data Cleanups
##
##------------------------------------------------------------------

## sort data frame
es <- es[ order(es$StudyID,es$SubStudyID,es$Construct,es$ESID), ]
es <- as.data.table(es)

## shorten direction labels
es$Direction <- ifelse(es$Direction=="Favors Treatment","Treatment",
                 ifelse(es$Direction=="Favors Control","Control","Neither"))
es$Direction <- factor(es$Direction)


##------------------------------------------------------------------
##
## Drop Redundant Effet Sizes
##
##------------------------------------------------------------------

##
## Drop redundant effect sizes: Method 1 and 3 for the same study and
## same outcome. In all cases, the effect size is the same (within rounding
## error) but method 3 is adjusted for over-dispersion.
##
## Similarly, method 2 and 7a have the same effect sizes within
## rounding error
##
## Others differ a bit by method but are roughly similar
##

## Determine which studies have two effect sizes for the
## same OutcomeID
es <- es %>%
    group_by(StudyIDSub,OutcomeID) %>%
    add_tally(name = "k")
table(es$StudyIDSub,es$k)
es <- as.data.frame(es)
print(es[ es$k>1,
                c("StudyIDSub","OutcomeID","ESID","lgrirr","vlgrirr","estype")])
## Select Method 3 and Method 1 over Method 6 (thus, drop method 6 if k>1
es <- subset(es, !(k>1 & estype=="Method 6"))
print(es[ es$k>1,
                c("StudyIDSub","OutcomeID","ESID","lgrirr","vlgrirr","estype")])
es$k <- NULL

##------------------------------------------------------------------
##
## Aggregate Multiple Remaining Effect Sizes within
```

```
## StudyIDSub/Construct
##
##------------------------------------------------------------------
##
## We first need to combine the two "Resistance" outcomes (S352 and S604)
## with the  "Officer Injuries/Assault on Officer" outcomes
##
tabyl(es$Construct)
es$Construct <- recode(es$Construct, "Resistance" = "Assault on Officer/Officer
Injuries/Resistance",
    "Officer Injuries/Assault on Officer" =  "Assault on Officer/Officer
Injuries/Resistance")
tabyl(es$Construct)

## Determine which studies have two effect sizes for the
## sample Construct (these are unique ESs but for the same construct)
es <- es %>%
    group_by(StudyIDSub,Construct) %>%
    add_tally(name = "k")
table(es$StudyIDSub,es$k)
es <- as.data.frame(es)
print(es[ es$k>1,
                c("StudyIDSub","OutcomeID","ESID","lgrirr","vlgrirr","estype")])
## S272A has two measures and two effect sizes for "complaints against officer"
## S612A has two measures and two effect sizes for "complaints against officer"
## S612A has two measures and two effect sizes for "use of force"
## S352A has two measures of Injuries/Assualts on Officers + Resistance
## Code below averages the effect size and variance and uses the average for
## the effect szie, dropping the duplicate record
esk1 <- subset(es, k==1)
esk2 <- subset(es, k==2)
esk2 <- esk2 %>%
    group_by(StudyIDSub,Construct) %>%
    mutate(mlgrirr = mean(lgrirr),
           mvlgrirr = mean(vlgrirr),
           rowID = row_number()) %>%
    ungroup
esk2$lgrirr  <- esk2$mlgrirr
esk2$vlgrirr <- esk2$mvlgrirr
esk2$mlgrirr  <- NULL
esk2$mvlgrirr <- NULL
esk2 <- subset(esk2, rowID == 1)
es.all <- es
es.one.per <- as.data.table(bind_rows(esk1,esk2))
es.one.per$rowID <- NULL
rm(esk1,esk2,es)

##------------------------------------------------------------------
##
## Ready to Run Analyses
##
##------------------------------------------------------------------

## summarize all study level variables
studyVars <- list("PubType", "LocationText", "LocationState",
               "LocationCountry", "PopDensity", "Diversity", "Agency",
               "AgencyType", "NumSworn", "ResTeam", "BWCPrior",
               "Reform", "BWCType", "BWCPolicy1", "DateBWCStarted",
               "DataStart", "DataEnd", "Fidelity", "UOA", "Design",
               "Sampling", "SamplingBias", "TxDesc", "CgDesc", "TxN",
               "CgN", "NumInvolved", "Attrition", "Power", "UOAOther",
```

```
                 "BWCTypeOther", "CriticalIncTypeOther", "Contaminate",
                 "BWCPolicy2", "BWCPolicy3", "DateBWCEnded",
                 "BWCPhasedIn", "BWCPhasedInYear", "RoB11",
                 "RoB1contam", "RoB13", "RoB1extra", "RoB1opt", "RoB26",
                 "RoB27", "RoB51", "RoB52", "RoB53")
names(studyVars) <- studyVars
#lapply(studyVars, function(x) {tabyl(study[[x]])} )

##
## Create Table of Study Level variables
##
## Fix dates
study$DataStartDate <- paste(month(mdy(study$DataStart), label = TRUE),
                             year(mdy(study$DataStart)), sep=" ")
study$DataEndDate   <- paste(month(mdy(study$DataEnd), label = TRUE),
                             year(mdy(study$DataEnd)), sep=" ")
## fix some things
study$BWCPolicy1 <- factor(study$BWCPolicy1, levels=c("Yes","No","Cannot tell"))
study$BWCPolicy3 <- factor(study$BWCPolicy3, levels=c("Yes","No","Not specified"))
study <- study %>%
    dplyr::mutate(
        discretion = case_when(
            word(BWCPolicy2, 1) == "cannot"   ~ "Cannot tell" ,
            word(BWCPolicy2, 1) == "higher"   ~ "Higher",
            word(BWCPolicy2, 1) == "moderate" ~ "Moderate",
            word(BWCPolicy2, 1) == "no"       ~ "No or low"))
study$discretion <- factor(study$discretion, levels=c("Higher","Moderate",
                                                      "No or low","Cannot tell"))
study_tbl1 <- subset(study, select=c(
                                AuthorLabel,
                                # ResTeam,
                                PopDensity,
                                BWCPhasedInYear,
                                Design,
                                UOA,
                                NumInvolved,
                                # NumSworn,
                                DataStartDate,
                                DataEndDate
                                #  BWCPolicy1,
                                # discretion,
                                #Reform
                             ))

study_tbl1$DataStartDate[ study_tbl1$DataStartDate=="NA NA"] <- ""
study_tbl1$DataEndDate[ study_tbl1$DataEndDate=="NA NA"] <- ""
study_tbl1$PopDensity <- format(as.numeric(study_tbl1$PopDensity), big.mark=",")
#study_tbl1$NumSworn <- format(study_tbl1$NumSworn, big.mark=",")
study_tbl1$NumInvolved <- format(study_tbl1$NumInvolved, big.mark=",")
study_tbl1 <- sapply(study_tbl1, as.character)
study_tbl1[is.na(study_tbl1)] <- ""
study_tbl1[study_tbl1=="    NA"] <- ""
row.names(study_tbl1) <- NULL
study_tbl1 <- study_tbl1[ order(study$AuthorLabel), ]
study_tbl1_html <- kable(study_tbl1,
                    caption="Study Level Descriptive Information",
                    format.args = list(big.mark = ","),
                    col.names=c("Study Name",
                              #  "Primary Research Organization",
                                "Population",
                                "Year of initial implementation of BWCs",
```

```
                                "Research Design",
                                "Unit-of-Assignment",
                              # "Total number of sworn officers",
                                "Number of officers involved in study",
                                "Intervention start date",
                                "Intervention end date"
#                   "BWC turned on by default for entire shift or for any police
encounter?",
#                   "Discretion to turn on/off?",
#                   "In the two years prior to camera adoption, agency underwent a
collaborative reform or sentinel event?"
                   ))
write_file(study_tbl1_html, "tables/study_tbl1.html")

##
## Study Summary Table 2
##
##study_tbl2 <- subset(study, select=c(
##                                AuthorLabel, Design, UOA,
##                                Sampling, ContaminateYN))
##study_tbl2 <- study_tbl2 %>%
##    dplyr::mutate(
##        sampling = case_when(
##            word(Sampling, 1) == "All"   ~ "All units used" ,
##            word(Sampling, 1) == "Convenience"   ~ "Convenience" ,
##            word(Sampling, 1) == "Hybrid"   ~ "Hybrid" ,
##            word(Sampling, 1) == "Specific"   ~ "Specific units",
##            TRUE ~ "Unknown"))
##study_tbl2$Sampling <- NULL
##study_tbl2 <- study_tbl2[ ,c(1:3,5,4)]
##study_tbl2_html <- kable(study_tbl2,
##                    caption="Study Level Design Information",
##                    col.names=c("Study Name",
##                                "Research Design",
##                                "Unit-of-Assignment",
##                                "How sample was selected",
##                                "Contamination of Control Condition"))
##write_file(study_tbl2_html, "tables/study_tbl2.html")

##
## Study Summary Table 3
##
## create grouped population variable
study$PopDensity <- as.numeric(study$PopDensity)
study <- study %>%
    dplyr::mutate(
        Population = case_when(
            PopDensity <   100000   ~ "<100,000" ,
            PopDensity <=  500000   ~ "100,000-500,000" ,
            PopDensity <= 1000000   ~ "500,001-1 million" ,
            PopDensity >  1000000   ~ "> 1 million" ,
            TRUE ~ "Unknown"))
study$Population <- factor(study$Population, levels=c("<100,000","100,000-500,000",
                                                      "500,001-1 million","> 1
million",
                                                      "Unknown"))
## extract just the year on when BWC implemented in agency
study$bwcyear <- as.numeric(substr(study$DateBWCStarted,
                                   nchar(study$DateBWCStarted)-3,
                                   nchar(study$DateBWCStarted)))
## create unit-of-analysis variable from ES level data
```

```
uoa <- as.data.table(unique(subset(es.one.per,
select=c("StudyID","SubStudyID","UOA.x"))))
uoa$geographic <- ifelse(uoa$UOA.x=="Geographic area","Geographic area & ","")
uoa$incident <- ifelse(uoa$UOA.x=="Incident","Incident & ","")
uoa$officer <- ifelse(uoa$UOA.x=="Officer","Officer & ","")
uoa$shift <- ifelse(uoa$UOA.x=="Shift","Shift & ","")
uoa$time <- ifelse(uoa$UOA.x=="Time period","Time period & ","")
uoa$SubStudyID[ uoa$StudyID=="S416"] <- "A" ## want to merge A & B for S416
uoa <- uoa[ , .(geographic = max(geographic), incident = max(incident), officer =
max(officer),
                shift = max(shift), time = max(time)), by = .(StudyID, SubStudyID)]
uoa$UOA <- with(uoa, paste0(geographic,incident,officer,shift,time))
uoa$UOA <- substr(uoa$UOA,1,nchar(uoa$UOA)-3)
## function to create summary subtables with frequency and percent by value
subTable <- function(var, label, sort) {
    subtbl <- as.data.frame(tabyl(var))[1:3]
    names(subtbl) <- c("Characteristic","N","Percent")
    subtbl$Characteristic[is.na(subtbl$Characteristic)] <- "Not reported"
    if(missing(sort)) {
        subtbl } else {
        subtbl <-  subtbl[ order(-subtbl$N), ]
               }
    subtbl$Characteristic <- as.character(subtbl$Characteristic)
    subtbl <- rbind(c(label,NA,NA),subtbl)
}
study_noS416B <- subset(study, !(StudyID=="S416" & SubStudyID=="B"))

subtbl.1  <- subTable(study_noS416B$PubType,         "Publication type", sort)
subtbl.2  <- subTable(study_noS416B$LocationCountry, "Country", sort)
subtbl.3  <- subTable(study_noS416B$ResTeam,         "Evaluation team", sort)
subtbl.4  <- subTable(study$Design,                  "Research design", sort)
subtbl.5  <- subTable(study_noS416B$Power,           "A priori power analysis")
subtbl.6  <- subTable(study_noS416B$Population,      "Population size")
subtbl.7  <- subTable(uoa$UOA,                       "Unit of analysis", sort)
subtbl.8  <- subTable(study_noS416B$BWCPrior,        "BWCs use by the agency prior to
the study", sort)
subtbl.9  <- subTable(study_noS416B$BWCType,         "Nature of BWC use during
intervention", sort)
subtbl.10  <- subTable(study_noS416B$BWCPolicy1,     "BWC turned on by default")
subtbl.11 <- subTable(study_noS416B$discretion,      "Discretion regarding on-off")
subtbl.12 <- subTable(study_noS416B$BWCPolicy3,      "Must inform citizen that BWC is
on")
subtbl.13 <- subTable(study_noS416B$Reform,          "In the two years prior to camera
adoption, agency underwent a collaborative reform or sentinel event?")
subtbl.14 <- subTable(study_noS416B$bwcyear,         "Year BWCs were first implmented
in agency")
subtbl.15 <- subTable(study_noS416B$ContaminateYN,  "Conamination of Control
Condition")
subtbl.16 <- subTable(study_noS416B$FidelityHL,     "Fidelity of BWC implementation")
study_tbl2 <- bind_rows(subtbl.2, subtbl.6, subtbl.4, subtbl.7, subtbl.3,
                    subtbl.1, subtbl.8, subtbl.14, subtbl.9, subtbl.10,
                    subtbl.11, subtbl.12, subtbl.13, subtbl.15,subtbl.16)
study_tbl2$Percent <- round(as.numeric(study_tbl2$Percent)*100,1)
rownames(study_tbl2) <- NULL
options(knitr.kable.NA = '')
study_tbl2_html <- kable(study_tbl2,
                    caption="Key Characteristics of Eligible Studies",
                    col.names=c("Characteristics","N","Percent"))
study_tbl2_html <- footnote(study_tbl2_html, general = "N = 31 except of Research
Design where N = 32. The White et al. (2018) and Wallace et al. (2016) are a site and
data collection but with two different designs and units-of-analysis")
```

```
write_file(study_tbl2_html, "tables/study_tbl2.html")


##
## Table showing which studies report which outcomes
##
study_tbl3 <- as.data.table(unique(subset(dv,
select=c("StudyID","SubStudyID","Construct"))))
study_tbl3$k <- 1
study_tbl3 <- spread(study_tbl3, Construct, k)
tmp_study <- subset(study, select=c("StudyID","SubStudyID","AuthorLabel"))
study_tbl3 <- full_join(study_tbl3, tmp_study,
                            by=c("StudyID","SubStudyID"),
                            type='left', match='all')
study_tbl3 <- study_tbl3[ order(study_tbl3$AuthorLabel) , c(16,3:15) ]
study_tbl3 <- sapply(study_tbl3, as.character)
study_tbl3[is.na(study_tbl3)] <- ""
study_tbl3[study_tbl3=="1"] <- "Yes"
study_tbl3_html <- kable(study_tbl3, caption="Outcome Constructs by Each Study for
Which an Effect Size was Computed")
write_file(study_tbl3_html, "tables/study_tbl3.html")

##
## Create table for study level risk-of-bias
##
study_tbl4 <- subset(study, select=c("AuthorLabel","RoB11","RoB13","RoB1extra",
                                    "RoB1contam","RoB1opt",
                                    "RoB26","RoB27","RoB51","RoB52","RoB53"))
study_tbl4[is.na(study_tbl4)] <- ""
study_tbl4[study_tbl4=="NA"] <- ""
column.labels <- c("Study Name","1.1","1.3","1 Extra","1 Cont.","1 Opts",
                   "2.6","2.7","5.1","5.2","5.3")
study_tbl4_html <- kable(study_tbl4,
                        caption="Study Level Risk-of-Bias (Slightly Modified Cochrane
Tool)",
                        col.names=c(column.labels))
study_tbl4_html <- add_header_above(study_tbl4_html,
    c("","Domain 1: Risk of bias arising from the randomization process" = 5,
      "Domain 2: Risk of bias due to deviations from the intended interventions" = 2,
      "Domain 5: Risk of bias in selection of the reported result" = 3))
study_tbl4_html <- footnote(study_tbl4_html,
                           general = c(
  "Y=Yes; PY=Probably Yes; PN=Probably No; N=No; NI=No information",
  "1.1: Was the allocation sequence random?",
  "1.3: Did baseline differences between intervention groups suggest a problem with
the randomization [or other selection] process?",
  "1 Extra: Were there violations to the randomization process?",
  "1 Cont.: Was there contamination between the treatment and control conditions?",
  "1 Opt.: What is the predicted direction of bias arising from the randomization [or
other selection] process?",
  "2.6: Was an appropriate analysis used to estimate the effect of assignment to
intervention?",
  "2.7: Was there potential for a substantial impact (on the result) of the failure to
analyze participants in the group to which they were randomized?",
  "5.1: Were the data that produced this result [the results for this study] analyzed
in accordance with a pre-specified analysis plan that was finalized before unblinded
outcome data were available for analysis?",
  "5.2: Is (Are) the numerical result [results] being assessed likely to have been
selected, on the basis of the results, from multiple outcome measurements?",
  "5.3: Is (Are) the numerical result [results] being assessed likely to have been
selected, on the basis of the results, from multiple analyses of the data?")
```

```
)
write_file(study_tbl4_html, "tables/study_tbl4.html")

##
## Risk of Bias, Outcome/DV level
##
dv_tbl1 <- subset(es.one.per, select=c("Construct","AuthorLabel",
                                      "RoB31","Rob32",
                                      "Rob42","Rob43","Rob44","Rob45"))
dv_tbl1[is.na(dv_tbl1)] <- ""
dv_tbl1[dv_tbl1=="NA"] <- ""
dv_tbl1 <- dv_tbl1[ order(dv_tbl1$Construct,dv_tbl1$AuthorLabel),]
column.labels <- c("Construct","Study Name",
    "3.1","3.2",
    "4.2","4.3","4.4","4.5")
dv_tbl1_html <- kable(dv_tbl1,
                        caption="Outcome/Dependent Variable Level Risk-of-Bias
(Slightly Modified Cochrane Tool)",
                        col.names=c(column.labels))
dv_tbl1_html <- add_header_above(dv_tbl1_html,
    c("","","Domain 3: Missing outcome data" = 2, "Domain 4: Risk of bias in
measurement" =  4))
dv_tbl1_html <- footnote(dv_tbl1_html,
                         general = c(
  "3.1: Were data for this outcome available for all, or nearly all, participants
randomized?",
  "3.2: If N/PN/NI to 3.1: Is there evidence that the result was not biased by missing
outcome data?",
  "4.2: Could measurement or ascertainment of the outcome have differed between
intervention groups?",
  "4.3: If N/PN/NI 4.2: Were outcome assessors aware of the intervention received by
study participants?",
  "4.4: If Y/PY/NI to 4.3: Could assessment of the outcome have been influenced by
knowledge of intervention received?",
  "4.5: If Y/PY/NI to 4.4: Is it likely that assessment of the outcome was influenced
by knowledge of intervention received?")
  )
write_file(dv_tbl1_html, "tables/dv_tbl1.html")

##
## Outcome/DV Risk of Bias, Summary Table
##
dv_tbl2 <- subset(dv_tbl1, Construct=="Complaints Against Officer" |
                          Construct=="Use of Force" |
                          Construct=="Arrests" |
                          Construct=="Assault on Officer/Officer Injuries/Resistance")

dv_tbl2$Construct <- factor(dv_tbl2$Construct)
dv_tbl2[dv_tbl2==""] <- "Not applicable"
rob31 <- as.data.frame.matrix(table(dv_tbl2$RoB31,dv_tbl2$Construct))
rob42 <- as.data.frame.matrix(table(dv_tbl2$Rob42,dv_tbl2$Construct))
rob43 <- as.data.frame.matrix(table(dv_tbl2$Rob43,dv_tbl2$Construct))
rob44 <- as.data.frame.matrix(table(dv_tbl2$Rob44,dv_tbl2$Construct))
rob45 <- as.data.frame.matrix(table(dv_tbl2$Rob45,dv_tbl2$Construct))
rob31   <- cbind(rob31,   round(prop.table(rob31)*100,1))
rob42   <- cbind(rob42,   round(prop.table(rob42)*100,1))
rob43   <- cbind(rob43,   round(prop.table(rob43)*100,1))
rob44   <- cbind(rob44,   round(prop.table(rob44)*100,1))
rob45   <- cbind(rob45,   round(prop.table(rob45)*100,1))
rob31$"Risk of Bias"   <- rownames(rob31)
rob42$"Risk of Bias"   <- rownames(rob42)
```

```
rob43$"Risk of Bias"   <- rownames(rob43)
rob44$"Risk of Bias"   <- rownames(rob44)
rob45$"Risk of Bias"   <- rownames(rob45)
rownames(rob31)   <- NULL
rownames(rob42)   <- NULL
rownames(rob43)   <- NULL
rownames(rob44)   <- NULL
rownames(rob45)   <- NULL
dv_tbl2 <- rbind(rob31,rob42, rob43, rob44, rob45)
dv_tbl2  <- rbind(
    c(NA,NA,NA,NA,NA,NA,NA,NA,"(3.1) Were data for this outcome available for all, or
nearly all, participants randomized?"),
    rob31,
    c(NA,NA,NA,NA,NA,NA,NA,NA,"(4.2) Could measurement or ascertainment of the outcome
have differed between intervention groups?"),
    rob42,
    c(NA,NA,NA,NA,NA,NA,NA,NA,"(4.3) If N/PN/NI 4.2: Were outcome assessors aware of
the intervention received by study participants?"),
    rob43,
    c(NA,NA,NA,NA,NA,NA,NA,NA,"(4.4) If Y/PY/NI to 4.3: Could assessment of the
outcome have been influenced by knowledge of intervention received?"),
    rob44,
    c(NA,NA,NA,NA,NA,NA,NA,NA,"(4.5) If Y/PY/NI to 4.4: Is it likely that assessment
of the outcome was influenced by knowledge of intervention received?"),
    rob45)
dv_tbl2 <- dv_tbl2[ ,c(9,1,5,2,6,3,7,4,8)]
dv_tbl2 <- sapply(dv_tbl2, as.character)
dv_tbl2[is.na(dv_tbl2)] <- ""
dv_tbl2_html <- kable(dv_tbl2,
                        caption="Frequencies and Percentages for Selected
Outcome/Dependent Variable
                               Risk-of-Bias Items",
                      col.names=c("Risk of Bias Question","N","Percent","N","Percent",
                                  "N","Percent","N","Percent"))
dv_tbl2_html <- add_header_above(dv_tbl2_html,
                                 c("",
                                   "Arrests" = 2,
                                   "Complaints against officer" = 2,
                                   "Assault on Officer/Officer Injuries/Resistance" =
2,
                                   "Use of Force" = 2))
write_file(dv_tbl2_html, "tables/dv_tbl2.html")

###
### Create tables for Appendix list each effect size and
### the data it is based on
###
study$StudyIDSub <- paste0(study$StudyID,study$SubStudyID)
appendix_tbl1 <- subset(study,select=c(StudyIDSub,AuthorLabel))
names(appendix_tbl1) <- c("StudyID","Author, Year, Location")
row.names(appendix_tbl1) <- NULL
appendix_tbl1_html <- kable(appendix_tbl1,
                            caption="Study Identifier and Study Author Labels",
                            align=c("l","l"))
write_file(appendix_tbl1_html, "tables/appendix_tbl1.html")

##
## Create table of Effect Sizes for Appendix
##
es.all <- es.all[ order(es.all$Construct,es.all$StudyID,es.all$SubStudyID), ]
appendix_tbl2 <- subset(es.all, select=c(StudyIDSub,Construct,estype,lgrirr,selgrirr))
```

```
row.names(appendix_tbl2) <- NULL
appendix_tbl2_html <- kable(appendix_tbl2,
                            caption="All Effect Sizes by Construct and Study 
Identifier",
                            align=c("l","l","l","r","r"),
                            col.names=c("Study ID","Construct",
                                        "Calculation Method",
                                        "log RIRR","SE log RIRR"))
write_file(appendix_tbl2_html, "tables/appendix_tbl2.html")

## Number of Effect Sizes by Construct
tabyl(es.one.per$Construct)

## ---------------------------------------------------------------------
##
## Main Meta-analyses and Results Tables and Plots
##
## ---------------------------------------------------------------------
## function used to generate and extract results to build tables
meanES <- function(construct) {
    rma.uni(lgrirr, vlgrirr,
            data=es.one.per[es.one.per$Construct==construct, ],
            method="REML", slab=AuthorLabel)
}
logRIRR2RIRR <- function(lgrirr) {
    ifelse(lgrirr<0,
          round(-(1-exp(lgrirr))*100,1) ,
    ifelse(lgrirr>0,
          round((exp(lgrirr)-1) * 100,1),
          0))
}
extractResults <- function(model) {
     c("Mean log RIRR" = logRIRR2RIRR(model$b),
       "Lower CI"      = logRIRR2RIRR(model$ci.lb),
       "Upper CI"      = logRIRR2RIRR(model$ci.ub),
       "k"             = round(model$k,0),
       "z"             = round(model$zval,3),
       "p of z"        = round(model$pval, 3),
       "Q"             = round(model$QE,3),
       "p of Q"        = round(model$QEp,3),
       "tau"           = round(sqrt(model$tau2),3)) }
##
## Mean ES and related stats by Construct
##
levels(es.one.per$Construct)[levels(es.one.per$Construct)=="Stop and Frisk"] <-
    "Field Interviews or Stop and Frisk"
levels(es.one.per$Construct)[levels(es.one.per$Construct)=="Traffic Stops"] <-
    "Traffic Stops or Traffic Tickets"
constructs <- as.list(levels(es.one.per$Construct))
names(constructs) <- levels(es.one.per$Construct)
es_tbl1_ma <- lapply(constructs, meanES)
es_tbl1_ma
es_tbl1    <- lapply(es_tbl1_ma, extractResults)
es_tbl1 <- matrix(unlist(es_tbl1,
                         recursive=FALSE, use.names=FALSE), ncol=9, byrow=TRUE)
es_tbl1 <- as.data.frame(es_tbl1)
names(es_tbl1) <- c("Mean % Change","Lower CI","Upper CI",
                    "k","z","p","Q","p of Q","tau")
names(constructs) <- constructs
rownames(es_tbl1) <- unlist(constructs)
es_tbl1$Construct <- row.names(es_tbl1)
```

```
es_tbl1 <- es_tbl1[ ,c(10,1:9)]
es_tbl1$Q[es_tbl1$k==1] <- NA
es_tbl1$"p of Q"[es_tbl1$k==1] <- NA
es_tbl1$tau[es_tbl1$k==1] <- NA
es_tbl1 <- es_tbl1[order(-es_tbl1$k),]
row.names(es_tbl1) <- NULL
es_tbl1_html <- kable(es_tbl1,
       caption="Overall Mean Percent Change by
                Construct and Associated Statistics",
       align=c("l","r","r","r","r","r","r","r","r","r"),
       col.names = c("Construct","Mean % Change","Lower CI","Upper CI",
                     "k","z","p","Q","p","tau"))
es_tbl1_html <-
    footnote(es_tbl1_html, general =
    c("Percent change based on Relative Incident Rate Ratio (RIRR). For values of RIRR
greater than 1, the percent change is RIRR minus 1. For values of RIRR less than one
it is 1 minus RIRR. A RIRR reflects the difference-in-difference percent change in
counts. Negative numbers reflect lower counts for the BWC condition."))
write_file(es_tbl1_html, "tables/es_tbl1.html")

##
## Forest plots
##
myforestPlot <- function(model,x,y) {
    par(mar=c(4,4,1,2))
    forest(model, xlab="Relative Incident Rate Ratio",
           transf=exp, refline=1, clim=c(0,y), annotate=FALSE,
           xlim=c(x, y), order=order(model$yi))
    text(x,  model$k+2, "Author(s)/Year/Location",  pos=4)
    text( 1,  model$k+2, "Favors Treatment", pos=2)
    text( 1,  model$k+2, "Favors Control", pos=4)
}
svglite("./figures/arrests.svg",  width=11,height=4.5)
myforestPlot(es_tbl1_ma$"Arrests",-4,3)
dev.off()
svglite("./figures/complaints.svg",  width=11,height=6)
myforestPlot(es_tbl1_ma$"Complaints Against Officer",-10,10)
dev.off()
svglite("./figures/dispatched.svg",  width=11,height=4)
myforestPlot(es_tbl1_ma$"Dispatched Calls for Service",-2,2)
dev.off()
svglite("./figures/incident.svg",  width=11,height=3)
myforestPlot(es_tbl1_ma$"Incident Reports",-2,2)
dev.off()
svglite("./figures/officercfs.svg",  width=11,height=4.5)
myforestPlot(es_tbl1_ma$"Officer Initiated CFS",-8,5)
dev.off()
svglite("./figures/assaults.svg",  width=11,height=4.5)
myforestPlot(es_tbl1_ma$"Assault on Officer/Officer Injuries/Resistance",-10,10)
dev.off()
svglite("./figures/stopfrisk.svg",  width=11,height=2.5)
myforestPlot(es_tbl1_ma$"Field Interviews or Stop and Frisk",-2,2)
dev.off()
svglite("./figures/stoptraffic.svg",  width=11,height=3)
myforestPlot(es_tbl1_ma$"Traffic Stops or Traffic Tickets",-2,2)
dev.off()
svglite("./figures/useofforce.svg",  width=11,height=6)
myforestPlot(es_tbl1_ma$"Use of Force",-3,4)
dev.off()
system("./figures/convertsvg2emf") ## convert svf files to emf files
```

```
## ---------------------------------------------------------------------
##
## Moderator Meta-analyses
##
## ---------------------------------------------------------------------

##
## Moderator analysis by Design
##
es.one.per <- es.one.per %>%
    dplyr::mutate(
               design = case_when(
                   substr(Design,1,3) == "RCT" ~ "RCT",
#                   substr(Design,5,8) == "time" ~ "TS",
                   TRUE ~ "QE" ))

es.one.per$design <- factor(es.one.per$design)
table(es.one.per$Construct,es.one.per$design)
design_k <- as.data.frame.matrix(table(es.one.per$Construct,es.one.per$design))
design_k$k <- apply(design_k[ ,c(1:2)], 1, sum)
design_k <- design_k[ design_k$k>2 ,]
constructs <- row.names(design_k)
design_k$Construct <- constructs
names(constructs) <- constructs
es_design_ma <- lapply(constructs, function(x) {
    rma.uni(lgrirr ~ design, vlgrirr, data=es.one.per[es.one.per$Construct==x, ],
            method="REML", slab=AuthorLabel) })
es_design_QB <- lapply(es_design_ma, function(x) { x$QM })
es_design_QBp <- lapply(es_design_ma, function(x) { x$QMp })
## Create Table of Results for Above Moderator Analysis
es_design_QB <-  as.data.frame(unlist(es_design_QB))
es_design_QBp <-  as.data.frame(unlist(es_design_QBp))
es_design_QB <- cbind(es_design_QB,es_design_QBp)
es_design_QB <- round(es_design_QB,3)
names(es_design_QB) <- c("QB","QBp")
es_design_QB$Construct <- row.names(es_design_QB)
## generated mean ES by design category
es_design <- lapply(es_design_ma, function(x) {
#     predict(x, newmods=matrix(c(0,0,1,0,0,1), nrow=3, byrow=TRUE))
       predict(x, newmods=c(0,1))
} )
es_design <- lapply(es_design, as.data.frame)
es_design <- bind_rows(es_design, .id = "Construct")
es_design <- es_design[ ,1:5]
es_design <- es_design[ , -3]
design_k <- gather(design_k, design, freq, "QE":"RCT")
design_k <- design_k[ order(design_k$Construct,design_k$design) , ]
design_k <- design_k[ design_k$freq>0, ]
design_k <- as.data.table(design_k)
design_k <- design_k[ , row := seq_len(.N), by = Construct]
es_design <- as.data.table(es_design)
es_design <- es_design[ , row := seq_len(.N), by = Construct]
es_design <- full_join(es_design, design_k)
es_design$rirr  <- logRIRR2RIRR(es_design$pred)
es_design$lower <- logRIRR2RIRR(es_design$ci.lb)
es_design$upper <- logRIRR2RIRR(es_design$ci.ub)
es_design  <- full_join(es_design,es_design_QB,
                        by=c("Construct"))
es_design <- es_design[ ,c(1,7,8,9:13,5)]
es_design$Construct[es_design$row>1] <- ""
es_design$QB[es_design$row>1] <- ""
```

```
es_design$QBp[es_design$row>1] <- ""
es_design$row <- NULL
row.names(es_design) <- NULL
es_design$QB <- as.numeric(es_design$QB)
es_design$QBp <- as.numeric(es_design$QBp)
es_design
options(knitr.kable.NA = '')
es_design_html <- kable(es_design,
       caption="Mean Percent Change Based by Design (RCTs vs. QEs) and Construct",
       align=c("l","l","r","r","r","r","r","r"),
       col.names = c("Construct","Design","k","Mean % Change",
                     "Lower CI","Upper CI","Q-Between","p (Q-Between)"),
       digits = c(0,0,0,1,1,1,3,3))
es_design_html <-
    footnote(es_design_html, general =
      c("Q-Between is the test of the difference between the design means."))
write_file(es_design_html, "tables/es_moderator_design.html")


## keep just the constructs we need for the reminaing moderator analyses
tmp <- es.one.per
es.one.per <- subset(es.one.per, (Construct=="Complaints Against Officer" |
                                        Construct=="Use of Force" |
                                        Construct=="Arrests" |
                                        Construct=="Assault on Officer/Officer
Injuries/Resistance"))
es.one.per$Construct <- factor(es.one.per$Construct)
tabyl(es.one.per$Construct)


##
## Moderator analysis by BWC Policy Discretion
##
tabyl(es.one.per$BWCPolicy2)
es.one.per <- es.one.per %>%
    dplyr::mutate(
        discretion = case_when(
            word(BWCPolicy2, 1) == "higher"   ~ "Higher",
            word(BWCPolicy2, 1) == "moderate" ~ "Moderate",
            word(BWCPolicy2, 1) == "no"       ~ "No or low",
            TRUE ~ ""))
es.one.per$discretion <- factor(es.one.per$discretion,
                               levels=c("Higher","Moderate", "No or low"))
tabyl(es.one.per$discretion)
table(es.one.per$Construct,es.one.per$discretion)
discretion_k <-
as.data.frame.matrix(table(es.one.per$Construct,es.one.per$discretion))
#discretion_k$k <- apply(discretion_k[ ,c(1:2)], 1, sum)
discretion_k$k <- apply(discretion_k[ ,c(1:3)], 1, sum)
discretion_k$Construct <- row.names(discretion_k)
constructs <- row.names(discretion_k)
names(constructs) <- constructs
es_discretion_ma <- lapply(constructs, function(x) {
    rma.uni(lgrirr ~ discretion, vlgrirr, data=es.one.per[es.one.per$Construct==x &

!is.na(es.one.per$discretion), ],
            method="REML", slab=AuthorLabel) })
## generated mean ES by discretion category
es_discretion <- lapply(es_discretion_ma, function(x) {
     predict(x, newmods=matrix(c(0,0,1,0,0,1), nrow=3, byrow=TRUE))
#    predict(x, newmods=c(0,1))
```

```
})
es_discretion <- lapply(es_discretion, as.data.frame)
es_discretion <- bind_rows(es_discretion, .id = "Construct")
es_discretion <- es_discretion[ ,1:5]
es_discretion <- es_discretion[ , -3]
#discretion_k <- gather(discretion_k, discretion, freq, "Moderate or higher":"No or
low")
discretion_k <- gather(discretion_k, discretion, freq, "Higher":"No or low")
#discretion_k$discretion <- factor(discretion_k$discretion, ordered = TRUE ,
#                                  labels=c("Moderate or higher","No or low"))
discretion_k$discretion <- factor(discretion_k$discretion, ordered = TRUE ,
                                  labels=c("Higher","Moderate","No or low"))
discretion_k <- discretion_k[ order(discretion_k$Construct,discretion_k$discretion) ,
]
discretion_k <- as.data.table(discretion_k)
discretion_k <- discretion_k[ , row := seq_len(.N), by = Construct]
es_discretion <- as.data.table(es_discretion)
es_discretion <- es_discretion[ , row := seq_len(.N), by = Construct]
es_discretion <- full_join(es_discretion, discretion_k)
es_discretion$rirr  <- logRIRR2RIRR(es_discretion$pred)
es_discretion$lower <- logRIRR2RIRR(es_discretion$ci.lb)
es_discretion$upper <- logRIRR2RIRR(es_discretion$ci.ub)
## linear regression moderator analysis
es.one.per <- es.one.per %>%
    dplyr::mutate(
        discretion_linear = case_when(
            word(BWCPolicy2, 1) == "higher"   ~ 3,
            word(BWCPolicy2, 1) == "moderate" ~ 2,
            word(BWCPolicy2, 1) == "no"       ~ 1))
table(es.one.per$discretion,es.one.per$discretion_linear)
es_discretion_ma <- lapply(constructs, function(x) {
    rma.uni(lgrirr ~ discretion_linear, vlgrirr,
data=es.one.per[es.one.per$Construct==x &

!is.na(es.one.per$discretion_linear), ],
            method="REML", slab=AuthorLabel) })
beta    <- as.data.frame(unlist(lapply(es_discretion_ma, function(x) { x$beta[2] })))
p       <- as.data.frame(unlist(lapply(es_discretion_ma, function(x) { x$pval[2] })))
z      <- as.data.frame(unlist(lapply(es_discretion_ma, function(x) { x$zval[2] })))
discretion_reg <- cbind(beta, z, p)
colnames(discretion_reg) <- c("b","z","p")
discretion_reg <- round(discretion_reg,3)
discretion_reg$Construct <- row.names(discretion_reg)
rownames(discretion_reg) <- NULL
discretion_reg
## merge the two tables
es_discretion  <- full_join(es_discretion,discretion_reg,
                        by=c("Construct"))
es_discretion$b[es_discretion$row>1] <- NA
es_discretion$z[es_discretion$row>1] <- NA
es_discretion$p[es_discretion$row>1] <- NA
es_discretion$Construct[es_discretion$row>1] <- NA
es_discretion <- es_discretion[ ,c(1,7,8,9:14)]
row.names(es_discretion) <- NULL
es_discretion_html <- kable(es_discretion,
       caption="Mean Percent Change Based by Discretion and Construct and Meta-
Regression for Linear Relationship between Discretion and logged RIRR",
       align=c("l","l","r","r","r","r","r","r","r"),
       col.names = c("Construct","Discretion","k","Mean % Change",
                     "Lower CI","Upper CI","Regression Coefficient","z","p"),
       digits = c(0,0,0,1,1,1,3,3,3))
```

```
#write_file(es_discretion_html, "tables/es_moderator_discretion.html")
write_file(es_discretion_html, "tables/es_moderator_discretion_3categories.html")

##
## Moderator analysis by Contamination
##
tabyl(es.one.per$ContaminateYN)
contaminate_k <-
as.data.frame.matrix(table(es.one.per$Construct,es.one.per$ContaminateYN))
contaminate_k$k <- apply(contaminate_k[ ,c(1:2)], 1, sum)
contaminate_k$Construct <- row.names(contaminate_k)
constructs <- row.names(contaminate_k)
names(constructs) <- constructs
es_contaminate_ma <- lapply(constructs, function(x) {
    rma.uni(lgrirr ~ ContaminateYN, vlgrirr, data=es.one.per[es.one.per$Construct==x,
],
            method="REML", slab=AuthorLabel) })
es_contaminate_QB <- lapply(es_contaminate_ma, function(x) { x$QM })
es_contaminate_QBp <- lapply(es_contaminate_ma, function(x) { x$QMp })
## Create Table of Results for Above Moderator Analysis
es_contaminate_QB <-  as.data.frame(unlist(es_contaminate_QB))
es_contaminate_QBp <-  as.data.frame(unlist(es_contaminate_QBp))
es_contaminate_QB <- cbind(es_contaminate_QB,es_contaminate_QBp)
es_contaminate_QB <- round(es_contaminate_QB,3)
names(es_contaminate_QB) <- c("QB","QBp")
es_contaminate_QB$Construct <- row.names(es_contaminate_QB)
## generated mean ES by contaminate category
es_contaminate <- lapply(es_contaminate_ma, function(x) {
       predict(x, newmods=c(0,1))
     } )
es_contaminate <- lapply(es_contaminate, as.data.frame)
es_contaminate <- bind_rows(es_contaminate, .id = "Construct")
es_contaminate <- es_contaminate[ ,1:5]
es_contaminate <- es_contaminate[ , -3]
contaminate_k <- gather(contaminate_k, contaminate, freq, "Less Likely":"More Likely")
contaminate_k <- contaminate_k[
order(contaminate_k$Construct,contaminate_k$contaminate) , ]
contaminate_k <- contaminate_k[ contaminate_k$freq>0, ]
contaminate_k <- as.data.table(contaminate_k)
contaminate_k <- contaminate_k[ , row := seq_len(.N), by = Construct]
es_contaminate <- as.data.table(es_contaminate)
es_contaminate <- es_contaminate[ , row := seq_len(.N), by = Construct]
es_contaminate <- full_join(es_contaminate, contaminate_k)
es_contaminate$rirr  <- logRIRR2RIRR(es_contaminate$pred)
es_contaminate$lower <- logRIRR2RIRR(es_contaminate$ci.lb)
es_contaminate$upper <- logRIRR2RIRR(es_contaminate$ci.ub)
es_contaminate  <- full_join(es_contaminate,es_contaminate_QB,
                        by=c("Construct"))
es_contaminate <- es_contaminate[ ,c(1,7,8,9:13,5)]
es_contaminate$Construct[es_contaminate$row>1] <- ""
es_contaminate$QB[es_contaminate$row>1] <- ""
es_contaminate$QBp[es_contaminate$row>1] <- ""
es_contaminate$row <- NULL
row.names(es_contaminate) <- NULL
es_contaminate$QB <- as.numeric(es_contaminate$QB)
es_contaminate$QBp <- as.numeric(es_contaminate$QBp)
es_contaminate_html <- kable(es_contaminate,
       caption="Mean Percent Change by Whether There are Contamination of the Control
Condition Concerns and Construct",
       align=c("l","l","r","r","r","r","r","r"),
       col.names = c("Construct","contaminate","k","Mean % Change",
```

```
                     "Lower CI","Upper CI","Q-Between","p (Q-Between)"),
       digits = c(0,0,0,1,1,1,3,3))
es_contaminate_html <-
    footnote(es_contaminate_html, general =
      c("Q-Between is the test of the difference between the means."))
write_file(es_contaminate_html, "tables/es_moderator_contaminate.html")

##
## Moderator analysis by Organization
## ASU vs. Cambridge vs. everyone else
##
tabyl(es.one.per$ResTeam)
es.one.per <- es.one.per %>%
    dplyr::mutate(
               university = case_when(
                   word(ResTeam, 1) == "Arizona"   ~ "ASU" ,
                   word(ResTeam, 3) == "Cambridge" ~ "Cambridge",
                   TRUE ~ "Other"))
tabyl(es.one.per$university)
es.one.per$university <- factor(es.one.per$university)
tabyl(es.one.per$university)
table(es.one.per$Construct,es.one.per$university)
university_k <-
as.data.frame.matrix(table(es.one.per$Construct,es.one.per$university))
university_k$k <- apply(university_k[ ,c(1:3)], 1, sum)
university_k$Construct <- row.names(university_k)
constructs <- row.names(university_k)
names(constructs) <- constructs
es_university_ma <- lapply(constructs, function(x) {
    rma.uni(lgrirr ~ university, vlgrirr, data=es.one.per[es.one.per$Construct==x, ],
            method="REML", slab=AuthorLabel) })
es_university_QB <- lapply(es_university_ma, function(x) { x$QM })
es_university_QBp <- lapply(es_university_ma, function(x) { x$QMp })
## Create Table of Results for Above Moderator Analysis
es_university_QB <-  as.data.frame(unlist(es_university_QB))
es_university_QBp <-  as.data.frame(unlist(es_university_QBp))
es_university_QB <- cbind(es_university_QB,es_university_QBp)
es_university_QB <- round(es_university_QB,3)
names(es_university_QB) <- c("QB","QBp")
es_university_QB$Construct <- row.names(es_university_QB)
## generated mean ES by university category
es_university <- lapply(es_university_ma, function(x) {
    predict(x, newmods=matrix(c(0,0,1,0,0,1), nrow=3, byrow=TRUE))
    } )
es_university <- lapply(es_university, as.data.frame)
es_university <- bind_rows(es_university, .id = "Construct")
es_university <- es_university[ ,1:5]
es_university <- es_university[ , -3]
university_k <- gather(university_k, university, freq, "ASU":"Other")
university_k$university <- factor(university_k$university, ordered = TRUE ,
                                  labels=c("ASU","Cambridge","Other"))
university_k <- university_k[ order(university_k$Construct,university_k$university) ,
]
university_k <- university_k[ university_k$freq>0, ]
university_k <- as.data.table(university_k)
university_k <- university_k[ , row := seq_len(.N), by = Construct]
es_university <- as.data.table(es_university)
es_university <- es_university[ , row := seq_len(.N), by = Construct]
es_university <- full_join(es_university, university_k)
es_university$rirr  <- logRIRR2RIRR(es_university$pred)
es_university$lower <- logRIRR2RIRR(es_university$ci.lb)
```

```
es_university$upper <- logRIRR2RIRR(es_university$ci.ub)
es_university  <- full_join(es_university,es_university_QB,
                        by=c("Construct"))
es_university <- es_university[ ,c(1,7,8,9:13,5)]
es_university$Construct[es_university$row>1] <- ""
es_university$QB[es_university$row>1] <- ""
es_university$QBp[es_university$row>1] <- ""
es_university$row <- NULL
row.names(es_university) <- NULL
es_university$QB <- as.numeric(es_university$QB)
es_university$QBp <- as.numeric(es_university$QBp)
es_university_html <- kable(es_university,
       caption="Mean Percent Change by Research Team and Construct",
       align=c("l","l","r","r","r","r","r","r"),
       col.names = c("Construct","university","k","Mean % Change",
                     "Lower CI","Upper CI","Q-Between","p (Q-Between)"),
       digits = c(0,0,0,1,1,1,3,3))
es_university_html <-
    footnote(es_university_html, general =
      c("Q-Between is the test of the difference among the means."))
write_file(es_university_html, "tables/es_moderator_university.html")

##
## Moderator analysis by Fidelity
##
es.one.per$FidelityHL <- factor(es.one.per$FidelityHL)
tabyl(es.one.per$FidelityHL)
table(es.one.per$Construct,es.one.per$FidelityHL)
FidelityHL_k <-
as.data.frame.matrix(table(es.one.per$Construct,es.one.per$FidelityHL))
FidelityHL_k$k <- apply(FidelityHL_k[ ,c(1:3)], 1, sum)
FidelityHL_k$Construct <- row.names(FidelityHL_k)
constructs <- row.names(FidelityHL_k)
names(constructs) <- constructs
es_FidelityHL_ma <- lapply(constructs, function(x) {
    rma.uni(lgrirr ~ FidelityHL, vlgrirr, data=es.one.per[es.one.per$Construct==x &
                                                        !is.na(es.one.per$FidelityHL),
],
            method="REML", slab=AuthorLabel) })
es_FidelityHL_QB <- lapply(es_FidelityHL_ma, function(x) { x$QM })
es_FidelityHL_QBp <- lapply(es_FidelityHL_ma, function(x) { x$QMp })
## Create Table of Results for Above Moderator Analysis
es_FidelityHL_QB <-  as.data.frame(unlist(es_FidelityHL_QB))
es_FidelityHL_QBp <-  as.data.frame(unlist(es_FidelityHL_QBp))
es_FidelityHL_QB <- cbind(es_FidelityHL_QB,es_FidelityHL_QBp)
es_FidelityHL_QB <- round(es_FidelityHL_QB,3)
names(es_FidelityHL_QB) <- c("QB","QBp")
es_FidelityHL_QB$Construct <- row.names(es_FidelityHL_QB)
## generated mean ES by FidelityHL category
es_FidelityHL <- lapply(es_FidelityHL_ma, function(x) {
    predict(x, newmods=matrix(c(0,0,1,0,0,1), nrow=3, byrow=TRUE))
    } )
es_FidelityHL <- lapply(es_FidelityHL, as.data.frame)
es_FidelityHL <- bind_rows(es_FidelityHL, .id = "Construct")
es_FidelityHL <- es_FidelityHL[ ,1:5]
es_FidelityHL <- es_FidelityHL[ , -3]
FidelityHL_k <- gather(FidelityHL_k, FidelityHL, freq, "Higher":"Unsure")
FidelityHL_k$FidelityHL <- factor(FidelityHL_k$FidelityHL, ordered = TRUE ,
                                  labels=c("Higher","Lower","Unsure"))
FidelityHL_k <- FidelityHL_k[ order(FidelityHL_k$Construct,FidelityHL_k$FidelityHL) ,
]
```

```
FidelityHL_k <- FidelityHL_k[ FidelityHL_k$freq>0, ]
FidelityHL_k <- as.data.table(FidelityHL_k)
FidelityHL_k <- FidelityHL_k[ , row := seq_len(.N), by = Construct]
es_FidelityHL <- as.data.table(es_FidelityHL)
es_FidelityHL <- es_FidelityHL[ , row := seq_len(.N), by = Construct]
es_FidelityHL <- full_join(es_FidelityHL, FidelityHL_k)
es_FidelityHL$rirr  <- logRIRR2RIRR(es_FidelityHL$pred)
es_FidelityHL$lower <- logRIRR2RIRR(es_FidelityHL$ci.lb)
es_FidelityHL$upper <- logRIRR2RIRR(es_FidelityHL$ci.ub)
es_FidelityHL  <- full_join(es_FidelityHL,es_FidelityHL_QB,
                        by=c("Construct"))
es_FidelityHL <- es_FidelityHL[ ,c(1,7,8,9:13,5)]
es_FidelityHL$Construct[es_FidelityHL$row>1] <- ""
es_FidelityHL$QB[es_FidelityHL$row>1] <- ""
es_FidelityHL$QBp[es_FidelityHL$row>1] <- ""
es_FidelityHL$row <- NULL
row.names(es_FidelityHL) <- NULL
es_FidelityHL$QB <- as.numeric(es_FidelityHL$QB)
es_FidelityHL$QBp <- as.numeric(es_FidelityHL$QBp)
es_FidelityHL_html <- kable(es_FidelityHL,
       caption="Mean Percent Change by Intervention (BWC) Fidelity and Construct",
       align=c("l","l","r","r","r","r","r","r"),
       col.names = c("Construct","Fidelity","k","Mean % Change",
                     "Lower CI","Upper CI","Q-Between","p (Q-Between)"),
       digits = c(0,0,0,1,1,1,3,3))
es_FidelityHL_html <-
    footnote(es_FidelityHL_html, general =
      c("Q-Between is the test of the difference between the means."))
write_file(es_FidelityHL_html, "tables/es_moderator_fidelity.html")

##
## Moderator analysis by Reform
##
es.one.per$Reform <- factor(es.one.per$Reform)
table(es.one.per$Construct,es.one.per$Reform)
reform_k <- as.data.frame.matrix(table(es.one.per$Construct,es.one.per$Reform))
reform_k$k <- apply(reform_k[ ,c(1:2)], 1, sum)
reform_k$Construct <- row.names(reform_k)
reform_k <- subset(reform_k, k>2)
constructs <- row.names(reform_k)
names(constructs) <- constructs
es_reform_ma <- lapply(constructs, function(x) {
    rma.uni(lgrirr ~ Reform, vlgrirr, data=es.one.per[es.one.per$Construct==x, ],
            method="REML", slab=AuthorLabel) })
es_reform_QB <- lapply(es_reform_ma, function(x) { x$QM })
es_reform_QBp <- lapply(es_reform_ma, function(x) { x$QMp })
## Create Table of Results for Above Moderator Analysis
es_reform_QB <-  as.data.frame(unlist(es_reform_QB))
es_reform_QBp <-  as.data.frame(unlist(es_reform_QBp))
es_reform_QB <- cbind(es_reform_QB,es_reform_QBp)
es_reform_QB <- round(es_reform_QB,3)
names(es_reform_QB) <- c("QB","QBp")
es_reform_QB$Construct <- row.names(es_reform_QB)
## generated mean ES by reform category
es_reform <- lapply(es_reform_ma, function(x) {
#     predict(x, newmods=matrix(c(0,0,1,0,0,1), nrow=3, byrow=TRUE))
       predict(x, newmods=c(0,1))
} )
es_reform <- lapply(es_reform, as.data.frame)
es_reform <- bind_rows(es_reform, .id = "Construct")
es_reform <- es_reform[ ,1:5]
```

```
es_reform <- es_reform[ , -3]

reform_k <- gather(reform_k, reform, freq, "No or not mentioned":"Yes mentioned")
reform_k <- reform_k[ order(reform_k$Construct,reform_k$reform) , ]
reform_k <- reform_k[ reform_k$freq>0, ]
reform_k <- as.data.table(reform_k)
reform_k <- reform_k[ , row := seq_len(.N), by = Construct]
es_reform <- as.data.table(es_reform)
es_reform <- es_reform[ , row := seq_len(.N), by = Construct]
es_reform <- full_join(es_reform, reform_k)
es_reform$rirr  <- logRIRR2RIRR(es_reform$pred)
es_reform$lower <- logRIRR2RIRR(es_reform$ci.lb)
es_reform$upper <- logRIRR2RIRR(es_reform$ci.ub)
es_reform  <- full_join(es_reform,es_reform_QB,
                        by=c("Construct"))
es_reform <- es_reform[ ,c(1,7,8,9:13,5)]
es_reform$Construct[es_reform$row>1] <- ""
es_reform$QB[es_reform$row>1] <- ""
es_reform$QBp[es_reform$row>1] <- ""
es_reform$row <- NULL
row.names(es_reform) <- NULL
es_reform$QB <- as.numeric(es_reform$QB)
es_reform$QBp <- as.numeric(es_reform$QBp)
es_reform
options(knitr.kable.NA = '')
es_reform_html <- kable(es_reform,
       caption="Mean Percent Change Based by Collaborative Reform and Construct",
       align=c("l","l","r","r","r","r","r","r"),
       col.names = c("Construct","Reform","k","Mean % Change",
                     "Lower CI","Upper CI","Q-Between","p (Q-Between)"),
       digits = c(0,0,0,1,1,1,3,3))
es_reform_html <-
    footnote(es_reform_html, general =
      c("Q-Between is the test of the difference between the collaborative reform
means."))
write_file(es_reform_html, "tables/es_moderator_reform.html")


##
## Moderator analysis by Year BWC Phased In
##
bwcyear_k <-
as.data.frame.matrix(table(es.one.per$Construct,es.one.per$BWCPhasedInYear))
bwcyear_k
constructs <- row.names(bwcyear_k)
names(constructs) <- constructs
es_bwcyear_ma <- lapply(constructs, function(x) {
    rma.uni(lgrirr ~ BWCPhasedInYear, vlgrirr,
data=es.one.per[es.one.per$Construct==x, ],
            method="REML", slab=AuthorLabel) })
beta    <- as.data.frame(unlist(lapply(es_bwcyear_ma, function(x) { x$beta[2] })))
betalb  <- as.data.frame(unlist(lapply(es_bwcyear_ma, function(x) { x$ci.lb[2] })))
betaup  <- as.data.frame(unlist(lapply(es_bwcyear_ma, function(x) { x$ci.ub[2] })))
pz       <- as.data.frame(unlist(lapply(es_bwcyear_ma, function(x) { x$pval[2] })))
z       <- as.data.frame(unlist(lapply(es_bwcyear_ma, function(x) { x$zval[2] })))
bwcyear <- cbind(beta, betalb, betaup, z, pz)
colnames(bwcyear) <- c("b","lb","ub","z","p")
bwcyear <- round(bwcyear,3)
bwcyear$Construct <- row.names(bwcyear)
rownames(bwcyear) <- NULL
bwcyear <- bwcyear[ ,c(6,1:5)]
```

```
bwcyear
bwcyear_html <- kable(bwcyear,
       caption="Meta-Regression for Year BWC Phased In by Construct Predicting logged
RIRR",
       align=c("r","r","r","r","r"),
       col.names = c("Construct","Regression Coefficient","Lower 95%","Upper
95%","z","p"))
write_file(bwcyear_html, "tables/es_moderator_bwceyar.html")

## ---------------------------------------------------------------------
##
## Sensitivity Analyses
##
## ---------------------------------------------------------------------
##
## Drop TS analyses
##
es.one.per <- tmp
keep <- as.data.frame(table(es.one.per$Construct[es.one.per$Design=="QE: time
series"]))
keep <- keep$Var1[keep$Freq>0]
es.one.per <- subset(es.one.per, !(Design=="QE: time series"))
es.one.per <- subset(es.one.per, Construct %in% keep)
es.one.per$Construct <- factor(es.one.per$Construct)
constructs <- as.list(levels(es.one.per$Construct))
names(constructs) <- levels(es.one.per$Construct)
es_tbl2_ma <- lapply(constructs, meanES)
es_tbl2    <- lapply(es_tbl2_ma, extractResults)
es_tbl2 <- matrix(unlist(es_tbl2,
                          recursive=FALSE, use.names=FALSE), ncol=9, byrow=TRUE)
es_tbl2 <- as.data.frame(es_tbl2)
names(es_tbl2) <- c("Mean % Change","Lower CI","Upper CI",
                     "k","z","p","Q","p of Q","tau")
es_tbl2$Construct <- unlist(constructs)
es_tbl2 <- es_tbl2[ ,c(10,1:9)]
es_tbl2$Q[es_tbl2$k==1] <- NA
es_tbl2$"p of Q"[es_tbl2$k==1] <- NA
es_tbl2$tau[es_tbl2$k==1] <- NA
es_tbl2 <- es_tbl2[order(-es_tbl2$k),]
row.names(es_tbl2) <- NULL
es_tbl2_html <- kable(es_tbl2,
       caption="Overall Mean Percent Change by
                Construct and Associated Statistics without Time Series Designs",
       align=c("l","r","r","r","r","r","r","r","r","r"),
       col.names = c("Construct","Mean % Change","Lower CI","Upper CI",
                     "k","z","p","Q","p","tau"))
es_tbl2_html <-
    footnote(es_tbl2_html, general =
    c("Percent change based on Relative Incident Rate Ratio (RIRR). For values of RIRR
greater than 1, the percent change is RIRR minus 1. For values of RIRR less than one
it is 1 minus RIRR. A RIRR reflects the difference-in-difference percent change in
counts. Negative numbers reflect lower counts for the BWC condition."))
write_file(es_tbl2_html, "tables/es_tbl2.html")
es.one.per <- tmp

##
## Dropping
##   Mesa PD, Ready and Young (2013, 2015) MESA, AZ
##
es.one.per <- tmp
keep <- as.data.frame(table(es.one.per$Construct[es.one.per$StudyID=="S314"]))
```

```
keep <- keep$Var1[keep$Freq>0]
es.one.per <- subset(es.one.per, !(StudyID=="S314" | StudyID=="S613"))
es.one.per <- subset(es.one.per, Construct %in% keep)
es.one.per$Construct <- factor(es.one.per$Construct)
constructs <- as.list(levels(es.one.per$Construct))
names(constructs) <- levels(es.one.per$Construct)
es_tbl3_ma <- lapply(constructs, meanES)
es_tbl3    <- lapply(es_tbl3_ma, extractResults)
es_tbl3 <- matrix(unlist(es_tbl3,
                          recursive=FALSE, use.names=FALSE), ncol=9, byrow=TRUE)
es_tbl3 <- as.data.frame(es_tbl3)
names(es_tbl3) <- c("Mean % Change","Lower CI","Upper CI",
                     "k","z","p","Q","p of Q","tau")
es_tbl3$Construct <- constructs
es_tbl3 <- es_tbl3[ ,c(10,1:9)]
es_tbl3$Q[es_tbl3$k==1] <- NA
es_tbl3$"p of Q"[es_tbl3$k==1] <- NA
es_tbl3$tau[es_tbl3$k==1] <- NA
es_tbl3 <- es_tbl3[order(-es_tbl3$k),]
row.names(es_tbl3) <- NULL
es_tbl3_html <- kable(es_tbl3,
       caption="Overall Mean Percent Change by
                Construct and Associated Statistics without Mesa PD (2013, 2015)
Study",
       align=c("l","r","r","r","r","r","r","r","r","r"),
       col.names = c("Construct","Mean % Change","Lower CI","Upper CI",
                     "k","z","p","Q","p","tau"))
es_tbl3_html <-
    footnote(es_tbl3_html, general =
    c("Percent change based on Relative Incident Rate Ratio (RIRR). For values of RIRR
greater than 1, the percent change is RIRR minus 1. For values of RIRR less than one
it is 1 minus RIRR. A RIRR reflects the difference-in-difference percent change in
counts. Negative numbers reflect lower counts for the BWC condition."))
write_file(es_tbl3_html, "tables/es_tbl3.html")
es.one.per <- tmp

##
## Dropping
##   Ariel Global 10 Studies
##

##
es.one.per <- tmp
keep <- as.data.frame(table(es.one.per$Construct[es.one.per$StudyID=="S274"]))
keep <- keep$Var1[keep$Freq>0]
es.one.per <- subset(es.one.per, !(StudyID=="S274"))
es.one.per <- subset(es.one.per, Construct %in% keep)
es.one.per$Construct <- factor(es.one.per$Construct)
constructs <- as.list(levels(es.one.per$Construct))
names(constructs) <- levels(es.one.per$Construct)
es_tbl4_ma <- lapply(constructs, meanES)
es_tbl4    <- lapply(es_tbl4_ma, extractResults)
es_tbl4 <- matrix(unlist(es_tbl4,
                          recursive=FALSE, use.names=FALSE), ncol=9, byrow=TRUE)
es_tbl4 <- as.data.frame(es_tbl4)
names(es_tbl4) <- c("Mean % Change","Lower CI","Upper CI",
                     "k","z","p","Q","p of Q","tau")
es_tbl4$Construct <- constructs
es_tbl4 <- es_tbl4[ ,c(10,1:9)]
es_tbl4$Q[es_tbl4$k==1] <- NA
es_tbl4$"p of Q"[es_tbl4$k==1] <- NA
```

```
es_tbl4$tau[es_tbl4$k==1] <- NA
es_tbl4 <- es_tbl4[order(-es_tbl4$k),]
row.names(es_tbl4) <- NULL
es_tbl4_html <- kable(es_tbl4,
       caption="Overall Mean Percent Change by
                Construct and Associated Statistics without Ariel et al. Global 10
Studies",
       align=c("l","r","r","r","r","r","r","r","r","r"),
       col.names = c("Construct","Mean % Change","Lower CI","Upper CI",
                     "k","z","p","Q","p","tau"))
es_tbl4_html <-
    footnote(es_tbl4_html, general =
    c("Percent change based on Relative Incident Rate Ratio (RIRR). For values of RIRR
greater than 1, the percent change is RIRR minus 1. For values of RIRR less than one
it is 1 minus RIRR. A RIRR reflects the difference-in-difference percent change in
counts. Negative numbers reflect lower counts for the BWC condition."))
write_file(es_tbl4_html, "tables/es_tbl4.html")
es.one.per <- tmp

## ------------------------------------------------------------------------
##
## Publication selection bias exploration (Complaints and Use of Force)
##
## ------------------------------------------------------------------------
ma_complaints <- rma.uni(lgrirr, vlgrirr,
                         data=es.one.per[es.one.per$Construct=="Complaints Against
Officer", ],
                         method="REML", slab=AuthorLabel)
ma_uof        <- rma.uni(lgrirr, vlgrirr,
                         data=es.one.per[es.one.per$Construct=="Use of Force", ],
                         method="REML", slab=AuthorLabel)
tf_ma_complaints  <- trimfill(ma_complaints)
tf_ma_uof         <- trimfill(ma_uof)
ma_complaints
tf_ma_complaints
ma_uof
tf_ma_uof
svglite("./figures/funnel_complaints.svg")
funnel(tf_ma_complaints, legend=TRUE)
dev.off()
svglite("./figures/funnel_useofforce.svg")
funnel(tf_ma_uof, legend=TRUE)
dev.off()
regtest(ma_complaints)
regtest(ma_uof)

es.one.per <- es.one.per %>%
    dplyr::mutate(
              Published = case_when(
                  PubType=="Book" ~ "Yes",
                  PubType=="Journal" ~ "Yes",
                  TRUE ~ "No" ))
ma_complaints <- rma.uni(lgrirr ~ Published, vlgrirr,
                         data=es.one.per[es.one.per$Construct=="Complaints Against
Officer", ],
                         method="REML", slab=AuthorLabel)
ma_uof        <- rma.uni(lgrirr ~ Published, vlgrirr,
                         data=es.one.per[es.one.per$Construct=="Use of Force", ],
                         method="REML", slab=AuthorLabel)
summary(ma_complaints)
summary(ma_uof)
```

```
predict(ma_complaints, newmods=c(0,1))
predict(ma_uof, newmods=c(0,1))

es.one.per <- es.one.per %>%
    dplyr::mutate(
              Published = case_when(
                  PubType=="Journal" ~ "Yes",
                  PubType=="Book" ~ "Yes",
                  TRUE ~ "No" ))
ma_complaints <- rma.uni(lgrirr ~ Published, vlgrirr,
                         data=es.one.per[es.one.per$Construct=="Complaints Against
Officer", ],
                         method="REML", slab=AuthorLabel)
ma_uof        <- rma.uni(lgrirr ~ Published, vlgrirr,
                         data=es.one.per[es.one.per$Construct=="Use of Force", ],
                         method="REML", slab=AuthorLabel)
summary(ma_complaints)
summary(ma_uof)
predict(ma_complaints, newmods=c(0,1))
predict(ma_uof, newmods=c(0,1))

##
## End code
##
```
