# Supplementary material for: Body‐worn cameras’ effects on police officers and citizen behavior: A systematic review
Source: Campbell Syst Rev. 2020 Sep 9;16(3):e1112. doi: 10.1002/cl2.1112 (PMC8356344; doi:10.1002/cl2.1112)
Supplement: Supplementary file 6 — Supporting information [file CL2-16-e1112-s002.pdf]

APPENDIX G: STUDY LEVEL RISK-OF-BIAS (SLIGHTLY MODIFIED COCHRANE TOOL)

Part of: Lum, C., Koper, C.S., Wilson, D.B., …et al. (2020). Body-worn cameras' effects on police officers and citizen behavior: A systematic review. Campbell Systematic Reviews 2020;e1112. https://doi.org/10.1002/cl2.1112.

	Domain 1: Risk of bias arising from the randomization process					Domain 2: Risk of bias due to deviations from the intended interventions		Domain 5: Risk of bias in selection of the reported result		
**Study Name**	**1.1**	**1.3**	**1 Extra**	**1 Cont.**	**1 Opt**	**2.6**	**2.7**	**5.1**	**5.2**	**5.3**
Ariel (2016, 2017) DENVER, CO	N	Y	N	NI	Unpredictable	Y	N	NI	NI	NI
Ariel et al. (2016, 2017, 2018) SITE A	Y	N	PY	PY	Unpredictable	NI	NI	Y	NI	NI
Ariel et al. (2016, 2017, 2018) SITE B	Y	N	PN	PY	Towards the null	NI	NI	Y	NI	NI
Ariel et al. (2016, 2017, 2018) SITE C	Y	N	PY	PY	Unpredictable	NI	NI	Y	NI	NI
Ariel et al. (2016, 2017, 2018) SITE D	Y	N	N	PY	Towards the null	NI	NI	Y	NI	NI
Ariel et al. (2016, 2017, 2018) SITE E	Y	N	PY	PY	Unpredictable	NI	NI	Y	NI	NI
Ariel et al. (2016, 2017, 2018) SITE F	Y	N	PY	PY	Unpredictable	NI	NI	Y	NI	NI
Ariel et al. (2016, 2017, 2018) SITE H	Y	N	PN	PY	Towards the null	NI	NI	Y	NI	NI
Ariel et al. (2016, 2017, 2018) SITE I	Y	N	PY	PY	Towards the null	NI	NI	Y	NI	NI
Ariel et al. (2016, 2017, 2018) SITE J	Y	N	PY	PY	Towards the null	NI	NI	Y	NI	NI
Ariel et al. (2016, 2017, 2018) SITE K	Y	N	PY	PY	Towards the null	NI	NI	Y	NI	NI
Jennings et al. (2017) TAMPA, FL	N	N	NI	PY	Towards the null	Y	N	NI	NI	NI
Ariel, Farrar, et al. (2012, 2013, 2015, 2017) RIALTO, CA	Y	N	N	Y	Towards the null	Y	N	Y	N	N
Jennings et al. (2015) ORLANDO, FL	Y	N	NI	PY	Towards the null	Y	N	NI	NI	NI
Mesa PD, Ready and Young (2013, 2015) MESA, AZ	N	N	NI	PY	Unpredictable	Y	N	NI	NI	NI
Sousa, Braga, et al. (2016, 2018) LAS VEGAS, NV	Y	N	N	Y	Towards the null	Y	N	NI	NI	NI
Headley et al. (2017) HALLANDALE BEACH, FL	N	NI	N	Y	Unpredictable	Y	N	NI	NI	NI

	Domain 1: Risk of bias arising from the randomization process					Domain 2: Risk of bias due to deviations from the intended interventions		Domain 5: Risk of bias in selection of the reported result		
**Study Name**	**1.1**	**1.3**	**1 Extra**	**1 Cont.**	**1 Opt**	**2.6**	**2.7**	**5.1**	**5.2**	**5.3**
Henstock and Ariel (2017) WEST MIDLANDS, UK	Y	N	Y	Y	Towards the null	Y	N	Y	NI	NI
Mitchell et al. (2018) URUGUAY	N	Y	PN	N	Unpredictable	Y	N	NI	NI	NI
White et al. (2018) SPOKANE, WA	Y	N	Y	Y	Towards the null	PN	N	NI	NI	NI
Wallace et al. (2018) SPOKANE, WA	Y	N	Y	Y	Towards the null	PN	PN	NI	NI	NI
Braga et al. (2019) BOSTON, MA	Y	N	NI	Y	Towards the null	Y	N	NI	NI	NI
Grossmith, Owens, Finn, et al. (2015, 2018) LONDON, UK	Y	PN	N	PY	Towards the null	Y	N	NI	NI	NI
Katz et al. (2015, 2016) PHOENIX, AZ (Maryvale)	N	Y	NI	Y	Towards the null	Y	N	NI	NI	NI
Peterson, Lawrence, et al. (2018, 2019) MILWAUKEE, WI	Y	N	NI	PY	Towards the null	Y	N	NI	NI	NI
Stolzenberg et al. (2019) MIAMI-DADE, FL	N	N	N	N		Y	N	N	N	N
Koslicki et al. (2019) NORTHWEST CITY	N	N	N	N		Y	N	N	N	N
Yokum et al. (2019) WASHINGTON, DC	Y	N	PN	Y	Towards the null	Y	N	Y	N	N
Bennett et al. (2019) FAIRFAX COUNTY, VA	N	PN	N	N	Unpredictable	Y	NI	NI	NI	NI
Katz et al. (2019) PHOENIX, AZ (not Maryvale/Mandated)	Y	N	N	PY	Unpredictable	Y	N	NI	N	N
Katz et al. (2019) PHOENIX, AZ (not Maryvale/Volunteer)	N	Y	N	PY	Unpredictable	Y	N	NI	N	N

Notes:

Y=Yes; PY=Probably Yes; PN=Probably No; N=No; NI=No information

1.1: Was the allocation sequence random?

1.3: Did baseline differences between intervention groups suggest a problem with the randomization [or other selection] process?

	Domain 1: Risk of bias arising from the randomization process					Domain 2: Risk of bias due to deviations from the intended interventions		Domain 5: Risk of bias in selection of the reported result		
**Study Name**	**1.1**	**1.3**	**1 Extra**	**1 Cont.**	**1 Opt**	**2.6**	**2.7**	**5.1**	**5.2**	**5.3**

1 Extra: Were there violations to the randomization process?

1 Cont.: Was there contamination between the treatment and control conditions?

1 Opt.: What is the predicted direction of bias arising from the randomization [or other selection] process?

2.6: Was an appropriate analysis used to estimate the effect of assignment to intervention?

2.7: Was there potential for a substantial impact (on the result) of the failure to analyze participants in the group to which they were randomized?

5.1: Were the data that produced this result [the results for this study] analyzed in accordance with a pre-specified analysis plan that was finalized before unblinded outcome data were available for analysis?

5.2: Is (Are) the numerical result [results] being assessed likely to have been selected, on the basis of the results, from multiple outcome measurements?

5.3: Is (Are) the numerical result [results] being assessed likely to have been selected, on the basis of the results, from multiple analyses of the data?
