# Supplementary material for: Body‐worn cameras’ effects on police officers and citizen behavior: A systematic review
Source: Campbell Syst Rev. 2020 Sep 9;16(3):e1112. doi: 10.1002/cl2.1112 (PMC8356344; doi:10.1002/cl2.1112)
Supplement: Supplementary file 7 — Supporting information [file CL2-16-e1112-s003.pdf]

APPENDIX H. OUTCOME/DEPENDENT VARIABLE LEVEL RISK-OF-BIAS (SLIGHTLY MODIFIED COCHRANE TOOL)

Part of: Lum, C., Koper, C.S., Wilson, D.B., …et al. (2020). Body-worn cameras' effects on police officers and citizen behavior: A systematic review. Campbell Systematic Reviews 2020;e1112. https://doi.org/10.1002/cl2.1112.

	Domain 3: Missing outcome data		Domain 4: Risk of bias in measurement			
**Construct and Study Name**	**3.1**	**3.2**	**4.2**	**4.3**	**4.4**	**4.5**
**Arrests**						
Ariel (2016, 2017) DENVER, CO	Yes		No	Yes	No	
Braga et al. (2019) BOSTON, MA	Yes		No	Probably Yes	Probably Yes	Probably No
Grossmith, Owens, Finn, et al. (2015, 2018) LONDON, UK	Yes		No	Probably No		
Headley et al. (2017) HALLANDALE BEACH, FL	Probably Yes		No	No		
Katz et al. (2015, 2016) PHOENIX, AZ (Maryvale)	Probably Yes		No	Yes	Probably No	
Katz et al. (2019) PHOENIX, AZ (not Maryvale/Mandated)	Yes		No	No		
Katz et al. (2019) PHOENIX, AZ (not Maryvale/Volunteer)	Yes		No	No		
Mesa PD, Ready and Young (2013, 2015) MESA, AZ	No information	No information	Probably Yes			
Peterson, Lawrence, et al. (2018, 2019) MILWAUKEE, WI	Yes		No	Yes	Probably Yes	Probably No
Sousa, Braga, et al. (2016, 2018) LAS VEGAS, NV	Yes		No	Yes	No	
Stolzenberg et al. (2019) MIAMI-DADE, FL	Yes		No	No		
Wallace et al. (2018) SPOKANE, WA	Yes		No	No		
Yokum et al. (2019) WASHINGTON, DC	Yes		No	Yes	No	
**Citations**						
Mesa PD, Ready and Young (2013, 2015) MESA, AZ	No information	No information	Probably Yes			
Sousa, Braga, et al. (2016, 2018) LAS VEGAS, NV	Yes		No	Yes	No	
**Complaints Against Officer**						
Ariel (2016, 2017) DENVER, CO	Yes		Probably Yes			
Ariel et al. (2016, 2017, 2018) SITE A	Probably Yes		Probably Yes			
Ariel et al. (2016, 2017, 2018) SITE B	Probably Yes		Probably Yes			
Ariel et al. (2016, 2017, 2018) SITE C	Probably Yes		Probably Yes			
Ariel et al. (2016, 2017, 2018) SITE E	Probably Yes		Probably Yes			
Ariel et al. (2016, 2017, 2018) SITE H	Probably Yes		Probably Yes			

Construct and Study Name	Domain 3: Missing outcome data		Domain 4: Risk of bias in measurement			
	3.1	3.2	4.2	4.3	4.4	4.5
Ariel et al. (2016, 2017, 2018) SITE K	Probably Yes		Probably Yes			
Ariel, Farrar, et al. (2012, 2013, 2015, 2017) RIALTO, CA	Probably Yes		Probably Yes			
Bennett et al. (2019) FAIRFAX COUNTY, VA	Probably Yes		Probably Yes			
Braga et al. (2019) BOSTON, MA	Yes		Probably Yes			
Grossmith, Owens, Finn, et al. (2015, 2018) LONDON, UK	Yes		Probably Yes			
Headley et al. (2017) HALLANDALE BEACH, FL	Probably Yes		Probably Yes			
Jennings et al. (2015) ORLANDO, FL	Probably Yes		Probably Yes			
Katz et al. (2015, 2016) PHOENIX, AZ (Maryvale)	Probably Yes		Probably Yes			
Katz et al. (2019) PHOENIX, AZ (not Maryvale/Mandated)	Yes		Probably Yes			
Katz et al. (2019) PHOENIX, AZ (not Maryvale/Volunteer)	Yes		Probably Yes			
Mitchell et al. (2018) URUGUAY	Yes		Probably Yes			
Peterson, Lawrence, et al. (2018, 2019) MILWAUKEE, WI	Yes		Probably Yes			
Sousa, Braga, et al. (2016, 2018) LAS VEGAS, NV	Yes		Probably Yes			
Stolzenberg et al. (2019) MIAMI-DADE, FL	Yes		Probably Yes			
White et al. (2018) SPOKANE, WA	Yes		Probably Yes			
Yokum et al. (2019) WASHINGTON, DC	Yes		Probably Yes			
**Dispatched Calls for Service**						
Ariel (2016, 2017) DENVER, CO	Yes		No	Yes	No	
Bennett et al. (2019) FAIRFAX COUNTY, VA	Probably Yes		No	No		
Braga et al. (2019) BOSTON, MA	Yes		No	No		
Katz et al. (2019) PHOENIX, AZ (not Maryvale/Mandated)	Yes		No	No		
Katz et al. (2019) PHOENIX, AZ (not Maryvale/Volunteer)	Yes		No	No		
Sousa, Braga, et al. (2016, 2018) LAS VEGAS, NV	Yes		No	Yes	No	
**Incident Reports**						
Braga et al. (2019) BOSTON, MA	Yes		No	No		
Sousa, Braga, et al. (2016, 2018) LAS VEGAS, NV	Yes		No	Yes	No	
Stolzenberg et al. (2019) MIAMI-DADE, FL	Yes		No	No		
**Officer Initiated CFS**						
Braga et al. (2019) BOSTON, MA	Yes		Probably Yes			

Construct and Study Name	Domain 3: Missing outcome data		Domain 4: Risk of bias in measurement			
	3.1	3.2	4.2	4.3	4.4	4.5
Headley et al. (2017) HALLANDALE BEACH, FL	Probably Yes		Probably Yes			
Katz et al. (2019) PHOENIX, AZ (not Maryvale/Mandated)	Yes		Probably Yes			
Katz et al. (2019) PHOENIX, AZ (not Maryvale/Volunteer)	Yes		Probably Yes			
Mesa PD, Ready and Young (2013, 2015) MESA, AZ	No information	No information	Probably Yes			
Peterson, Lawrence, et al. (2018, 2019) MILWAUKEE, WI	Yes		Probably Yes			
Sousa, Braga, et al. (2016, 2018) LAS VEGAS, NV	Yes		Probably Yes			
Wallace et al. (2018) SPOKANE, WA	Yes		Probably Yes			
**Assault on Officer/Officer Injuries/Resistance**						
Ariel et al. (2016, 2017, 2018) SITE A	Probably Yes		Probably Yes			
Ariel et al. (2016, 2017, 2018) SITE B	Probably Yes		Probably Yes			
Ariel et al. (2016, 2017, 2018) SITE C	Probably Yes		Probably Yes			
Ariel et al. (2016, 2017, 2018) SITE D	Probably Yes		Probably Yes			
Ariel et al. (2016, 2017, 2018) SITE E	Probably Yes		Probably Yes			
Ariel et al. (2016, 2017, 2018) SITE F	Probably Yes		Probably Yes			
Ariel et al. (2016, 2017, 2018) SITE H	Probably Yes		Probably Yes			
Ariel et al. (2016, 2017, 2018) SITE I	Probably Yes		Probably Yes			
Ariel et al. (2016, 2017, 2018) SITE J	Probably Yes		Probably Yes			
Ariel et al. (2016, 2017, 2018) SITE K	Probably Yes		Probably Yes			
Headley et al. (2017) HALLANDALE BEACH, FL	Probably Yes		Probably Yes			
Katz et al. (2015, 2016) PHOENIX, AZ (Maryvale)	Probably Yes		Probably Yes			
Stolzenberg et al. (2019) MIAMI-DADE, FL	Yes		Probably Yes			
White et al. (2018) SPOKANE, WA	Probably Yes		Probably Yes			
Yokum et al. (2019) WASHINGTON, DC	Probably Yes		Probably Yes			
**Response Time**						
Katz et al. (2019) PHOENIX, AZ (not Maryvale/Mandated)	Yes		No	No		
Katz et al. (2019) PHOENIX, AZ (not Maryvale/Volunteer)	Yes		No	No		
Wallace et al. (2018) SPOKANE, WA	Yes		No	No		
**Stop and Frisk**						
Braga et al. (2019) BOSTON, MA	Yes		Probably Yes			

	Domain 3: Missing outcome data		Domain 4: Risk of bias in measurement			
**Construct and Study Name**	**3.1**	**3.2**	**4.2**	**4.3**	**4.4**	**4.5**
Grossmith, Owens, Finn, et al. (2015, 2018) LONDON, UK	Yes		Probably Yes			
Mesa PD, Ready and Young (2013, 2015) MESA, AZ	No information	No information	Probably Yes			
Peterson, Lawrence, et al. (2018, 2019) MILWAUKEE, WI	Yes		Probably Yes			
**Time on Scene**						
Wallace et al. (2018) SPOKANE, WA	Yes		No	No		
**Traffic Stops**						
Bennett et al. (2019) FAIRFAX COUNTY, VA	Probably Yes		Probably Yes			
Headley et al. (2017) HALLANDALE BEACH, FL	Probably Yes		Probably Yes			
Peterson, Lawrence, et al. (2018, 2019) MILWAUKEE, WI	Yes		Probably Yes			
Stolzenberg et al. (2019) MIAMI-DADE, FL	Yes		Probably Yes			
Yokum et al. (2019) WASHINGTON, DC	Yes		Probably Yes			
**Use of Force**						
Ariel (2016, 2017) DENVER, CO	Yes		Probably Yes			
Ariel et al. (2016, 2017, 2018) SITE A	Probably Yes		Probably Yes			
Ariel et al. (2016, 2017, 2018) SITE B	Probably Yes		Probably Yes			
Ariel et al. (2016, 2017, 2018) SITE C	Probably Yes		Probably Yes			
Ariel et al. (2016, 2017, 2018) SITE D	Probably Yes		Probably Yes			
Ariel et al. (2016, 2017, 2018) SITE E	Probably Yes		Probably Yes			
Ariel et al. (2016, 2017, 2018) SITE F	Probably Yes		Probably Yes			
Ariel et al. (2016, 2017, 2018) SITE H	Probably Yes		Probably Yes			
Ariel et al. (2016, 2017, 2018) SITE I	Probably Yes		Probably Yes			
Ariel et al. (2016, 2017, 2018) SITE J	Probably Yes		Probably Yes			
Ariel et al. (2016, 2017, 2018) SITE K	Probably Yes		Probably Yes			
Ariel, Farrar, et al. (2012, 2013, 2015, 2017) RIALTO, CA	Probably Yes		Probably Yes			
Bennett et al. (2019) FAIRFAX COUNTY, VA	Probably Yes		Probably Yes			
Braga et al. (2019) BOSTON, MA	Yes		Probably Yes			
Headley et al. (2017) HALLANDALE BEACH, FL	Probably Yes		Probably Yes			
Henstock and Ariel (2017) WEST MIDLANDS, UK	Yes		Probably Yes			
Jennings et al. (2015) ORLANDO, FL	Probably Yes		Probably Yes			

	Domain 3: Missing outcome data		Domain 4: Risk of bias in measurement			
**Construct and Study Name**	**3.1**	**3.2**	**4.2**	**4.3**	**4.4**	**4.5**
Jennings et al. (2017) TAMPA, FL	Probably Yes		Probably Yes			
Katz et al. (2019) PHOENIX, AZ (not Maryvale/Mandated)	Yes		Probably Yes			
Katz et al. (2019) PHOENIX, AZ (not Maryvale/Volunteer)	Yes		Probably Yes			
Koslicki et al. (2019) NORTHWEST CITY	Yes		Probably Yes			
Peterson, Lawrence, et al. (2018, 2019) MILWAUKEE, WI	Yes		Probably Yes			
Sousa, Braga, et al. (2016, 2018) LAS VEGAS, NV	Yes		Probably Yes			
Stolzenberg et al. (2019) MIAMI-DADE, FL	Yes		Probably Yes			
White et al. (2018) SPOKANE, WA	Yes		Probably Yes			
Yokum et al. (2019) WASHINGTON, DC	Yes		Probably Yes			
Yokum et al. (2019) WASHINGTON, DC	Yes		Probably Yes			

Note:

3.1: Were data for this outcome available for all, or nearly all, participants randomized?

3.2: If N/PN/NI to 3.1: Is there evidence that the result was not biased by missing outcome data?

4.2: Could measurement or ascertainment of the outcome have differed between intervention groups?

4.3: If N/PN/NI 4.2: Were outcome assessors aware of the intervention received by study participants?

4.4: If Y/PY/NI to 4.3: Could assessment of the outcome have been influenced by knowledge of intervention received?
