# Supplementary material for: Body‐worn cameras’ effects on police officers and citizen behavior: A systematic review
Source: Campbell Syst Rev. 2020 Sep 9;16(3):e1112. doi: 10.1002/cl2.1112 (PMC8356344; doi:10.1002/cl2.1112)
Supplement: Supplementary file 8 — Supporting information [file CL2-16-e1112-s008.pdf]

APPENDIX I. OUTCOME CONSTRUCTS BY EACH STUDY FOR WHICH AN EFFECT SIZE WAS COMPUTED

Part of: Lum, C., Koper, C.S., Wilson, D.B., …et al. (2020). Body-worn cameras' effects on police officers and citizen behavior: A systematic review. Campbell Systematic Reviews 2020;e1112. https://doi.org/10.1002/cl2.1112.

	Arrests	Citations	Complaints Against Officer	Dispatched Calls for Service	Incident Reports	Officer Initiated CFS	Officer Injuries/Assault	Resistance	Response Time	Stop and Frisk	Time on Scene	Traffic Stops	Use of Force
Ariel (2016, 2017) DENVER, CO	x		x	x									x
Ariel et al. (2016, 2017, 2018) SITE A			x				x						x
Ariel et al. (2016, 2017, 2018) SITE B			x				x						x
Ariel et al. (2016, 2017, 2018) SITE C			x				x						x
Ariel et al. (2016, 2017, 2018) SITE D							x						x
Ariel et al. (2016, 2017, 2018) SITE E			x				x						x
Ariel et al. (2016, 2017, 2018) SITE F							x						x
Ariel et al. (2016, 2017, 2018) SITE H			x				x						x
Ariel et al. (2016, 2017, 2018) SITE I							x						x
Ariel et al. (2016, 2017, 2018) SITE J							x						x
Ariel et al. (2016, 2017, 2018) SITE K			x				x						x
Ariel, Farrar, et al. (2012, 2013, 2015, 2017) RIALTO, CA			x										x
Bennett et al. (2019) FAIRFAX COUNTY, VA			x	x								x	x
Braga et al. (2019) BOSTON, MA	x		x	x	x	x				x			x
Grossmith, Owens, Finn, et al. (2015, 2018) LONDON, UK	x		x							x			
Headley et al. (2017) HALLANDALE BEACH, FL	x		x			x	x	x				x	x
Henstock and Ariel (2017) WEST MIDLANDS, UK													x
Jennings et al. (2015) ORLANDO, FL			x										x
Jennings et al. (2017) TAMPA, FL													x
Katz et al. (2015, 2016) PHOENIX, AZ (Maryvale)	x		x					x					

Katz et al. (2019) PHOENIX, AZ (not Maryvale/Mandated)	x		x	x		x			x				x
Katz et al. (2019) PHOENIX, AZ (not Maryvale/Volunteer)	x		x	x		x			x				x
Koslicki et al. (2019) NORTHWEST CITY													x
Mesa PD, Ready and Young (2013, 2015) MESA, AZ	x	x				x				x			
Mitchell et al. (2018) URUGUAY			x										
Peterson, Lawrence, et al. (2018, 2019) MILWAUKEE, WI	x		x			x				x		x	x
Sousa, Braga, et al. (2016, 2018) LAS VEGAS, NV	x	x	x	x	x	x							x
Stolzenberg et al. (2019) MIAMI-DADE, FL	x		x		x		x					x	x
Wallace et al. (2018) SPOKANE, WA	x					x			x		x		
White et al. (2018) SPOKANE, WA			x				x						x
Yokum et al. (2019) WASHINGTON, DC	x		x				x					x	x
